# Supplementary material for: Broadband synergy versus oscillatory redundancy in the visual cortex
Source: Nat Commun. 2026 Apr 28;17:5568. doi: 10.1038/s41467-026-72444-2 (PMC13294381; doi:10.1038/s41467-026-72444-2)
Supplement: Supplementary file 1 — Supplementary Information [file 41467_2026_72444_MOESM1_ESM.pdf]

Supplementary Figures

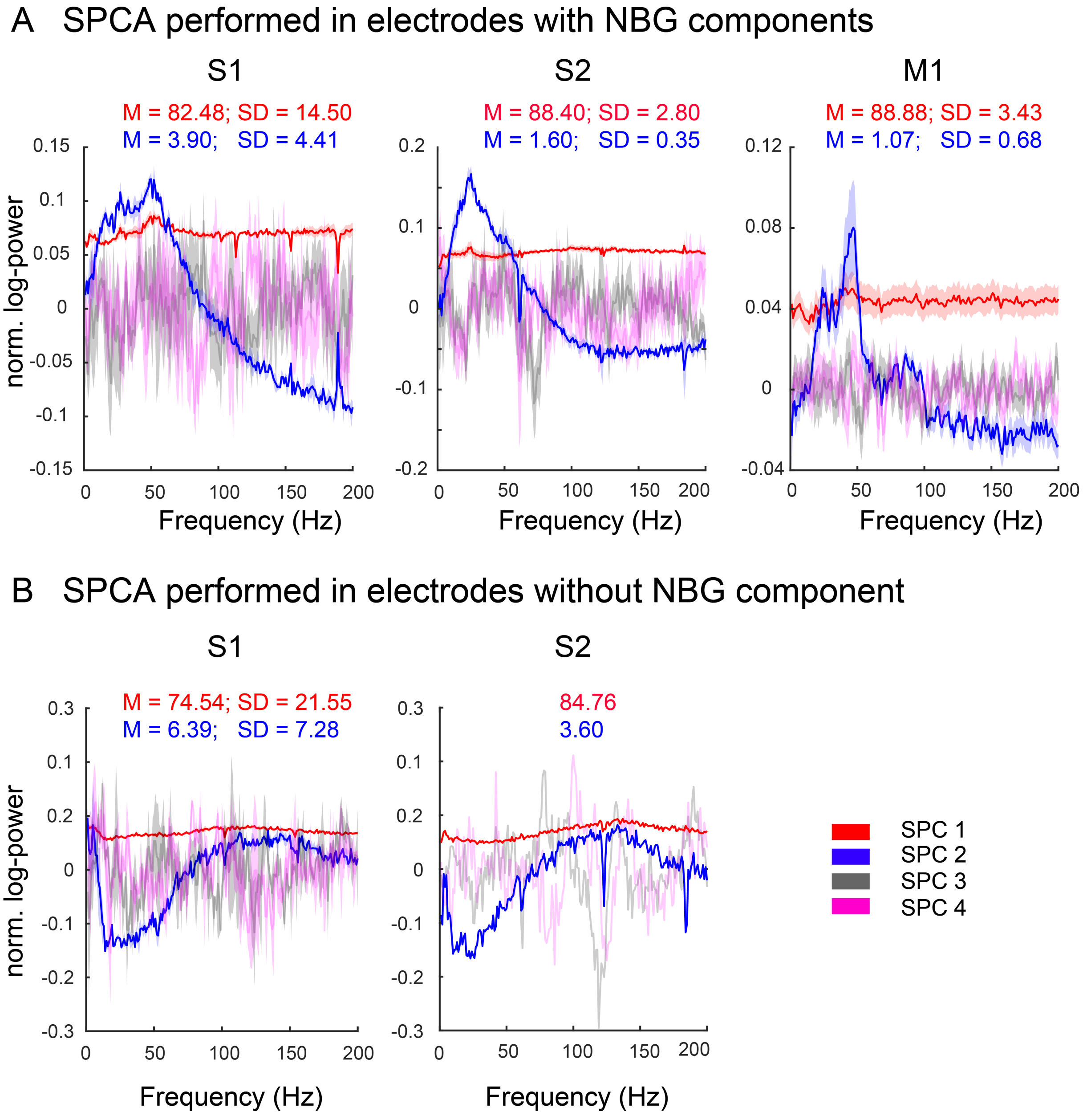

Figure S1: SPCA component for S1, S2, and M1. **(A)** Averaged traces showing a characteristic NBG component for S1 (4 out of 7 electrodes), S2 (5 out of 6 electrodes), and M1 (63 out of 63 electrodes). While SPC1 (red) does not show a characteristic peak across frequencies (BB component), SPCA2 (blue) shows increased magnitude spanning the gamma band (NBG). The mean and standard deviation of the variance explained by the SPC1 and SPC2 components across channels are depicted in red and blue, respectively. Unlike SPC1 and SPC2, SPC3 (gray) and SPC4 (magenta) exhibit high channel variability without a characteristic peak. **(B)** Averaged traces for electrodes without a characteristic NBG component for S1 (2 out of 7 electrodes), S2 (1 out of 6 electrodes). The mean and standard deviation of the variance explained by the SPC1 and SPC2 components across channels are depicted in red and blue, respectively. In contrast, SPC1 shows a broadband component, and SPC2 mainly peaks in the lower-frequency range (<10 Hz).

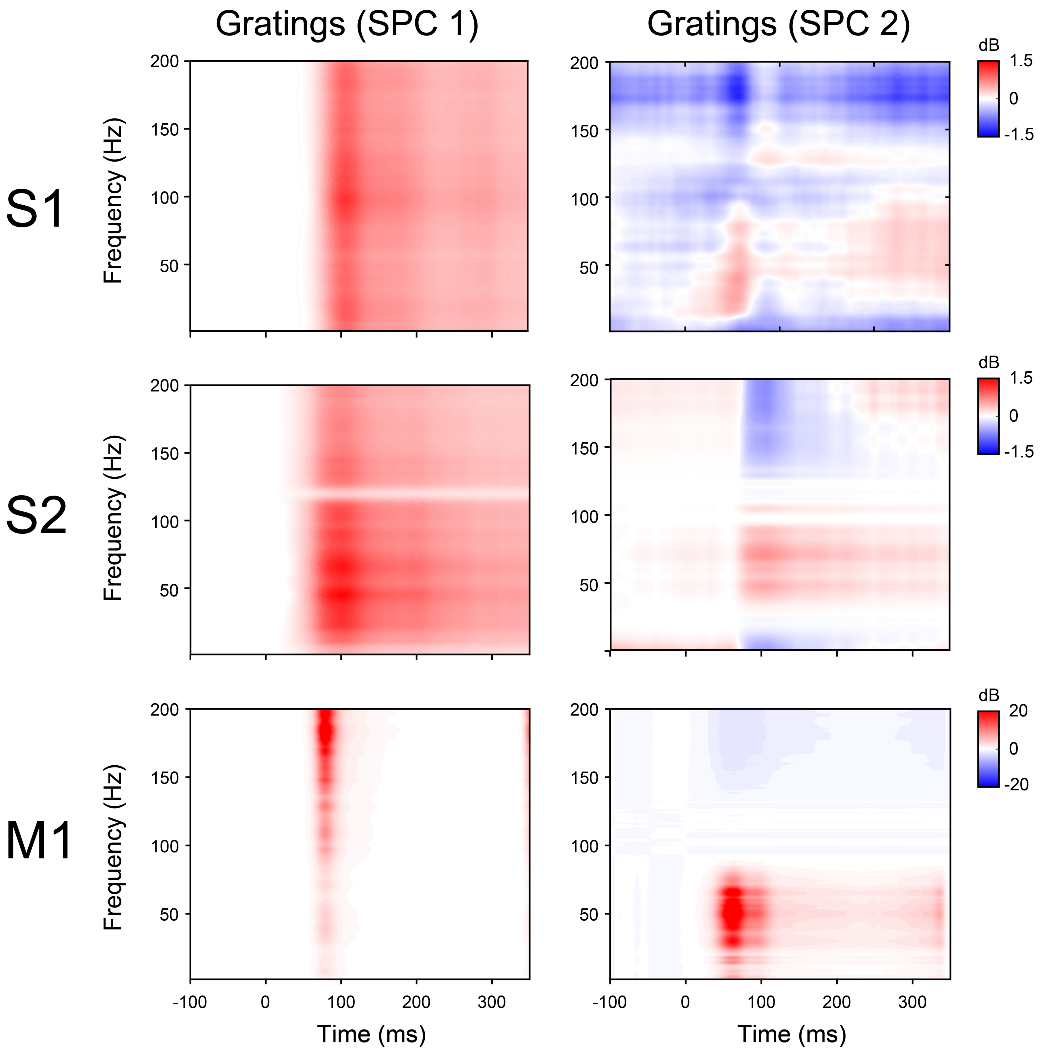

Figure S2: Time–frequency spectral reconstruction using SPC1 and SPC2 in S1, S2, and M1. For each electrode and trial with grating stimuli, Welch power spectra were computed with a single full-length segment (nfft = 500), retaining positive-frequency bins up to 200 Hz. To capture spectral structure, spectra were log-normalized at each frequency, and spectral PCA (SPCA) was performed on the frequency–frequency covariance matrix. The first two components (SPC1 and SPC2) were treated as fixed spectral motifs. We then computed an SPC-specific time course: the signal was filtered with a bank of complex Morlet wavelets (5 cycles per frequency), the squared magnitude was taken to obtain band-power over time, convolution edge samples were discarded, each band's power trace was normalized to unit mean, a natural-log transform was applied, and the resulting log-power matrix was projected onto the SPC vector to yield a single time course for that component. To reconstruct absolute time–frequency (TF) power, we (1) paired this time course with the component's frequency profile to form a separable log-power map, (2) exponentiated that map to convert it into a multiplicative power modulation, and (3) multiplied, at each frequency, by the electrode mean Welch spectrum to return to absolute power units. We then baseline-normalized per frequency to -100 to 0 ms and reported the values in dB (10 log10 of the baseline ratio). Panels show TF maps averaged across channels. Power is expressed in decibels (dB) relative to the baseline period. While SPC1 spans most of the frequency axis, SPC2 shows non-zero power predominantly in the gamma range (30-80 Hz).

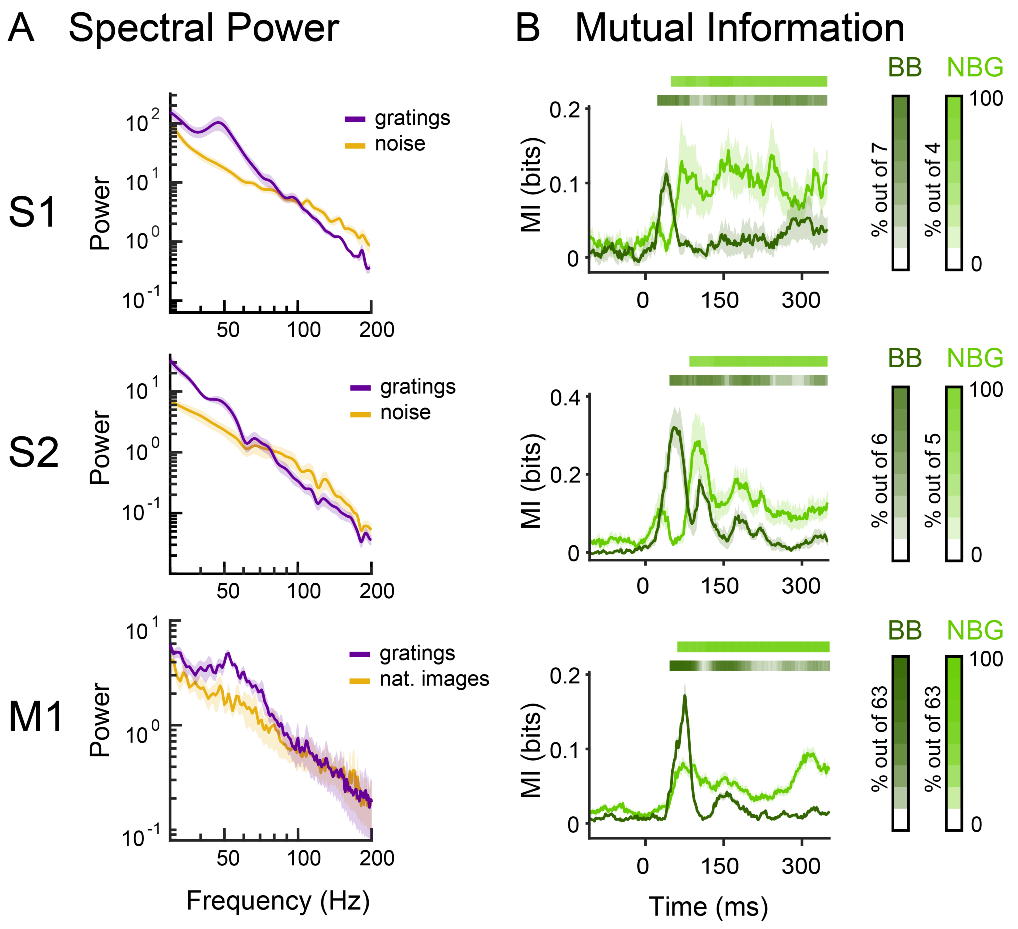

Figure S3: Spectral power and mutual information for S1, S2, and M1. **(A)** Power spectral density for gratings (purple) and noise (yellow) stimuli for S1 and S2, and for gratings and natural images (yellow) for M1. Error bars represents the standard error of the mean (S.E.M) across electrodes (S1: V1,V2; S2: V1, V2, V3, and M1: V1). **(B)** Mutual information analyses between gratings and noise images for the BB component for S1 and S2 (dark green), and between gratings and natural images for M1 (dark green). Mutual information between gratings and noise images for the NBG component for S1 and S2 (light green), and between gratings and natural images for M1 (light green). The shaded horizontal bars represent significant MI values at the single-electrode level, with the colors representing the percentage of electrodes showing a significant effect per time point for BB (dark green) and NBG (light green). Error bars represent the S.E.M of the MI values across electrodes.

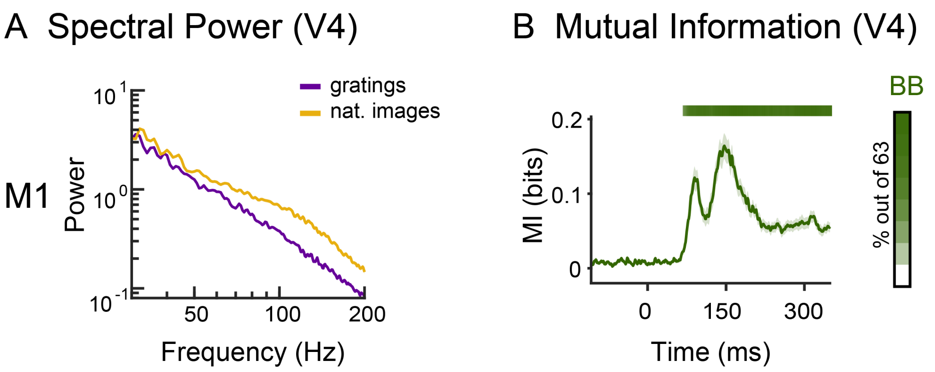

Figure S4: Spectral power and mutual information for M1 in area V4. **(A)** Power spectral density for gratings (purple) and noise and natural images (yellow). Error bars represent the standard error of the mean (S.E.M) across electrodes in V4. **(B)** Mutual information analyses between gratings and natural images for M1 (dark green). The shaded horizontal bars represent significant MI values at the single-electrode level, with the colors representing the percentage of electrodes showing a significant effect per time point for BB (dark green). Error bars represent S.E.M of the MI values across electrodes.

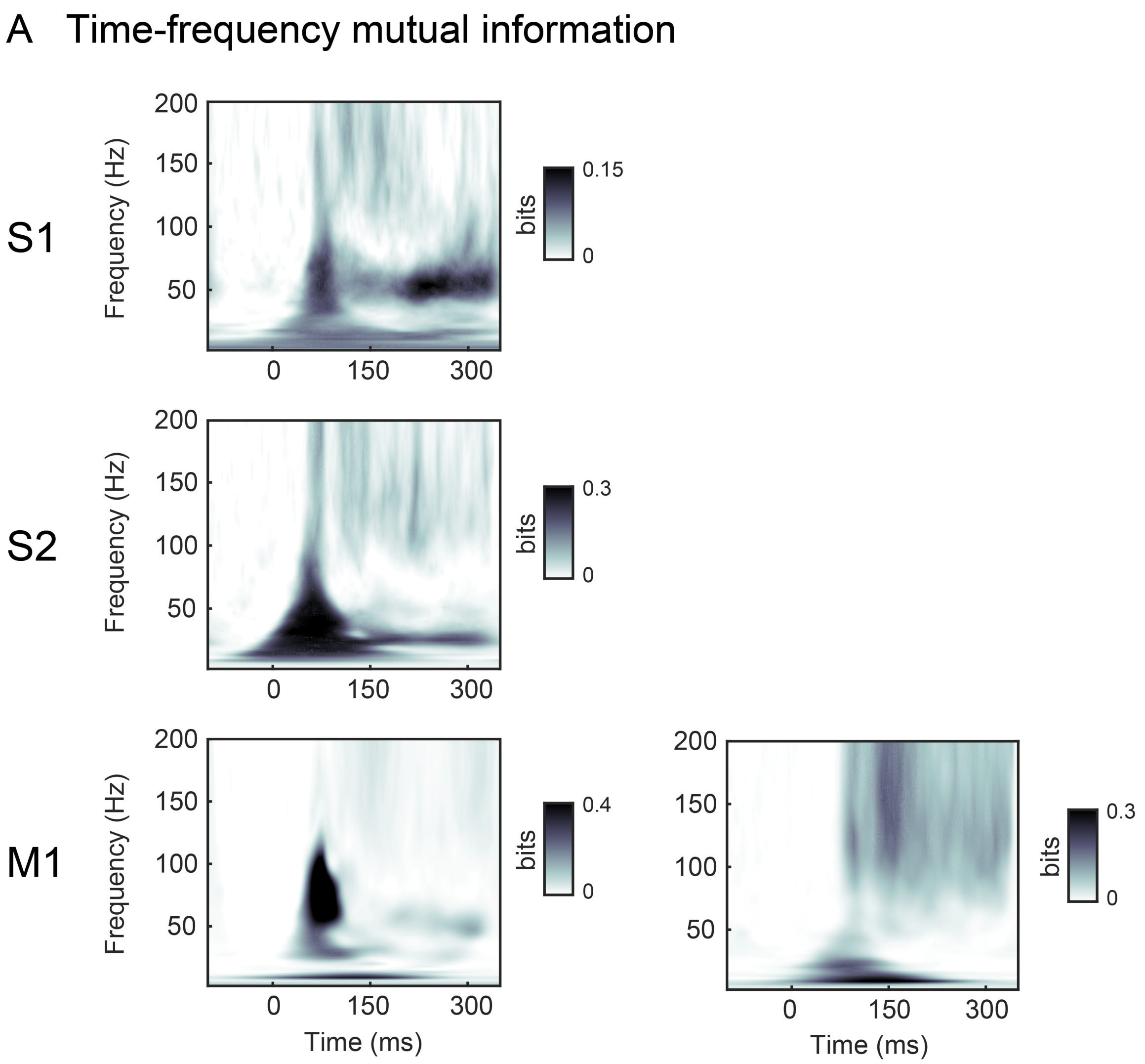

Figure S5: Time-frequency mutual information for S1, S2, and M1. **(A)** Mutual information (MI) between gratings and noise for S1 and S2, and between gratings and natural images for M1. MI was computed from power values across trials for each time-frequency bin from 1 to 200 Hz, within the -100 to 350 ms window around stimulus onset. MI represents the effect size (in bits) of the difference in spectral power for each corresponding stimulus comparison. Plots represent the average MI across electrodes (S1: 7 electrodes [V1 and V2 area]; S2: 6 electrodes [V1, V2 and V3 area], M1: 63 electrodes in V1 (left panel), and 63 electrodes in V4, right panel).

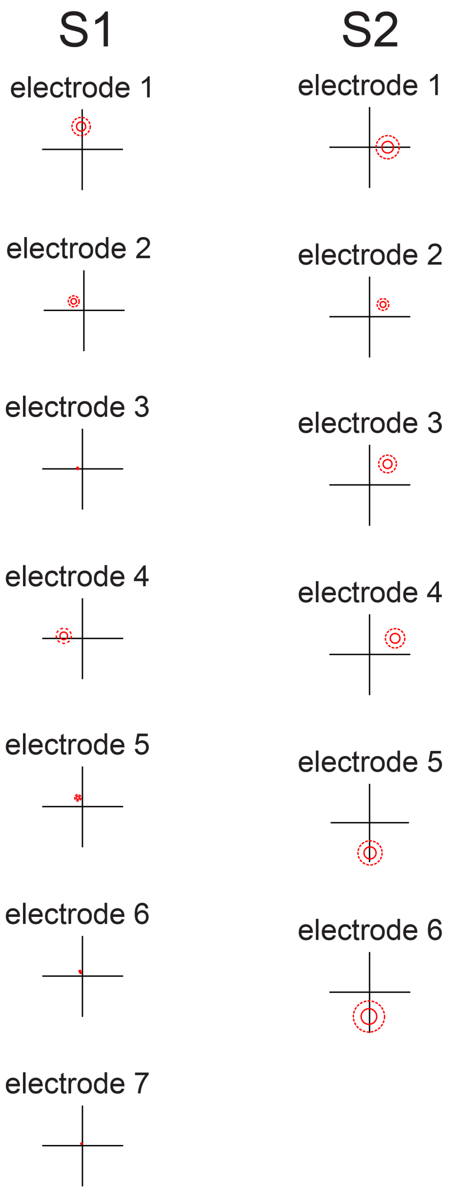

Figure S6: location of population receptive fields (pRF) for S1 and S2. The population receptive field for each electrode was defined by a Gaussian, indicated by the 1- and 2-sd contours (solid and dotted red lines).

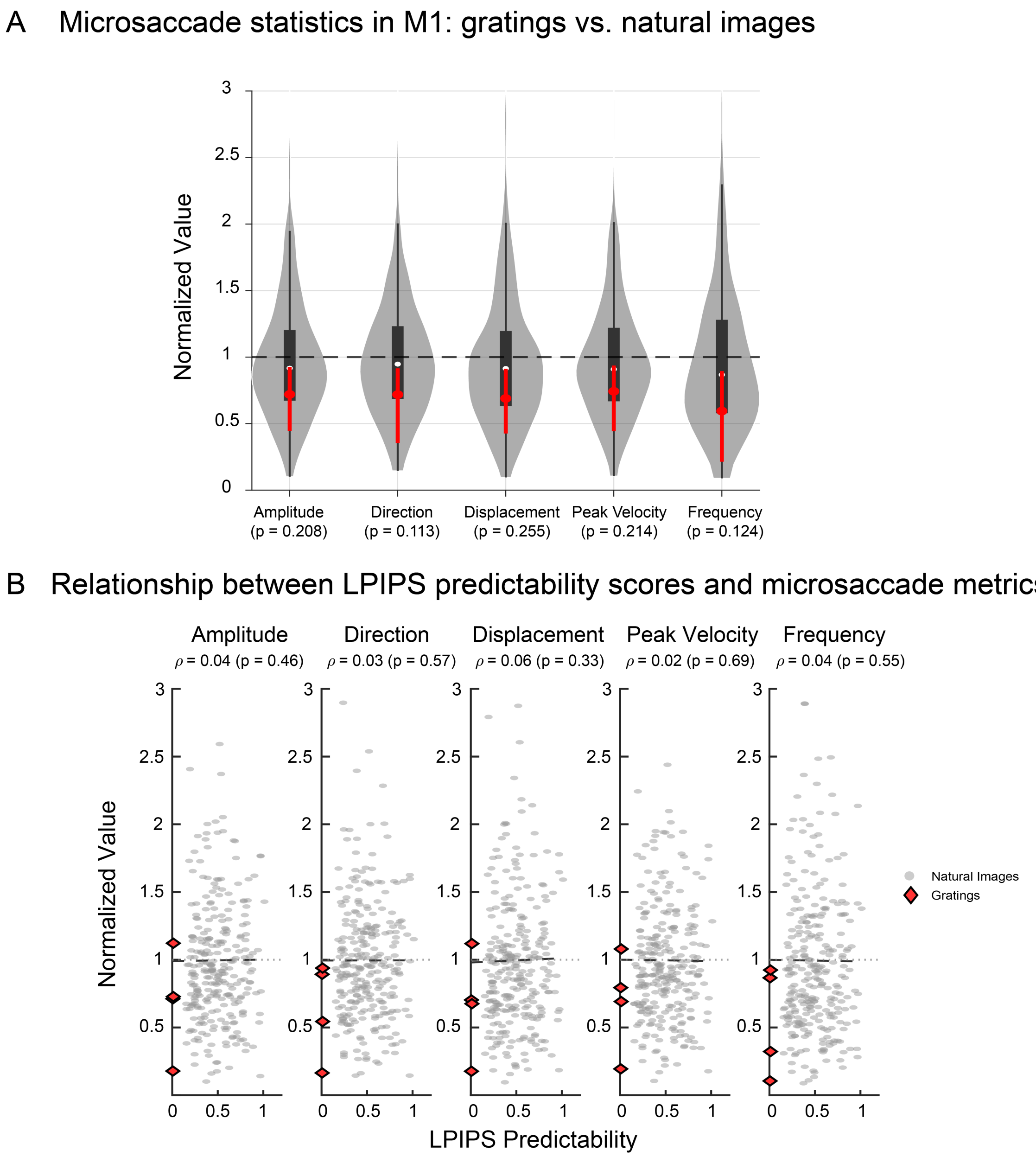

Figure S7: Eye Movement Analysis. To address potential oculomotor confounds, we analyzed eye-tracking data collected during the presentation of both natural images and gratings, which were used in all other analyses. **(A)** Microsaccades were detected using the algorithm as described in Otero et al. (2014). We extracted five kinematic metrics for comparison: amplitude, direction, displacement, peak velocity, and frequency. For each metric, we compared the distributions of the natural images and gratings. For visualization, all metric values were normalized to the mean of the Natural Image condition (Natural images Mean = 1). We assessed statistical significance using the nonparametric Wilcoxon rank-sum test. In the resulting plots, the natural image distribution is shown as a violin plot (gray), while the gratings are summarized by their median (red circle) and interquartile range (red vertical line). **(B)** The relationship between LPIPS predictability scores and microsaccade metrics. Grating stimuli are shown in red, and natural images are shown in gray. Grating stimuli follow the same distribution as natural images; they do not form an outlier cluster that drives a spurious trend. Across the pooled dataset, we found no significant correlation between image statistics and microsaccade dynamics (Spearman correlation, $p > 0.05$ for all metrics).
