# Supplementary material for: Broadband synergy versus oscillatory redundancy in the visual cortex
Source: Nat Commun. 2026 Apr 28;17:5568. doi: 10.1038/s41467-026-72444-2 (PMC13294381; doi:10.1038/s41467-026-72444-2)
Supplement: Supplementary file 3 — Transparent Peer Review file [file 41467_2026_72444_MOESM3_ESM.pdf]

nature portfolio

Peer Review File

Broadband synergy versus oscillatory redundancy in the visual cortex

Corresponding Author: Dr Andrés Canales-Johnson

This file contains all reviewer reports in order by version, followed by all author rebuttals in order by version.

Version 0:

Reviewer comments:

Reviewer #1

(Remarks to the Author)
General:
This paper reports an interesting finding from two human and one Macaque subject performing a visual discrimination task while having ECoG (human) or LFP (Macaque) recorded. After using principal component analysis to separate broadband (BB) from narrow-band-gamma (NBG) oscillatory brain activity, they computed mutual information (MI) and co-information (co-I) in order to measure synergistic and redundant encoding of the information in the visual stimuli. They concluded that the BB activity was mainly encoding information synergistically (i.e., relying on the joint activity of the brain areas) whereas the NBG activity was mainly encoding information redundantly. Moreover, the BB activity preceded the NBG activity, indicating different roles, with BB relating mainly to initial stimulus processing, and NBG relating to maintenance of stimulus information over time.
Although the sample is small, and will require replication for generalization, the demonstration of the result in both human and Macaque individuals is impressive, and lends credence to the identification of the separate roles for BB and NBG neural activity in visual sensory processing. In general, the methods are appropriate (although details are missing, which led to some confusion for me – see specific comments) and rigorous.
Critique: (1) I presume that the BB activity identified is closely related to spike rate and (sparse?) population coding (cf. Rolls & Treves, Prog. Neurobiol., 2011), although this is not made explicit. More could be said about this and its relation to oscillatory activity – perhaps in the context of the work of Akam and Kullman (which is cited). Or some other conceptualization of BB activity could be developed. (2) The statement of results before the methods (as is the convention for this journal) makes this paper somewhat difficult to understand – so perhaps a bit more method detail in the results, especially on how the MI and co-I were calculated would help. This applies especially to the "contrast" between gratings and noise (or gratings and natural stimuli). I have made several specific comments below on this. At the least the relevant equations, exactly as calculated, could be given in the methods. (3) Although the work of Donoghue and Voytek is cited (once for a tangential reason), their approach to separating BB (in their case 1/f^n) from narrow band (not just gamma) activity is not mentioned. I wonder whether the conclusions would be the same using their approach, which involves scraping the narrow band "bump" off of the 1/f-like broadband PSD. I note that the BB activity does not seem to be 1/f-like (Fig. 2A) whereas the NBG does appear to have this character for higher frequencies. At the least it could be mentioned that theirs is a different approach that might be complementary or even highly similar to the PCA approach taken here. (Note, I am not in any way affiliated with that group). (4) The values of MI and co-I found are rather small, in the range of 0.1 to 0.3 bit. It would be good to see a comment about the real significance of these amounts of information. Do they represent poor, fair, good or excellent stimulus discrimination in these brain areas?

Specific:
1. Figures 3, 4, 5: error bars are very faint in MI plots and appear to be missing in some plots, although they may be smaller than the line thickness….

2. Page3: bottom left: "Within the framework of information theory, MI is a statistical measure that quantifies the strength of the dependence (linear or nonlinear) between two random variables. It can also be seen as the effect size, quantified in bits, for a statistical test of independence. Thus, we used MI separately for the BB and NBG responses to quantify the difference between image categories on a common effect size scale." Here is where it would be good to explain just how MI works as an effect size for differential responses to the gratings vs the noise stimuli. It's because the Response-Stimulus MI goes both ways: $I(R;S) = H(S) – H(S|R) = H(R) – H(R|S) = H(S) + H(R) – H(R,S)$ where $H(x)$ is marginal entropy, $H(x|y)$ is conditional

entropy, and H(x,y) is joint entropy. More can be said in the Methods section.
3. Page 11: Methods, Data acquisition: no information is given on the filtering of the ECoG data. This should be given. Also, re Macaque recordings: it seems rather odd to use a filter from 0.35 Hz to 7500 Hz to isolate LFPs. I guess this was the filter for the original data recording. More usual for LFPs is < 250 Hz (cf. Jia & Kohn, 2011). One will still get spikes, and also the LFP >100 Hz is mostly related to spike rate (Ray & Maunsell, 2011, PLoS Biology). This makes me wonder how much of the BB signal is spikes unless some unspecified filtering was also performed. More detail is needed in the description of these data.
4. Page 11: Separating BB from NBG signals: Spectral decomposition: more details on the origin of the PSD covariance matrix (upon which the eigendecomposition was performed) should be given. What is covarying? It should not be necessary to go to the earlier papers to know at least this much about the method.
5. Page 11, Stimuli and task: "These patterns varied across well-controlled image dimensions, including special aperture, orientation, contrast, sparsity, and coherence (Hermes et al., 2019)." Did you mean "spatial" aperture?
6. Page 12, Mutual Information analyses: More detailed information on these analyses, including equations (similar to that in the co-I section), needs to be added here and referred to directly in the main Results section. It is difficult to figure out from the general statements in the results section about MI and Co-I exactly how they were calculated in each analysis/Figure. What was the equation actually used for MI for each of the analyses? Finally, the MI calculated in the GCMI toolbox needs a bit more detailed explanation. The Gaussian cupola method is complex – and quite different from the direct binning or the Kraskov nearest neighbour method - and the Methods section is where at least the major steps could be outlined.

Reviewer #2

(Remarks to the Author)
This work from Roberts et al. investigates the contribution of broadband (BB) and narrowband gamma (NBG) activity to visual processing in the cortex using information theory analysis of ECoG and LFP recordings from human and macaque visual areas. The authors apply spectral principal component analysis (SPCA) to separate BB and NBG components and report that BB carries early synergistic information, while NBG reflects sustained redundancy. These findings are interpreted as evidence for distinct functional roles, with BB supporting nonlinear integration and NBG reflecting linear and redundant information transfer.

The topic is timely and relevant, and the question of how different spectral components contribute to information coding is of general interest. However, I am not fully convinced that the analysis is robust enough to support the authors main conclusions. SPCA has been used previously to separate broadband from narrowband signals but there is no clear conceptual justification in the manuscript for why two spectral PCs should cleanly isolate oscillatory from non-oscillatory activity, and no additional validation of this claim is provided. Several electrodes are also excluded without any quantitative explanation, and no NBG components could be extracted from the V4 macaque data. This raises concerns about the reliability and specificity of the method which is problematic since its the foundation for all subsequent conclusions. As discussed below, I also find the interpretation of the second component as narrowband gamma insufficiently supported by the data.

The dataset is limited to three recordings, with low electrode counts in the human data. Statistical comparisons also lack key details such as the sample sizes for statistical tests and whether tests are done across trials or electrodes. Lastly, some core claims, such as the distinction between diagonal and sustained redundancy, are not convincingly demonstrated in the figures. In fact, the NBG component often shows stronger diagonal structure (even before stimulus onset), which appears inconsistent with the authors' interpretation.

In summary, while the manuscript presents an interesting idea, the methodological limitations and ambiguity of the results currently limit the impact of the work. Substantial extensions and clarifications are needed to strengthen the conclusions and support the proposed dissociation between BB and NBG signals. Below are my main points to help improve the impact of the work.

Major points:
The contrast between stimulus categories is based on a predictability measure derived from a deep neural network. While this is an interesting approach, the categories used (gratings, noise, natural images) differ in several respects beyond spatial homogeneity. It is therefore not clear whether the observed effects truly reflect differences in image homogeneity or other factors. For example, gratings are known to strongly drive gamma-band activity and elicit stereotyped responses across trials and noise is different from complex natural images or textures. The authors should therefore consider more carefully matched stimulus comparisons or at least acknowledge that homogeneity may not be the only relevant factor. Current claims, such as 'The finding that BB and NBG signals encode information about image homogeneity' are, in my view, not supported by the results.

One way to clearly demonstrate that image homogeneity is a driving factor would be to present noise or natural images with different image homogeneity and show that neural responses change, depending on the amount of homogeneity in a given stimulus. The results of the DNN are also not well explained: Why are the reported mean values low if homogeneity is high and what is distribution across stimuli. Using the DNN results to relate them to the neural data and information theory results could be helpful to support the authors claims.

Lastly, the exact type of gratings and noise stimuli should be described more clearly in the text. Why do the redundancy results differ between S1, S2 and M1? Where different stimuli shown in each experiment?

The entire analysis depends on separating BB and NBG signals using SPCA, yet the manuscript provides no quantitative validation that the resulting components indeed correspond to the desired signal types. In fact, SPCA is described with little conceptual detail or critical evaluation that the approach works as intended. For example, it is unclear why the first and second components should always reflect BB and NBG, respectively, or why it unnecessary to consider additional components to capture all relevant oscillatory activity.

There is also no attempt to verify the frequency content of the resulting components by using more conventional spectral analyses. In fact, the claim that the NBG largely reflects gamma activity between 30-80Hz is hard to follow from the traces in Figure 2A. To me, it seems like most power is below 50 Hz and might also be particularly high in the alpha and beta range. Figure 2A also has very sparse x-axis labels, making it difficult to assess the exact frequencies where the NBG peaks are located. If the authors feel strongly that their analysis effectively isolates NBG activity, this should be demonstrated more clearly.

Moreover, several electrodes were excluded from the analysis upon visual inspection, and no NBG activity was extracted in V4 of the macaque data. Given the substantial literature on visually induced gamma activity in macaque extrastriate cortex, this absence is surprising and calls into question whether the method is sensitive enough to reliably detect gamma activity (or oscillatory activity in general). The authors should at least provide a more rigorous validation of the SPCA results and explicitly compare them to alternative approaches (e.g., band-limited filtering or parameterized spectral decomposition) to resolve these issues.

The dataset used in this study is small, consisting of only three recordings in total (two human, one macaque). The number of analyzed electrodes is also low, particularly in the human data. While this might be a limitation of the available data, it raises concerns about the robustness and generalizability of the results. Statistical tests are reported, but whether comparisons are across electrodes, sessions, or trials and basic information such as sample sizes is frequently missing. For example, Fig. 2 shows mutual information from individual electrodes, but it is not clear how these results generalize.

The interpretation of redundancy and synergy matrices is often ambiguous. The manuscript claims that BB signals show a temporally evolving redundancy profile along the diagonal, whereas NBG shows sustained redundancy over time. However, the actual co-information matrices (e.g., Fig. 3B, E or Fig. 5B) appear to show strong diagonal structure in the NBG component as well. If the authors want to support this distinction, they should separately quantify redundancy along and off the diagonal and show how these measures differ between signal types. Moreover, co-information in Fig. 5B is high even before the stimulus onset, which is difficult to reconcile with the idea that the signal reflects stimulus-driven information. Both redundancy and synergy results in Fig. 6 are sustained over time which is different from human results in Fig. 4, yet the authors describe them as consistent across species and different areas. This difference should be discussed and better explained. The statement in the discussion 'Notably, this broadband activity carried information especially early after stimulus onset, while oscillatory activity carried information substantially later' currently seems to go against the results shown in Fig. 6.

The discussion presents a range of interesting ideas about the role of BB and NBG signals in nonlinear integration, resonance, and inter-areal communication. However, many of these claims are speculative and not clearly linked to the data. For example, the idea that BB reflects nonlinear recurrent dynamics and NBG reflects linear information transfer is not demonstrated directly. The interpretation of NBG as reflecting gamma oscillations is also questionable given the unclear spectral content. Overall, I think a more restrained interpretation of the findings would be appropriate unless stronger evidence can be provided to support these functional distinctions.

Minor points:
Figure 2A lacks sufficient axis labeling to judge the frequency content of the BB and NBG components.

Figure references in the text are repeatedly mismatched (e.g., references to Fig. 1D seem to describe Fig. 1C. I dont think Fig. 1D is actually explained in the text). This should be corrected to improve clarity.

It would be helpful to show the raw or filtered traces to Fig.2 to better illustrate how BB and NBG relate to the original data.

Please add individual data points for all electrodes to the box plots ,e.g. Fig. 3C.

The statement "While the BB response is induced by all images, including spatially heterogeneous images, the NBG response is predominantly induced by spatially homogeneous images such as gratings." does not seem match any of the results. Is this shown somewhere or only refers to other work?

It is not clear to me why the co-Information in Fig. 6 is so asymmetric. Why is there such strong difference when computing co-I between V1-V4 or vice versa? It would also be good to show statistical results for all electrodes (similar to earlier figures) to provide an overview if results are consistent.

Reviewer #3

(Remarks to the Author)
The article by Roberts et al. examines how broadband signals and narrowband gamma oscillations contribute to the

encoding of visual information. The authors analyzed electrocorticography (ECoG) recordings from two human subjects and local field potentials (LFPs) from one monkey and used information-theoretic approaches to examine whether signals convey redundant (shared) or synergistic (complementary) visual information related to image statistics namely, the spatial homogeneity of stimuli. Their analyses revealed that broadband signals encode synergistic information, whereas narrowband gamma oscillations primarily carry redundant information. Based on this the authors suggest that broadband dynamics subserve nonlinear mechanisms that serve pattern recognition, whereas narrow band oscillatory activity facilitates information sharing that can enhance robustness of encoded information.
The paper is mostly well-written and puts forward an interesting proposal that can advance our understanding of neural coding. However, some questions pertaining to the generalization of the findings for visual information processing remain open. Additionally, there are several points where clarity could be improved to help the reader navigate through the analyses, results and their interpretation and appreciate their potential importance. We list below some suggestions toward this end.

Major comments:
1. This may sound overly simplistic but the very idea of finding that a broadband (BB) signal which by definition holds a rich frequency content carries synergistic information does not sound extremely surprising. It is possible that I am missing something but it would help if the authors could interpret their results (and perhaps frame their hypothesis) taking into account how different the two signals (BB and narrowband) are and what kind of biases or limitations this difference imposes.
2. The authors use data from two human subjects and one monkey. Results are variable between the two human subjects as well as the monkey data in certain cases. For example, the data shown in Figure 2B for BB response for gratings and noise/natural images do not allow a solid conclusion for the role of BB signals in carrying information about homogeneous images. In S1 the response is earlier for gratings and the one for noise follows, in S2 there is a higher initial response for noise and in the monkey there is a higher response for gratings. This seems to be a representative electrode in V1 which raises the question of what other electrodes look like. The authors do not comment on these. In S2, one might even detect a later sustained higher response for gratings in BB (something that is typical for NBG signals). A more nuanced discussion of these would help.
3. Previous work by the authors in human visual cortex (e.g., Hermes et al., 2015a) and macaque V1 (e.g., Uran et al., 2022) has shown that gamma oscillations are induced by homogeneous, predictable stimuli (e.g., gratings), whereas unpredictable stimuli (e.g., complex natural scenes) evoke weak or no gamma oscillations. How do these previous results relate to the current findings presented in Figure 2B, C? Differences between gratings and noise, or between gratings and natural images, do not appear to emerge at the initial onset of narrowband gamma, but rather during the sustained phase. These effects appear less pronounced than the narrowband power spectra shown in Figure 1 of Hermes et al. (2015a). This raises questions about the validity of the decoupling method. Is it possible that the method used to extract narrowband gamma in the current study takes into account a broader frequency range (30–80 Hz), whereas the actual effects may be confined to a narrower frequency band? At the very least, it would be useful to discuss how and why the current results may differ from those in previous studies.
4. In Figure 2C, the authors report that the onset of mutual information differs for BB and NBG signals, however, no statistical comparison of relative latencies is carried out. This should be provided in order to support the relevant claim.
5. The authors show synergistic information for BB signals that appears to be transient in most cases. It is hard to conceive what this would reflect in terms of information processing and the relevant dynamics. It would be helpful to comment on that.
6. When examining between areas interactions, the authors mention pairs of areas in which redundant or synergistic information was found. It would be extremely helpful to better appreciate this finding to provide a supplementary figure with examples of pairs of areas where no significant interactions were found. Although electrodes in human subjects are reported to cover several different areas it is not clear what the results looked like for pairs other than V1-V2/V3.
7. Previous studies have reported that interareal functional connectivity (e.g. spike-LFP coherence) is stronger for sites with overlapping RFs. Did the authors examine whether and how RF location of different sites influences co-information? This is critical to understand the potential role of distinct neuronal dynamics in a distributed network. If the sites sampled did not have overlapping RFs, this should be discussed and mentioned as a potential limitation.
8. The study did not find narrowband gamma in macaque V4, although gamma power in V4 has been found in previous studies even if this is weaker than in V1. This difference should be stated and some speculation on that should be provided to avoid confusion. Moreover, the fact that NBG was not found in V4 did not allow the authors to confirm the finding that inter-areal interactions as reflected on NBG activity carry redundant information. This should be acknowledged.

Minor comments:
Computing a measure of image homogeneity:
1. Abbreviations S1 and S2 for human subjects 1 and 2, and M1 for the macaque are used before being defined in the main text.
2. “gratings had the highest image homogeneity () and noise () patterns had the lowest image homogeneity”. Maybe move the word patterns before the parenthesis.
3. Including an explanation in the Methods section of what the degrees of freedom in the t-tests correspond to would improve clarity.
Decoupling broadband and oscillatory responses from the ECoG and LFP signals
4. Please rephrase: “In each electrodes and datasets”.
5. When discussing the decoupling of signals, the authors refer to Figure 1D, when they should be referring to Figure 1C.
6. Figure 2A shows some noticeable noise peaks for S1. Would notch filtering these peaks affect the results?

Temporal redundancy in BB and NBG signals

7. A brief overview of co-information as an extension of mutual information to more than two variables in the main text would help the reader follow the subsequent analyses more easily.
8. Are the diagonal vs. square patterns observed here related to those found in cross-temporal decoding analyses (e.g., Meyers et al., 2008)? Making this connection would help readers who are more familiar with decoding approaches better follow the results.
9. There are several figure-related issues: (a) Top panels of Figure 3 display green pixels that are not indicated in the color bar; (b) the error bar for V1 MI in the top panel of Figure 4D appears misaligned; (c) Figure 6 probably lacks an error bar for V1 MI and (d) the reference to Figure 3A,C for diagonal redundancy in BB signals of S1 and S2 should probably be Figure 3A,D.
10. In Figures 3C and 3F, does the y-axis represent 0.4% or 40% of significant time points? If it is 40%, please correct the axis or label accordingly. Same applies to Figures 4 and 5.
Spatiotemporal redundancy in BB and NBG signals
The reference to specific panels of Figure 4 in the second paragraph should be corrected.
Spatiotemporal synergy in BB and NBG signals
11. The text currently reads: “In contrast, and consistent with the findings within regions, interareal synergistic information was mainly observed in the BB signals in S1 and S2 (Figure 4A,C)”. Should the reference be to panels A and B instead of A and C? This is repeated in the following sentences.

Methods
Macaque recordings
12. It should be justified why previous related studies by some of the authors (e.g., Uran et al., 2022) used data from three monkeys whereas the current study reports data from only one animal. If the third animal in the Uran et al., 2022 study is the same as the one included here, it appears that it was implanted with Utah arrays. This needs to be stated.
13. The text reads “Monkeys self-initiated trials”. It should be “monkey” instead.
Stimuli and task
14. The acronym LPIPS is not defined.
Separating BB from NBG signals
The text reads: “3 electrodes (S1: V3 ventral; S2: V2 ventral, V2 ventral”. The closing parenthesis is missing and “V2 ventral” is repeated.

Reviewer #4

(Remarks to the Author)
I co-reviewed this manuscript with one of the reviewers who provided the listed reports. This is part of the Nature Communications initiative to facilitate training in peer review and to provide appropriate recognition for Early Career Researchers who co-review manuscripts.

Reviewer #5

(Remarks to the Author)
This manuscript presents an intriguing and conceptually rich investigation into the distinct roles of narrowband gamma (NBG) oscillations and broadband (BB) signals in encoding and transmitting visual information. The authors leverage information-theoretic measures—redundancy and synergy—to probe how these signals differentially contribute to visual processing, with the hypothesis that NBG reflects feedforward sensory input and network coordination, while BB may index nonlinear dynamics supporting recurrent, modulatory feedback. Using ECoG and LFP recordings from nonhuman primates viewing homogenous (gratings) and heterogeneous (naturalistic or noise) images, the study applies spectral PCA to dissociate NBG and BB signals and uses DNN-derived predictability scores to quantify image homogeneity. This combination of signal decomposition and information-theoretic analysis provides a promising framework for disentangling the functional contributions of different neural signals. That said, there are some inconsistencies in the exposition and rationale that reduce clarity. In several cases, key concepts, figure captions, or methodological choices required inference on the part of the reader. I also have concerns about the limitations of the methods that are not sufficiently addressed. With clearer justification and validation for some of the analytical decisions, the manuscript would be substantially strengthened.

1. The report contains no assurances of ethical oversight or approval for research with human or animal subjects.

2. The use of spatial homogeneity as a stimulus dimension for manipulation is not motivated in the report. Why is this important, interesting, ethological, etc.?

3. The use of the term “homogeneity” by the authors seems to conflict with my intuitive understanding of the term. For example, generally pure “noise” I think would be considered more homogenous than a grating. Sure, it’s harder to predict the exact pixel values given the surround, but the statistical *texture* of the image is more spatially uniform.

4. It is not apparent to me why a DNN should be expected to complete the center of an image in a way that is texturally homogenous with its surround. For example, if given a picture of a sunflower in which the central disk has been masked

leaving only the surrounding ray petals, my impression is that the DNN should not fill in the center with more yellow petals, but rather complete the image as a sunflower with a dark central disk. But this image would not be spatially homogenous.

5. Was the image model trained on images of gratings and noise patterns? The relative image completion performance for different classes of images might be explained not by differences intrinsic to the test images, but rather to the training history of the DNN. Can the authors rule this in/out? Rather than actually tapping into image homogeneity, might the authors just be tapping into how familiar the test images are to the DNN, for reasons not due to their image statistics but rather due only to the decisions of the people that trained the model?

6. The authors report "As expected, gratings had the highest image homogeneity (S1: xbar = 0.171…)...". What is x-bar? What are the units? Can any context be given for these numbers? It looks like maybe lower numbers imply greater homogeneity? Are these values bounded in some way? These numbers are completely uninterpretable as reported. Actually, now I'm really confused, because for the human subjects the authors report "x-bar" values around 0.17 for gratings, and claim that those images have greater homogeneity than noise, which had "x-bar" values of around 0.3 (implying an inverse relationship between x-bar and homogeneity); however, for the monkey subject, the authors report "predictability" (which I think they're using interchangeably with "homogeneity", but it would be better to just pick one term and stick with it consistently) of x-bar around 0.24 for gratings and say that implies greater homogeneity than natural images with an x-bar of 0.009, which implies a direct relationship between x-bar and homogeneity. So, I really cannot make heads nor tails of this.

7. It is not clear to me why it should be the case, mathematically, that the first SPC always captures the broadband activity and the second SPC always captures the narrow band gamma activity. I could believe this could be the case in practice, but not mathematically guaranteed. What's to prevent the NBG from being captured by the third SPC instead in some cases? It strikes me that rather than simply taking the components with the top two eigenvalues, it would make more sense to scrutinize the properties of the components to see which, if any, have the expected properties of broadband or NBG activity.

8. On that note, since the objective is to separate components that are assumed to be superimposed, would not independent components analysis (or factor analysis), rather than principal components analysis, be the more appropriate method for the stated objectives? Principal component analysis does not include source separation as an explicit objective (i.e., mixtures of sources can be captured in a single PC if the covariance is large enough), whereas ICA or FA do so.

9. How did the authors determine whether NBG was "observed" in a component? Was this done just by visual inspection, or was some objective, quantitative testing done? Ideally this should be objective. What was the authors' criterion for considering a gamma band to be "narrow"? For example, in figure 2a the bandwidth of the NBG curves is, in the opinion of this reviewer, quite wide (especially for the human subjects: for S2 the magnitude of the signal is roughly the same from 12–60 Hz (please add finer graduations to the abscissas on these plots, which would greatly ease our ability to interpret these).

10. It is not clear if the authors calculate MI at the single-channel level, or if this is multivariate.

11. On p.3 when the authors describe "[quantifying] the difference between image categories", it is not clear if they mean e.g. gratings vs. noise, or the various categories of natural images, or high or low homogeneity, or anything else. It should be clearer what are the categories being compared.

12. In the results (p. 3) when the authors report "We observed significant information encoded in…" not enough information is given about the nature of the statistical test here, in the figure legend, or in the methods. The results text doesn't even state what the type of test was (permutation test we eventually learn), but we should also know which variables were shuffled and along which dimensions, the number of iterations that were performed, etc.

13. There seems to be a bit of inconsistency between the result description and the figure captions for their MI data (figure 2). In page 3: "We observed significant information encoded in the BB (dark green) and NBG (light green) ..."; but then in the figure caption (fig. 2c): "between gratings (dark green) and noise images (light green)". The legend inset in the plots also says dark green = BB (not dark green = gratings, as implied in the figure legend). Is the figure legend incorrect? It's hard to understand how MI would be computed *within* a stimulus category, since I think the MI is about image category itself.

14. Throughout the manuscript, the authors report approximate values where it seems they should have made precise measurements under quantifiable uncertainty. For example, "This was observed in S1 (BB: ~40-150 ms)..." (p. 3). It would be more professional to report the actual measures of central tendency and the dispersion, such as "...S1 (BB: 39.6(0.2) ms [mean(SD)] to 150.2(0.6) ms…"

15. P.4: “Redundancy quantifies the degree to which two time points carry the same trial-by-trial information about the stimulus contrast.” What do the authors mean by “contrast” here? Luminance contrast? Or a specific type of comparison between conditions maybe? Perhaps the authors intended “homogeneity” rather than “contrast”?

16. How do the authors determine whether a pattern of redundancy/synergy is “diagonal” or “blocky”? Is this by visual inspection? Or is there an objective, quantitative test? For this claim, the test should be objective.

17. P.4: “We observed more temporal redundancy in NBG…” this phrasing is ambiguous as to whether “more redundancy” means greater intensity, longer duration, some combination thereof, or something else. If I’m piecing this together correctly, what the authors mean here is that they found a greater cardinality of significant time points.

18. In figure 2, what is “Magnitude”? Given the methods –which describes computing the PSD and then performing PCA –I was expecting something in volts (or perhaps volts squared, or volts^2/Hz). There is no method described for normalizing or scaling the SPCs into something called “magnitude”.

19. When computing the PSDs for the SPCA procedure, isn’t the phase information lost? I don’t understand how an inverse transformation into the time domain could be done properly if the phase information is discarded (should result in complex values in the time domain). I would appreciate more clarity on this method.

20. The data are initially and exclusively presented at a high level of processed abstraction. Inclusion of less processed results (e.g., spectrograms of example channels) would improve the report and make readers better able to interpret the quality of the data.

21. Can the authors rule out the influence of microsaccades? One could imagine that microsaccades when viewing a spatially heterogeneous image would lead to a greater change in the retinal input than microsaccade when viewing a spatially homogenous image. Here I mean homogeneity in my intuitive understanding in that a white noise pattern has a more homogenous texture than a grating. The predicted pattern of results under such an interpretation would look exactly like the NBG plots in figure 2B.

22. The vertical axis in figure 3c is labeled as “%”, but the maximum value on that axis is less than 0.5, i.e. less than half of one percent, which would be quite a small value for this. Do the authors perhaps mean to label this rather as a *proportion* of significant time points, rather than percent? This comment applies to like figures.

23. P.5: “This off-diagonal synergy has a characteristic pattern…” How did the authors determine that this pattern is characteristic of temporal synergy within BB signals. Is there an objective, quantitative test for this, or is this a qualitative opinion of the authors? Later in the same sentence: “...aligned with each temporal axis…” Again, is this handwaving, or is there some quantitative testing behind this claim? For this claim its probably acceptable to use qualitative interpretation by the authors, but it should be clear what the basis for the claim is.

24. Figure 2 (and others): it is not reported which measure of dispersion is represented by the shading.

25. P.7: “Similarly to the results observed within areas, interareal interactions…” What evidence do the authors have for interaction between the brain areas here? If I’m following correctly, this same analysis could be applied, e.g., to two populations recorded in different monkeys viewing the same stimulus set on different days in different laboratories. The analysis tests whether the information in two populations is redundant or synergistic, but does not depend on interaction between the populations, as far as I can tell.

26. Figure 6, the ordinate tick marks are unlabeled.

27. First sentence of Methods says the recordings in monkey were ECoG, whereas the Results says the recordings in monkey were LFP. Please clarify. Note also that Hermes et al., 2019 does not seem to report monkey (Methods says “data acquisition details are reported in full in [that paper]”).

28. Hermes et al., 2019 –to which we are directed to learn the full methodological details –describes three human subjects, not two. Was one subject excluded from this, and why?

29. The Methods reports the monkey subject as “Monkey H” whereas the rest of the report uses a different identifier (“M1”). However, in order to understand the methods, which are very brief in this report, the authors point to Peter et al., 2019, which refers to one subject as “Monkey H”, which I’m assuming is this same monkey. So, the authors should probably use “Monkey H” throughout the report to refer to this subject.

30. Methods: “Trials were aborted during which the eye position deviated…” How was this determined? There is no reporting of gaze tracking methods in either this manuscript or in Hermes et al., 2019. Edit: it looks like eye tracking is reported in Peter et al., 2019. I’ve been really thrown off by the first sentence of the Methods that includes “monkey” and then points to Hermes et al., 2019 for “details… reported in full.” Better organization and clarity would help a lot, and the authors should include enough information in this manuscript itself to understand the key details (such as that the monkey’s gaze was tracked with an optical eye-tracker, that it had a Utah array implanted, etc.)

31. Methods: “Human subjects were repeatedly presented with each type of stimulus image (15 trials per image) for a period of 500 ms…” What is the order of presentation? Are the trials randomly interlaced? Is it blocked?

32. “...presented… at an approximate visual angle of 20 [degrees]...” Is this an angle of eccentricity or an angle of distention? In either case, the other value should also be reported. I.e., if the stimulus is 20 degrees in distention, where in the visual field was it presented? If the stimulus was presented at 20 degrees eccentricity, then how big was it?

33. What was the nature of the stimulus display (I’m guessing a computer monitor, but it isn’t specified)? What was displayed between stimuli? Was there any kind of fixation point? These details are also not found in Hermes et al. 2019

34. Methods describe stimuli with curved lines, but the rest of the report only mention gratings, noise, and natural images. Please clarify.

35. P.11: “...including special aperture…” I do not understand what “special” means here.

36. On p. 11, beginning from where it says “Unlike the human subjects, Monkey-H was presented with…” to the end of that paragraph is extremely hard to understand. The monkey only ever saw maximum of one grating in a session (possibly repeated 20 times), is that right? The whole thing about the “mode least predictable natural stimuli” and “thus selecting 80 trials” has me completely flummoxed. I cannot understand what happened here, and why.
37. On page 11 there’s a part that starts, “In brief, this network models the visual system as an OR-CNN,...” and then the paragraph abruptly ends mid-sentence, then there’s a one-sentence paragraph that doesn’t seem to follow, and then yet another new paragraph that also seems a bit disconnected. Something seems to be missing around here.

38. Methods say “Each image was masked 26 pixels from its center in width and height”. When I first read that I was confused as to whether 26 pixels was analogous to radius or diameter. However, I become more confused when later in that paragraph a 104 x 104 pixel mask was described, which wouldn’t be consistent with 26 pixels as *either* a radius or diameter.

39. In the methods, when the authors write “We ranked the image categories by their predictability…” what are the categories being ranked, exactly? Just the two categories of “gratings” and “noise” (or “gratings” and “natural images”)? What is the point of this ranking step?

40. The claim that Spectral Principal Components “[represent] distinct neural processing components” seems to reach beyond its grasp. What is a “neural processing component”? What would make such components distinct?

41. The authors perform SPCA, select components, and then perform the inverse transform back into the time domain. How is phase information used for this? Is this inverse transformation a “causal” operation, or could late time points come to influence earlier time points (acausality) through this process? More generally, to what extend does this transform/manipulate/inverse transform method have the potential to *introduce* information redundancy (or synergy) in the time domain? Have the authors tried this on simulated data reflecting the null hypothesis, or for which the ground truth of the underlying encoding mechanisms is known?

42. In order to compute MI, the probability of observing a particular neural datum needs to be known/estimated. Because the voltage values are continuously valued, it is highly unlikely that the exact same voltage appears more than once in the data,

which prohibits estimating the probabilities from the neural data sample itself. To deal with this issue, people usually discretize their neural data observations (such as by computing a histogram and then using the number of observations in each bin to estimate probabilities). In order to interpret and replicate this analysis, we need to know how the neural data were discretized. This information is not reported.

43. Does the GCMI toolbox have any user-configurable options? If so, these should be reported.

44. Methods: "... the number of electrode pairs analyzed for the between-electrode co-I analyses were 12 for BB and 4 for NBG signal pairs for S1 (Figure 5), and 8 BB and 6 NBG signal pairs for S2 (Figure 6)." Figures 5 and 6 show monkey data, however, not human data.

Version 1:

Reviewer comments:

Reviewer #1

(Remarks to the Author)
I think the authors have done a good job in responding to the reviewers' comments and suggestions. I accept the revisions in response to my own comments. Regarding those of the other reviewers I can only say they seem thorough to me, including several instances of clarifying misunderstandings, but of course I cannot speak for them. I did notice one typo in the revised ms: line 94 NGB instead of NBG - this mistake occurred in several places in the responses to reviewers so I guess it was also missed in this case in the ms.

Reviewer #2

(Remarks to the Author)
I appreciate the authors' effort to clarify their spectral decoupling approach for separating broadband (BB) from narrowband oscillations and more explicitly describe how narrow-band gamma oscillations (NBGs) were identified. The additional text in the results and methods sections substantially improves the readability of the analysis and strengthens the overall quality of the manuscript. The new supplementary figures are also helpful, in particular Fig. S1 for demonstrating the separation of BB and NBG in SPC1 and 2.

Overall, I think that the manuscript has much improved and it is now easier to follow the general logic and main conclusion. Below are some remaining points that I believe would further strengthen the paper:

- When using the OR-CNN to compute homogeneity, I wonder what extent predictability depends on the training data used to construct the model. Specifically, to what degree might the model's performance reflect learned priors about natural image structure (e.g., inferring missing parts such as wheels on a car)? More generally, how sensitive is the LPIPS-based homogeneity measure to the specific training regime of the network?

- Despite the additional descriptions, it is still unclear why the narrowband component in SPC2 is specifically referred to as a gamma oscillation. Although NBGs were identified with a threshold in the range from 30-80 Hz, the example SPCs in Figure 2 and S1 appear to have significant contributions from lower frequencies. For instance, the NBG for S2 in Fig. 2a and Fig. S1 appears to peak around ~25 Hz and falls off at higher frequencies, while the NBG for S1 rises near ~10 Hz and remains elevated only up to ~50 Hz. Specifically referring to these components as gamma oscillations therefore appears to be a potentially misleading. If the authors feel strongly that NBG is the right label to use to align with the literature I would suggest to at least change lines 128-130 which explicitly states that SPCA2 peaks in the gamma range which seems to be at odds with these results, especially the S2 spectra.

- I appreciate the inclusion of the two summary figures in S3B and S4B to show that results from individual electrodes appear to be consistent across electrodes within the same object. To further improve clarity, it would be good to provide more information in the results on which statistical test was done on which data and also name the sample size. For example, for the result in lines 267-271 I assume from the text in the methods that a two-sided t-test was used to compare BB to NGB across n = 7 versus 4 across electrodes. Throughout the manuscript, it would be easier to understand if a test was done across trials, electrodes or electrode pairs by directly adding the n (e.g. n = 4 electrodes or 80 trials, 80 images etc) in the text.

Reviewer #3

(Remarks to the Author)
The authors have addressed in a satisfactory manner all of my concerns and suggestions.

Reviewer #4

(Remarks to the Author)

I co-reviewed this manuscript with one of the reviewers who provided the listed reports. This is part of the Nature Communications initiative to facilitate training in peer review and to provide appropriate recognition for Early Career Researchers who co-review manuscripts.

Reviewer #5

(Remarks to the Author)

I have a mixed reaction to this revision. Some of the more straightforward remedies have been tackled, but the more fundamental issues have not been satisfyingly addressed. The methods reporting is greatly improved. However, the greater clarity has exacerbated, rather than assuaged, my concerns about how the authors are constructing isolated-component time series in their analysis. I appreciate the inclusion of some less-processed, descriptive analyses of the data. I continue to disagree with the use of "homogeneity" here, and find that that terminology confuses, rather than clarifies. I continue to think that the use of homogeneity as an independent variable has not been adequately motivated. Some of the responses from the authors do not seem to engage with the concerns substantively (e.g., on the training history of the DNN and the potential impacts, or on the impacts of microsaccades). The response about microsaccades was especially disappointing, considering that the authors reported being in possession of eyetracking data that could speak to this rigorously.

I'm still not on board with the authors' use of the term "homogeneity", which simply seems incorrect. They have not persuaded me otherwise with the revision. The methods refers to a "predictability score"... why not just use this, more intuitive label for the metric of interest? It seems unnecessarily confusing to re-label this measure as something unintuitive.

The authors seem to have made no attempt to address my question about the behavioral relevance / ethological validity of their "homogeneity" measure. Why is this interesting/important? Why "homogeneity" as opposed to any other stimulus property? The authors' guiding thesis seems to be that BB is more internal/inferrential sort of processing whereas NBG is more external/stimulus processing, so shouldn't the same analysis work for practically any stimulus feature you might want to try this with? Why or why not? The authors' motivation for "homogeneity" seems to boil down to (paraphrasing): "we looked at this because we thought it would be correlated with at least one of our dependent measures given the literature", which is not satisfying. They also say that they're not interested in homogeneity itself, but are instead interested in NBG and BB activity. However, why should the readers be interested in those phenomena either? If the only motivation is to link them to another phenomenon that we also haven't been given any reason to care about, then that is not effective.

I am not at all moved by the authors' response about accounting for the training history of the model (i.e., that the model was trained on natural images but not the other stimulus classes, and how might this impact things). They seem to have made no real attempt to address this. Another reviewer (at least one) also raised this issue.

In their rebuttal the authors state that they do not inverse transform back to the time domain after their SPCA procedure. However, the legend to Figure 1 clearly states: "BB signals are computed by reconstructing the time series of each stimulus category trial with the first spectral principal component (SPCA) of the ECoG/LFP signal", which implies that the time-domain signal of the BB component is reconstructed. If this is not, in fact, what was done, the authors should revise this (and similar) statement(s) to not be so misleading. Actually, now that I've had another pass of the revision after originally writing this point, I see that the authors took their SPC loadings and multiplied those by the original time-domain data to reconstruct BB time series alone (for example). This raises a number of new issues that I deal with separately below.

The legend for Figure 2 still doesn't match what is shown. The legend states "...first (dark green) and second (light green) spectral principal component (SPCA) is shown between 1 and 200 Hz." But the inset says "BB" and "NBG" not "first" and "second". Arbitrarily associating the ordinality of the component with assumed spectral content was a main concern shared among the reviewers. The authors changed from using ordinality to some operational definition, so the legend should reflect that change.

On the point of time-domain signal reconstruction, the authors have revised to write: "For each component, we reconstructed the corresponding time-domain signal by weighting and summing the original ECoG time series according to the component's loading vector, thereby obtaining separate BB and NBG time courses for subsequent analyses." This seems improper, as described. After the PSD step, polarity information at the electrode would be lost if the authors are just taking the magnitudes (unless the "loadings" used by the authors are complex-valued, which they wouldn't seem to be –this still has not been cleared up in the revision). I don't understand how this could work. The math is well-formed, but the result would in no way reflect a reconstruction of the broad-band activity alone. Can the authors show their analysis on some realistic simulated data where the ground truth is known and demonstrate that this does, in fact, reconstruct the correct broadband time series? Also, the fact that I still can't tell whether the PSD is complex-valued in this case or not is another concern. I would have a lot easier time of this if the authors simply reported the sequence of equations that they used, and explained what each variable was, its dimensionality and units etc. E.g., " 'A' is a nTrials x nFrequencies matrix of complex-valued Fourier coefficients in microvolts…." Finally, tying into my point from the previous round of reviews about how ICA would be a more appropriate approach for this study (which the authors dismissed in their rebuttal), because the spirit of the analysis as described by the authors assumes independence that PCA does not guarantee.

The authors rebuttal about microsaccades is not persuasive. They write:
"Firstly, microsaccade-related artifacts affect both gamma-band and broadband dynamics both for EEG/MEG (Schwartzman et al., 2011; Kupers et al., 2018) and intracranial recordings (Lower [sic] et al., 2016), making a selective influence on only

one of the components improbable. …"
First, how improbable is "improbable"? This comment is not rigorous. Also, are both NBG and BB affected in precisely the same way? All we need for a confound is a differential effect. Also, did those previous studies cited by the authors use the same/similar stimuli as the current report? Because the interaction with stimulus processing is a central component of the study, and so comparison to those precedents needs to account for the stimulus differences. The authors rebuttal continues:
"....Secondly, a single ocular artifact provides no precise mechanism for producing opposing information-theoretic signatures; redundancy in gamma and synergy in broadband."
Again, we don't need microsaccades to simultaneously have opposing effects on two phenomena, they only need to associate with one of the phenomena to produce a confound. Moreover, this claim that a singular artifact cannot provide opposing effects on the two spectral components is given simply as conjecture without support, and so is not persuasive.
".... We further note that, firing rates are generally low for grating stimuli (e.g. see Zhou, 2008; Gieselmann &Thiele 2008), which runs counter to the explanation of the reviewer. We also emphasize that the "noise" stimuli here are not white noise, but are band-pass filtered for S1 and S2, and for M1, are natural images." My comment made no mention of firing rate, and firing rate and precise stimulus class are not substantive dimensions of the concern. If there is any spatial variation in stimulus properties across the retina, and anything about microsaccades differs between stimulus conditions (frequency, amplitude, direction, velocity, etc) then this is a potential confound, simply put. Any persuasive response to the microsaccade question MUST include some analysis of eye movement data from the experiment. The authors reported they possess those data. I would struggle to accept a version of this manuscript without such an analysis. Even then, it doesn't guarantee that the microsaccades aren't an issue, but it's an essential starting point.

In one of their rebuttals the authors write "...as SPCA operates purely in the frequency domain…" This conflicts with the description for reconstructing the time-domain signal of the broadband activity using the SPCA loadings. Granted, the SPCA results are just frequency-domain, but then the authors did this time-domain reconstruction. I understand that constructing the time series of the component scores doesn't require phase information, but I'm not concerned about the time series of component scores. Throughout these concerns about time-domain reconstruction I have been specifically concerned with the step now described on line 781 of the revised manuscript. That step does not seem proper as described (albeit mathematically well-formed), and the rebuttal does not address this. Also, in reading line 117 ("The time series for each trial was then reconstructed by using the the weights from the first and second SPC, separately…"), the typical way to "reconstruct" a time series from some PC result is to take some set of component scores and multiply those times the loadings to get back into the original domain. In the absence of more clarification, this would be the consensus assumption as to what was done. However, the authors are not doing that. Rather, they are multiplying the SPC loadings by the original time-series data, which, even if it wasn't highly dubious, is at least highly atypical, and thus behooves a bit more detail as to precisely what was done.

The revised results section continues to talk about the "first" and "second" SP components as if those ordinal labels were meaningful, but the authors stated that in the revision they switch to a functional method for identifying BB vs. NBG components, so this way of presenting the results no longer makes sense.

In the legend to Figure S2 the authors describe a "fre covariance matrix". What is "fre"? Later in the same legend a "compo frequency profile" is mentioned. What is "compo"?

Version 2:

Reviewer comments:

Reviewer #2

(Remarks to the Author)
The authors have adequately addressed my remaining comments. I have no further comments or major concerns regarding the manuscript in its current form.

Reviewer #4

(Remarks to the Author)
I co-reviewed this manuscript with one of the reviewers who provided the listed reports. This is part of the Nature Communications initiative to facilitate training in peer review and to provide appropriate recognition for Early Career Researchers who co-review manuscripts.

Reviewer #5

(Remarks to the Author)
I'm satisfied with the revision. Thanks for the effort. Btw, it seems like part of a sentence is missing around line 833.

Open Access This Peer Review File is licensed under a Creative Commons Attribution 4.0 International License, which permits use, sharing, adaptation, distribution and reproduction in any medium or format, as long as you give appropriate credit to the original author(s) and the source, provide a link to the Creative Commons license, and indicate if changes were made.
In cases where reviewers are anonymous, credit should be given to 'Anonymous Referee' and the source.
The images or other third party material in this Peer Review File are included in the article's Creative Commons license, unless indicated otherwise in a credit line to the material. If material is not included in the article's Creative Commons license and your intended use is not permitted by statutory regulation or exceeds the permitted use, you will need to obtain permission directly from the copyright holder.
To view a copy of this license, visit https://creativecommons.org/licenses/by/4.0/

Please note that we have numbered the reviewers' comments when they were not already numbered for clarity.

REVIEWER COMMENTS

Reviewer #1 (Remarks to the Author):

General:
This paper reports an interesting finding from two human and one Macaque subject performing a visual discrimination task while having ECoG (human) or LFP (Macaque) recorded. After using principal component analysis to separate broadband (BB) from narrow-band-gamma (NBG) oscillatory brain activity, they computed mutual information (MI) and co-information (co-I) in order to measure synergistic and redundant encoding of the information in the visual stimuli. They concluded that the BB activity was mainly encoding information synergistically (i.e., relying on the joint activity of the brain areas) whereas the NBG activity was mainly encoding information redundantly. Moreover, the BB activity preceded the NBG activity, indicating different roles, with BB relating mainly to initial stimulus processing, and NBG relating to maintenance of stimulus information over time. Although the sample is small, and will require replication for generalization, the demonstration of the result in both human and Macaque individuals is impressive, and lends credence to the identification of the separate roles for BB and NBG neural activity in visual sensory processing. In general, the methods are appropriate (although details are missing, which led to some confusion for me – see specific comments) and rigorous.

Response: We thank the reviewer for their thoughtful and constructive feedback. We are pleased that the replication across species was considered impressive and potentially impactful. Below, we address each of the reviewers' comments point by point.

(1) I presume that the BB activity identified is closely related to spi*ke rat*e and (sparse?) population coding (cf. Rolls & Treves, Prog. Neurobiol., 2011), although this is not made explicit. More could be said about this and its relation to oscillatory activity – perhaps in the context of the work of Akam and Kullman (which is cited). Or some other conceptualization of BB activity could be developed.

Response: We thank the reviewer for this insightful comment regarding the physiological interpretation of the broadband (BB) signal and its relation to neuronal firing rate and population coding. We have now expanded the Discussion to clarify that the BB component of the ECoG signal is widely interpreted as an aggregate correlate of asynchronous population firing rather than a narrowband oscillation:

Lines 343 - 364: *"We note that BB activity is conventionally defined empirically based on a lack of spectral peaks in the PSD and a 1/f structure in the PSD (Miller et al., 2009; Donoghue et al., 2020); we contrast BB with oscillatory activity, which is reflected by clear spectral peaks in the PSD caused by frequency-band-limited synchronous activity. Studies indicate that BB activity primarily reflects the contribution of aperiodic synaptic currents. These aperiodic currents contain contributions of both uncorrelated and correlated synaptic inputs (Einevol et al., 2007; Linden et al., 2009). At higher frequencies, spikes may also directly contribute energy to the LFP, particularly when the neurons are located near the electrode (Anastassiou et al., 2015; Buzsáki et al., 2012). The energy of BB activity is expected to correlate with firing rates, which matches empirical reports (Ray and Maunsell, 2011). Importantly, BB and narrowband oscillatory activity overlap in frequency space and thus require decomposition techniques to be disentangled. This is possible because of*

their different underlying generative processes and because they show differential correlations with various stimulus factors (e.g., Ray & Maunsell, 2011; Peter et al., 2019; Uran et al., 2022; Hermes et al., 2015; Jia et al., 2013).”

References:

- Anastassiou, C. A., Perin, R., Buzsáki, G., Markram, H., & Koch, C. (2015). Cell type-and activity-dependent extracellular correlates of intracellular spiking. *Journal of neurophysiology*, *114*(1), 608-623.
- Buzsáki, G., Anastassiou, C. A., & Koch, C. (2012). The origin of extracellular fields and currents—EEG, ECoG, LFP and spikes. *Nature reviews neuroscience*, *13*(6), 407-420.
- Einevoll, G. T., Pettersen, K. H., Devor, A., Ulbert, I., Halgren, E., & Dale, A. M. (2007). Laminar population analysis: estimating firing rates and evoked synaptic activity from multielectrode recordings in rat barrel cortex. *Journal of neurophysiology*, *97*(3), 2174-2190.
- Hermes, D., Miller, K. J., Wandell, B. A., & Winawer, J. (2015). Stimulus dependence of gamma oscillations in human visual cortex. *Cerebral cortex*, *25*(9), 2951-2959.
- Jia, X., Xing, D., & Kohn, A. (2013). No consistent relationship between gamma power and peak frequency in macaque primary visual cortex. *Journal of Neuroscience*, *33*(1), 17-25.
- Lindén, H., Pettersen, K. H., & Einevoll, G. T. (2010). Intrinsic dendritic filtering gives low-pass power spectra of local field potentials. *Journal of computational neuroscience*, *29*(3), 423-444.
- Miller, K. J., Sorensen, L. B., Ojemann, J. G., & Den Nijs, M. (2009). Power-law scaling in the brain surface electric potential. *PLoS computational biology*, *5*(12), e1000609.
- Peter, A., Uran, C., Klon-Lipok, J., Roese, R., van Stijn, S., Barnes, W., ... & Vinck, M. (2019). Surface color and predictability determine contextual modulation of V1 firing and gamma oscillations. *elife*, *8*, e42101.
- Ray, S., & Maunsell, J. H. (2011). Different origins of gamma rhythm and high-gamma activity in macaque visual cortex. *PLoS biology*, *9*(4), e1000610.
- Uran, C., Peter, A., Lazar, A., Barnes, W., Klon-Lipok, J., Shapcott, K. A., ... & Vinck, M. (2022). Predictive coding of natural images by V1 firing rates and rhythmic synchronization. *Neuron*, *110*(7), 1240-1257.
- Donoghue, T., Haller, M., Peterson, E.J. *et al.* (2020) Parameterizing neural power spectra into periodic and aperiodic components. Nature Neuroscience 23, 1655–1665.

(2) The statement of results before the methods (as is the convention for this journal) makes this paper somewhat difficult to understand – so perhaps a bit more method detail in the results, especially on how the MI and co-I were calculated would help. This applies especially to the “contrast” between gratings and noise (or gratings and natural stimuli). I have made several specific comments below on this. At the least the relevant equations, exactly as calculated, could be given in the methods.

Response: We thank the reviewer for the opportunity to improve the description of MI and co-I in the results. The addition of equations concerning the reviewer’s comment has been addressed in the "Specific Comments” section from the reviewer. We have included in the results sections further clarifications and pointers to the corresponding additions in the Results sections:

Line 161-168: *“Specifically, we computed MI between the neural response R (BB or NBG) and the stimulus category S (‘gratings’ vs. ‘noise’ in humans; ‘grating’ vs natural image in macaque). MI quantifies, in bits, the reduction in uncertainty about S given R and was estimated with the Gaussian Copula MI framework (see Methods). This provides a continuous measure of category discriminability and is evaluated at each time point per electrode with FWER-controlled permutation testing.”*

Line 194-198: *“Co-I tests whether two responses carry the same information (redundant) or if the relationship between them carries extra (synergistic) information about the stimulus: positive values indicate redundancy; negative values indicate synergy (see Methods and Figure 1).”*

(3) Although the work of Donoghue and Voytek is cited (once for a tangential reason), their approach to separating BB (in their case 1/f^n) from narrow band (not just gamma) activity is not mentioned. I wonder whether the conclusions would be the same using their approach, which involves scraping the narrow band “bump” off of the 1/f-like broadband PSD. I note that the BB

activity does not seem to be 1/f-like (Fig. 2A) whereas the NBG does appear to have this character for higher frequencies. At the least it could be mentioned that theirs is a different approach that might be complementary or even highly similar to the PCA approach taken here. (Note, I am not in any way affiliated with that group).

Response: We thank the reviewer for their comments. We have improved the Methods section to clarify this technical issue. The FOOOF method (Donoghue et al., 2020) is indeed a general approach to separate narrowband and broadband features in the power spectrum. It is a generalization of previous approaches used to separate gamma and broadband activity in a power spectrum, which we developed in previous studies (Hermes et al., 2015). The key issue with these methods in the current study is that the FOOOF method is not a signal decomposition method but rather a method for quantifying the strength of oscillatory activity based on average spectra, i.e. it decomposes the power spectrum into different components. However, for our study, it is required to decompose the signals on a trial-by-trial basis into BB and NGB activity. In other words, the 1/f + Gaussian fit does not generalize to power spectra that vary over small time segments. In our previous work, the 1/f + Gaussian model was fit to power spectra averaged across multiple trials (e.g., Hermes et al., 2019), and the FOOOF approach also operates across larger time windows. A reliable 1/f fit and Gaussian over small time segments becomes noisy, and we therefore opted for the PCA approach in the current study, which is a well-motivated signal decomposition method. We clarify in the Methods that:

Line 736-758: *"Many standard methods that extract spectral power in predefined frequency bands cannot reliably dissociate broadband (BB) activity from narrowband oscillations (e.g., NBG). For instance, estimating gamma-band power in the 30-80 Hz range with a bandpass filter will generally mix two distinct contributions: (i) genuine NBG oscillations and (ii) the portion of the broadband component that falls within 30-80 Hz (Donoghue et al., 2020; Miller et al., 2009). Moreover, several recently proposed approaches are optimized for power spectra that are averaged across trials (Hermes et al., 2015; 2019) or computed over relatively long time windows (Donoghue et al., 2020). These methods are therefore less suited when fine-grained temporal precision is critical for computing information representations, as in the present study. In contrast, the Spectral Decoupling method (Miller, 2019; Miller et al., 2009; Gelens et al., 2024; Canales-Johnson et al., 2021; Sabra et al., 2019) based on spectral principal component analysis (SPCA) approach operates at the level of trial- and time-resolved spectra and exploits covariance across frequencies to identify components that capture independent fluctuations in broadband and narrowband activity. This makes SPCA particularly well suited to disentangle BB and NBG contributions with the temporal precision required for our analyses."*

References:

- Hermes D., Miller KJ., Wandell BA., Winawer J (2015) Stimulus Dependence of Gamma Oscillations in Human Visual Cortex. Cerebral Cortex, 25(9), 2951–2959.
- Hermes D., Petridou N., Kay KN., Winawer J (2019) An image-computable model for the stimulus selectivity of gamma oscillations, eLife 8:e47035.
- Donoghue, T., Haller, M., Peterson, E.J. *et al.* (2020) Parameterizing neural power spectra into periodic and aperiodic components. Nature Neuroscience 23, 1655–1665.

(4) The values of MI and co-I found are rather small, in the range of 0.1 to 0.3 bit. It would be good to see a comment about the real significance of these amounts of information. Do they represent poor, fair, good or excellent stimulus discrimination in these brain areas?

Response: We appreciate the reviewer's point regarding the effect size. We have improved the manuscript to clarify that the effect size is not small. While 0.1-0.3 might seem a low number on an

absolute scale (i.e., if a reader is more familiar with effect sizes like d'), we don't think that is true in this study. A key advantage of information theory is that it allows a range of quantitative statistical assessments on the same effect size scale of bits. This is the core unit of information, one bit corresponds to a halving of the uncertainty, or alternatively to one yes-no question which splits the space of possibilities into two halves. It's also important to keep in mind that our measure of information in bits, is the average reduction in uncertainty expected on a single trial, from a single sample (MI is calculated per sample). Here the sampling rate is 500 Hz, so 0.1 bits/sample corresponds to around 50 bits/second (assuming independence across samples), indicatinghigh information-bearing neural signals. Note, this is just a rough guide to motivate the scale - accurately estimating information rates is a topic of active research and requires computationally intensive analysis over multiple temporal resolutions. Similarly, for comparison, we have used similar MI measures across a range of applications in neuroimaging (Ince et al 2017) including, face vs house contrast in EEG (arguably the strongest categorical ERP contrast) (Rousselet et al, 2014), speech entrainment in MEG (Daube et al. 2019), sampling of visual information with bubbles (Zhan, 2019), and prediction errors in ECoG recordings (Gelens et al., 2024). In these diverse applications, it is interesting that consistent, robust within-participant effects often fall in the range of 0.01-0.2 bits/sample. We report higher values here, but our data here is from invasive intracranial electrophysiology and therefore would expect a substantially higher signal to noise than non-invasive neuroimaging experiments. So we a priori don't agree that these values are very small. We have now added to the Methods the following description:

Line 876-886: *"Note that MI and co-I values are reported in units of bits. A value of 1 bit corresponds to a halving of the uncertainty of the trial state when observing the neural response. It is important to keep in mind, though, that these information values are the average per sample. Here, we use a sampling rate of 500Hz, so a value of 0.1 bits/sample corresponds to an approximate information rate of 50 bits/second (assuming independence) at that specific post-stimulus time point. However, this is just a heuristic comparison; formal estimation of information rates involves more extensive computation to quantify the limit of this information for higher and higher temporal resolution. "*

References:

- Ince, R.A.A., Giordano, B.L., Kayser, C., Rousselet, G.A., Gross, J. and Schyns, P.G. (2017), A statistical framework for neuroimaging data analysis based on mutual information estimated via a gaussian copula. Hum. Brain Mapp., 38: 1541-1573.
- Rousselet GA, Ince RA, van Rijsbergen NJ, Schyns PG (2014), Eye coding mechanisms in early human face event-related potentials. J Vis., 13:7. doi: 10.1167/14.13.7.
- Daube, C., Ince, R. A., & Gross, J. (2019). Simple acoustic features can explain phoneme-based predictions of cortical responses to speech. Current Biology, 29(12), 1924-1937.
- Zhan, J., Ince, R. A., Van Rijsbergen, N., & Schyns, P. G. (2019). Dynamic construction of reduced representations in the brain for perceptual decision behavior. Current Biology, 29(2), 319-326
- Gelens, F., Äijälä, J., Roberts, L, Komatsu M., Uran C., Jensen M., Miller KJ, Ince RAA, Garagnanai M., Vinck M., Canales-Johnson A. (2024) Distributed representations of prediction error signals across the cortical hierarchy are synergistic. Nature Communications 15(3941) (2024).

Specific:

1. Figures 3, 4, 5: error bars are very faint in MI plots and appear to be missing in some plots, although they may be smaller than the line thickness....

Response: Correct, they are not missing; they are smaller than the line thickness.

2. Page3: bottom left: "Within the framework of information theory, MI is a statistical measure that quantifies the strength of the dependence (linear or nonlinear) between two random variables. It can also be seen as the effect size, quantified in bits, for a statistical test of independence. Thus,

we used MI separately for the BB and NBG responses to quantify the difference between image categories on a common effect size scale.” Here is where it would be good to explain just how MI works as an effect size for differential responses to the gratings vs the noise stimuli. It’s because the Response-Stimulus MI goes both ways: I(R;S) = H(S) – H(S|R) = H(R) – H(R|S) = H(S) + H(R) – H(R,S) where H(x) is marginal entropy, H(x|y) is conditional entropy, and H(x,y) is joint entropy. More can be said in the Methods section.

Response: We thank the reviewer for this suggestion. We have now expanded the explanation in the Methods section to clarify how mutual information (MI) serves as an effect-size metric for quantifying differential responses to gratings versus noise stimuli within the information-theoretic framework.

Line 818-851: *“Within the framework of information theory, Mutual Information (MI) between the neural response (R) and the stimulus category (S) quantifies the reduction in uncertainty about one variable given knowledge of the other. Formally, MI can be written in several equivalent forms: I(R;S) = H(S) – H(S|R) = H(R) – H(R|S) = H(S) + H(R) – H(R,S), where H(X) denotes the marginal entropy of variable X, H(X|Y) the conditional entropy, and H(X,Y) the joint entropy. Intuitively, I(R;S) measures how much information (in bits) the neural response conveys about the stimulus category. In the present study, S represents the experimental contrast between grating and noise (or natural image) stimuli, and R corresponds to either the broadband (BB) or narrowband gamma (NBG) univariate neural response (i.e., single-electrode). Thus, MI provides a measure of effect size that quantifies how strongly neural activity discriminates between stimulus classes on a common information-theoretic scale. A value of 1 bit indicates that the response completely resolves the uncertainty between two equally probable stimulus categories, whereas smaller values indicate partial reductions in uncertainty.*

This formulation enables a direct comparison of BB and NBG responses in terms of their relative encoding strength. MI was estimated using the Gaussian Copula Mutual Information (GCMI) framework (Ince et al., 2017), which provides bias-corrected, robust (rank-based) estimates suitable for continuous neural data. Specifically, using all available trials per category contrast, the signal at every time point was permuted 200 times for each electrode, randomly assigning the stimulus class labels each time. The maximum value at each time point was taken, and the 95th percentile of this value was used as the threshold for significance. This method corrects for multiple comparisons and provides a Family-Wise Error Rate (FWER) of 0.05. The electrodes listed in the previous section were used separately to compare orientation versus unfiltered noise and orientation versus filtered noise for the BB and NBG signals.”

3. Page 11: Methods, Data acquisition: no information is given on the filtering of the ECoG data. This should be given. Also, re Macaque recordings: it seems rather odd to use a filter from 0.35 Hz to 7500 Hz to isolate LFPs. I guess this was the filter for the original data recording. More usual for LFPs is < 250 Hz (cf. Jia & Kohn, 2011). One will still get spikes, and also the LFP >100 Hz is mostly related to spike rate (Ray & Maunsell, 2011, PLoS Biology). This makes me wonder how much of the BB signal is spikes unless some unspecified filtering was also performed. More detail is needed in the description of these data.

Response: We thank the reviewer for pointing out the need for clarification. For the macaque recordings, we added the missing information in the Results section:

Lines 648-655: “*The data were filtered between 0.35 and 7500 Hz (3 dB filter cutoffs) and digitized at 24414.0625 Hz (TDT PZ2 preamplifier). Line noise was removed using a two-pass 4th order*

Butterworth bandstop filter between 49.9-50.1, 99.7-100.3, and 149.5-150.5Hz. We downsampled the LFPs to 1.02kHz using Matlab's decimate.m function with the default settings, which leads to a 3dB low-pass cut-off frequency of 406.90 Hz."

4. Page 11: Separating BB from NBG signals: Spectral decomposition: more details on the origin of the PSD covariance matrix (upon which the eigendecomposition was performed) should be given. What is covarying? It should not be necessary to go to the earlier papers to know at least this much about the method.

Response: We thank the reviewer for pointing out the need to clarify the origin and interpretation of the covariance matrix used in the spectral principal component analysis (SPCA). We have now expanded the corresponding section in the Methods to precisely describe which quantities covary and how the matrix is constructed from the power spectral density (PSD) estimates.

Lines 759-785: ***"****To separate broadband (BB) from narrowband gamma (NBG) activity, we applied spectral principal component analysis (SPCA) to the trial-wise power spectral densities (PSDs) obtained from each electrode. For each subject, we first computed the PSD of the ECoG signal for every trial and concatenated these spectra into a two-dimensional matrix (frequency × trials). Each column represents the spectral power profile of a single trial, and each row represents the distribution of power across trials at a given frequency. We then normalized the PSD at each frequency by dividing by the mean power across trials and log-transformed the resulting values to stabilize variance. From this normalized log-power matrix, we computed the covariance between all pairs of frequencies. Thus, each element of the covariance matrix reflects how fluctuations in power at one frequency covary with those at another frequency across trials. This frequency-by-frequency covariance matrix was then decomposed into eigenvectors, yielding orthogonal spectral principal components (SPCs) ordered by the variance they explain. The first SPC typically captured broadband (aperiodic) modulations spanning 1–200 Hz, while the second SPC isolated narrowband gamma activity (30–80 Hz) with a characteristic spectral peak (****Figure S1****). For each component, we reconstructed the corresponding time-domain signal by weighting and summing the original ECoG time series according to the component's loading vector, thereby obtaining separate BB and NBG time courses for subsequent analyses."*

5. Page 11, Stimuli and task: "These patterns varied across well-controlled image dimensions, including special aperture, orientation, contrast, sparsity, and coherence (Hermes et al., 2019)." Did you mean "spatial" aperture?

Response: Yes, we meant "spatial" aperture, thanks. Corrected in the main text now.

6. Page 12, Mutual Information analyses: More detailed information on these analyses, including equations (similar to that in the co-I section), needs to be added here and referred to directly in the main Results section. It is difficult to figure out from the general statements in the results section about MI and Co-I exactly how they were calculated in each analysis/Figure. What was the equation actually used for MI for each of the analyses? Finally, the MI calculated in the GCMI toolbox needs a bit more detailed explanation. The Gaussian cupola method is complex – and quite different from the direct binning or the Kraskov nearest neighbour method - and the Methods section is where at least the major steps could be outlined.

Response: Full details of the methods are given in the cited references, which also serve as a tutorial introduction. It is not really possible to cover the full details of a method published 8 years ago and cited hundreds of times (Ince et al., 2017), so we encourage readers to consult the

original publication for details, as is common in scientific practice. However, we have emphasised some key aspects of the Gaussian copula in the Methods:

Line 852-853: *“GCMI is a semi-parametric estimator that first transforms the data to have a Gaussian marginal distribution, then uses a Gaussian parametric estimation method. Data are rank transformed to obtain the marginal empirical cumulative density function, which is transformed to Gaussian values.*

Co-I is formally computed as: $co\text{-}I(X; Y; S) = I(X; S) + I(Y; S) - I(X, Y; S)$*”*

………..
Reviewer #2 (Remarks to the Author):

This work from Roberts et al. investigates the contribution of broadband (BB) and narrowband gamma (NBG) activity to visual processing in the cortex using information theory analysis of ECoG and LFP recordings from human and macaque visual areas. The authors apply spectral principal component analysis (SPCA) to separate BB and NBG components and report that BB carries early synergistic information, while NBG reflects sustained redundancy. These findings are interpreted as evidence for distinct functional roles, with BB supporting nonlinear integration and NBG reflecting linear and redundant information transfer.

The topic is timely and relevant, and the question of how different spectral components contribute to information coding is of general interest. However, I am not fully convinced that the analysis is robust enough to support the authors main conclusions. SPCA has been used previously to separate broadband from narrowband signals but there is no clear conceptual justification in the manuscript for why two spectral PCs should cleanly isolate oscillatory from non-oscillatory activity, and no additional validation of this claim is provided. Several electrodes are also excluded without any quantitative explanation, and no NBG components could be extracted from the V4 macaque data. This raises concerns about the reliability and specificity of the method which is problematic since its the foundation for all subsequent conclusions. As discussed below, I also find the interpretation of the second component as narrowband gamma insufficiently supported by the data.

The dataset is limited to three recordings, with low electrode counts in the human data. Statistical comparisons also lack key details such as the sample sizes for statistical tests and whether tests are done across trials or electrodes. Lastly, some core claims, such as the distinction between diagonal and sustained redundancy, are not convincingly demonstrated in the figures. In fact, the NBG component often shows stronger diagonal structure (even before stimulus onset), which appears inconsistent with the authors' interpretation.

In summary, while the manuscript presents an interesting idea, the methodological limitations and ambiguity of the results currently limit the impact of the work. Substantial extensions and clarifications are needed to strengthen the conclusions and support the proposed dissociation between BB and NBG signals. Below are my main points to help improve the impact of the work.

Major points:

1. The contrast between stimulus categories is based on a predictability measure derived from a deep neural network. While this is an interesting approach, the categories used (gratings, noise, natural images) differ in several respects beyond spatial homogeneity. It is therefore not clear whether the observed effects truly reflect differences in image homogeneity or other factors. For example, gratings are known to strongly drive gamma-band activity and elicit stereotyped responses across trials and noise is different from complex natural images or textures. The authors should therefore consider more carefully matched stimulus comparisons or at least acknowledge that homogeneity may not be the only relevant factor. Current claims, such as 'The finding that BB and NBG signals encode information about image homogeneity' are, in my view, not supported by the results.

Response: We thank the reviewer for their comment. We first want to clarify an important point: the central topic of the present study is not the stimulus correlates of NGB - unfortunately, the beginning of the results section could have given this impression.

The stimulus correlates of NGB have been extensively studied in much previous work. This prior work (which is further supported by the present study) has highlighted that a key factor to predict NGB is the structural predictability of local image patches given the surround, i.e. the extent to which the image in the surround predicts the center, which relates to the homogeneity of orientation across space (Uran et al., 2022, Hermes et al., 2019, Vinck & Bosman, 2016, Ray paper, 2022, Coen-Cagli et al., 2012). This was extensively controlled for other image factors, such as luminance contrast, firing rates, spatial frequency, etc., in Uran et al. (2022), who showed that, among many image features, structural predictability is the best predictor of NGB.

In this study, we stick to the neutral descriptor **homogeneity**, which we define in the Results (see below).

Importantly, the key topic of the present study is the information redundancy and information synergy in BB and NGB signals. Studying information redundancy and synergy requires examining a stimulus dimension for which NGB contains information. This motivated us to examine stimulus homogeneity, enabling us to further investigate redundancy and synergy and to compare them with BB signals.

To make this more apparent to the reader, we have revised the start of the Results section:

Lines 83-108: *"We focused on a feature of images that is known to be reliably encoded by NGB in both humans and macaques, namely the structural homogeneity of images (Hermes et al., 2019; Shirhatti et al., 2022; Uran et al., 2022; Vinck and Bosman, 2016). We operationalize homogeneity as the extent to which the image in the surround predicts the precise structure of receptive field inputs in the center (Uran et al., 2022), which relates to the homogeneity of structural features such as orientations across space (Coen-Cagli et al., 2012; Hermes et al., 2019). Uran et al. (2022) have shown that, among many image features, structural predictability is the best predictor of NGB. This approach allowed us to compare the nature of information representations between BB and NBG in terms of synergy and redundancy.*

In both humans, we analyzed a previously reported dataset of bandpass-filtered grayscale images that varied along several parametric dimensions. We compared gratings to a set of images with low spatial homogeneity. Using a deep neural network (DNN) to predict the visual input in the center from the surround (similar to Uran et al., 2022), we verified that gratings had higher homogeneity than noise images (S1: $t(1,110) = 4.101$, $p < 0.001$; S2: $t(1,94) = 3.08$, $p = 0.002$). In macaques, we analyzed gratings and grayscale natural images previously reported in macaques by Uran et al. (2022). We compared gratings to a set of natural images that had low homogeneity, as identified with the DNN (difference gratings and natural images: $t(1,470) = 24.45$, $p < 0.001$)."

And in the Methods section:

Lines 690-719: *"LPIPS (Learned Perceptual Image Patch Similarity) predictability scores measure how different the predicted center of an image is from its true center, based on the surrounding context. Low scores indicate that the prediction is similar to the true center. High scores mean the prediction is dissimilar to the true center. Hence, according to our definition of image homogeneity, low scores indicate high homogeneity, while high scores indicate low homogeneity.*

Thus, to identify images with the highest and lowest homogeneity in human subjects S1 and S2, we first ranked all images by their predictability scores. Oriented grating images (80 images)

showed the lowest scores (i.e., high homogeneity). Noise images (80 images) showed the highest scores (i.e., low homogeneity). We then selected these two image categories for further analysis. As a result, the MI and co-I analyses were performed on 80 gratings and 80 noise trials per subject. To confirm that the predictability scores resulted in significant differences between categories, we used a two-sided t-test to compare the scores of the pre-selected images. Gratings showed the highest image homogeneity (i.e., lowest scores) in S1 (mean = 0.171; SD = 0.081) and S2 (mean = 0.179; SD = 0.082), and noise showed the lowest homogeneity (i.e., highest scores) in S1 (mean = 0.343; SD = 0.304) and S2 (mean = 0.307; SD = 0.274) (Figure 1B). As expected, we observed higher predictability for gratings compared to noise images across images and electrodes in S1 ($t(1,110) = 4.101$, $p < 0.001$) and in S2 ($t(1,94) = 3.08$, $p = 0.002$).

Unlike the human participants in the original study by Uran et al. (2022), the macaque M1 was shown a set of natural images across 20 sessions. A total of 340 natural images were presented (20 trials per image). To identify images with the highest and lowest homogeneity in M1, we used LPIPS to determine the highest and lowest predictable natural stimuli in each session. This resulted in 80 gratings (20 images x 4 sessions = 80 images) and 80 natural-image trails (4 images with the lowest scores per session x 20 sessions = 80 images) being selected for further MI and co-I analyses. When comparing the predictability scores, we observed higher homogeneity for gratings (mean = 0.009; SD = 0.013) than for natural images (mean = 0.244; SD = 0.146) across images and electrodes ($t(1,470) = 24.45$, $p < 0.001$).”

References:

- Hermes, D., Petridou, N., Kay, K. N., & Winawer, J. (2019). An image-computable model for the stimulus selectivity of gamma oscillations. *eLife, 8*, e47035. https://doi.org/10.7554/eLife.47035
- Shirhatti, V., Ravishankar, P., & Ray, S. (2022). Gamma oscillations in primate primary visual cortex are severely attenuated by small stimulus discontinuities. *PLOS Biology, 20*(6), e3001666. https://doi.org/10.1371/journal.pbio.3001666
- Uran, C., Peter, A., Lazar, A., Barnes, W., Klon-Lipok, J., Shapcott, K. A., Roese, R., Fries, P., Singer, W., & Vinck, M. (2022). Predictive coding of natural images by V1 firing rates and rhythmic synchronization. *Neuron, 110*(7), 1240–1257.e8. https://doi.org/10.1016/j.neuron.2022.01.002
- Vinck, M., & Bosman, C. A. (2016). More gamma more predictions: Gamma-synchronization as a key mechanism for efficient integration of classical receptive field inputs with surround predictions. *Frontiers in Systems Neuroscience, 10*, 35. https://doi.org/10.3389/fnsys.2016.00035
- Coen-Cagli, R., Dayan, P., & Schwartz, O. (2012). Cortical surround interactions and perceptual salience via natural scene statistics. *PLOS Computational Biology, 8*(3), e1002405. https://doi.org/10.1371/journal.pcbi.1002405

2. One way to clearly demonstrate that image homogeneity is a driving factor would be to present noise or natural images with different image homogeneity and show that neural responses change, depending on the amount of homogeneity in a given stimulus. The results of the DNN are also not well explained: Why are the reported mean values low if homogeneity is high and what is distribution across stimuli. Using the DNN results to relate them to the neural data and information theory results could be helpful to support the authors claims.

Lastly, the exact type of gratings and noise stimuli should be described more clearly in the text. Why do the redundancy results differ between S1, S2 and M1? Where different stimuli shown in each experiment?

Response: We refer to our response to **point 1** of Reviewer 2. The aim of this study is not to reveal a novel neural correlate of NBG, but to study the synergistic/redundant structure of information representation. Our study builds on previous work by Hermes et al. (2019), Uran et al. (2022), and Shirhattan et al. (2022). Uran et al. (2022), for example, show a monotonous correlation between NBG amplitude and the structural predictability of images.

We have clarified the values reported for homogeneity. These reports, in fact, concern the perceptual loss in a DNN that predicts the center from the surround, which is why the values, as

the reviewer notes, indeed appear non-intuitive. We have reported these means/SDs in the Methods now and explained this. We have also explained the stimuli more clearly. Indeed, S1 and S2 use the same stimuli, but M1 uses different stimuli. This was already described in the original results and methods, but we have reemphasized it.

3. The entire analysis depends on separating BB and NBG signals using SPCA, yet the manuscript provides no quantitative validation that the resulting components indeed correspond to the desired signal types. In fact, SPCA is described with little conceptual detail or critical evaluation that the approach works as intended. For example, it is unclear why the first and second components should always reflect BB and NBG, respectively, or why it unnecessary to consider additional components to capture all relevant oscillatory activity. There is also no attempt to verify the frequency content of the resulting components by using more conventional spectral analyses. In fact, the claim that the NBG largely reflects gamma activity between 30-80Hz is hard to follow from the traces in Figure 2A. To me, it seems like most power is below 50 Hz and might also be particularly high in the alpha and beta range. Figure 2A also has very sparse x-axis labels, making it difficult to assess the exact frequencies where the NBG peaks are located. If the authors feel strongly that their analysis effectively isolates NBG activity, this should be demonstrated more clearly.

Response: We thank the reviewer for this comment. We have improved the explanation of the method and added additional analyses to address these points. First, we improved our description of the method, which has been used in multiple previous studies, and we employed it for the quantitative validation of BB and NBG components and electrode selection:

1. We have now added the following explanation in the Methods section, and two supplementary Figures (**Figure S1 and S2**) depicting the results of the SPC quantification:

Line 736-816: "*Many standard methods that extract spectral power in predefined frequency bands cannot reliably dissociate broadband (BB) activity from narrowband oscillations (e.g., NBG). For instance, estimating gamma-band power in the 30-80 Hz range with a bandpass filter will generally mix two distinct contributions: (i) genuine NBG oscillations and (ii) the portion of the broadband component that falls within 30-80 Hz (Donoghue et al., 2020, Miller et al., 2009). Moreover, several recently proposed approaches are optimized for power spectra that are averaged across trials (Hermes et al., 2015; 2019) or computed over relatively long time windows (Donoghue et al., 2020). These methods are therefore less suitable when fine-grained temporal precision is critical for computing information representations, as in the present study. In contrast, the Spectral Decoupling method (Miller, 2019, Miller et al., 2009, Gelens et al., 2024, Canales-Johnson et al., 2021, Sabra et al., 2019) based on spectral principal component analysis (SPCA) approach operates at the level of trial- and time-resolved spectra and exploits covariance across frequencies to identify components that capture independent fluctuations in broadband and narrowband activity. This makes SPCA particularly well suited to disentangle BB and NBG contributions with the temporal precision required for our analyses.*

To separate broadband (BB) from narrowband gamma (NBG) activity, we applied the "Spectral Decoupling" method by computing the spectral principal component analysis (SPCA) on the trial-wise power spectral densities (PSDs) obtained from each electrode. For each subject, we first computed the PSD of the ECoG signal for every trial and concatenated these spectra into a two-dimensional matrix (frequency × trials). Each column represents the spectral power profile of a single trial, and each row represents the distribution of power across trials at a given frequency. We then normalized the PSD at each frequency by dividing by the mean power across trials and

log-transformed the resulting values to normalize variance. From this normalized log-power matrix, we computed the covariance between all pairs of frequencies. Thus, each element of the covariance matrix reflects how fluctuations in power at one frequency covary with those at another frequency across trials. This frequency-by-frequency covariance matrix was then decomposed into eigenvectors, yielding orthogonal spectral principal components (SPCs) ordered by the variance they explain. In our dataset, the first SPC captured broadband (aperiodic) modulations spanning 1–200 Hz, while the second SPC isolated narrowband gamma activity (30–80 Hz) with a characteristic spectral peak (see next paragraph). For each component, we reconstructed the corresponding time-domain signal by weighting and summing the original ECoG time series according to the component's loading vector, thereby obtaining separate BB and NBG time courses for subsequent analyses.

*We identified NBG components using a run-length threshold applied independently to each spectral principal component (SPC) and electrode. For each SPC, we evaluated the frequency range 30–80 Hz (51 samples). An NBG component in a given electrode was marked present if this window contained a consecutive run spanning at least 75% of the window with magnitudes strictly greater than 0 (i.e., ≥ 39 consecutive samples within 30–80 Hz). Applying this criterion independently yielded a single NBG component in 4/7 electrodes in S1, 5/6 electrodes in S2, 63/63 electrodes in M1 (V1), and 0/63 electrodes in M1 (V4). Across S1, S2, and M1, the NBG component corresponded to SPC 2 in all cases. Similarly, the BB components were selected with the same procedure over 1–200 Hz (200 samples). A BB component in a given electrode was marked present if this window contained a consecutive run spanning at least 75% of the window with magnitudes strictly greater than 0 (i.e., ≥150 consecutive samples within 1–200 Hz). Using this criterion, we found a single BB component in 7/7 electrodes in S1, 6/6 electrodes in S2, and 63/63 electrodes in M1 in both V1 and V4. The BB component corresponded to SPC 1 across S1, S2, and M1. The average across channels for NBG and BB components following this procedure is depicted in **Figure S1**. The projection of SPC1 and SPC2 on the time-frequency axis for gratings stimuli for S1, S2, and M1 is shown in **Figure S2**."*

References:

- Canales-Johnson, A., Teixeira Borges, A. F., Komatsu, M., Fujii, N., Fahrenfort, J. J., Miller, K. J., & Noreika, V. (2021). Broadband dynamics rather than frequency-specific rhythms underlie prediction error in the primate auditory cortex. Journal of Neuroscience, 41(45), 9374–9391. https://doi.org/10.1523/JNEUROSCI.0367-21.2021
- Gelens, F., Äijälä, J., Roberts, L., Komatsu, M., Uran, C., Jensen, M. A., Miller, K. J., Ince, R. A. A., Garagnani, M., Vinck, M., & Canales-Johnson, A. (2024). Distributed representations of prediction error signals across the cortical hierarchy are synergistic. Nature Communications, 15, 3941. https://doi.org/10.1038/s41467-024-48329-7
- Jensen, M. A., Fine, A., Kerezoudis, P., Wong-Kisiel, L., Alden, E. C., Hermes, D., & Miller, K. J. (2025). Functional mapping of movement and speech using task-based electrophysiological changes in stereoelectroencephalography. Journal of Neurosurgery, 142(2), 311–323. https://doi.org/10.3171/2024.5.JNS24434.
- Miller, K. J., Zanos, S., Fetz, E. E., den Nijs, M., & Ojemann, J. G. (2009). Decoupling the cortical power spectrum reveals real-time representation of individual finger movements in humans. Journal of Neuroscience, 29(10), 3132–3137. https://doi.org/10.1523/JNEUROSCI.5506-08.2009
- Miller, K. J. (2019). A library of human electrocorticographic data and analyses. Nature Human Behaviour, 3(11), 1225–1235. https://doi.org/10.1038/s41562-019-0678-3
- Miller, K. J., Hermes, D., Pestilli, F., Wig, G. S., & Ojemann, J. G. (2017). Face percept formation in human ventral temporal cortex. Journal of Neurophysiology, 118(5), 2614–2627. https://doi.org/10.1152/jn.00113.2017.
- Sabra, Z., Bonilha, L., & Naselaris, T. (2020). Spectral encoding of seen and attended object categories in the human brain. Journal of Neuroscience, 40(2), 327–342. https://doi.org/10.1523/JNEUROSCI.0900-19.2019

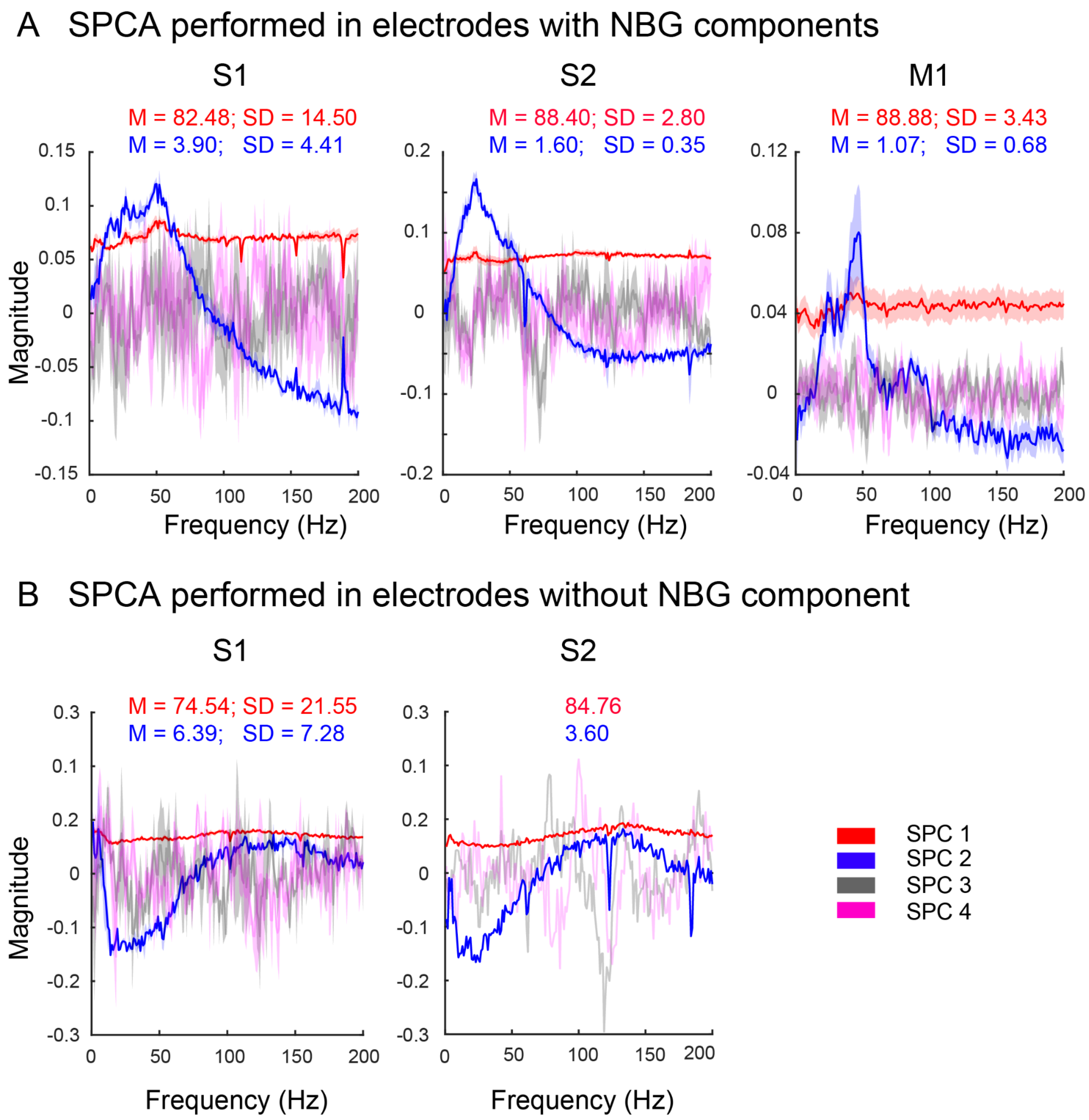

Figure S1. *SPCA component for S1, S2, and M1.* ***(A)*** *Averaged traces showing a characteristic NBG component for S1 (4 out of 7 electrodes), S2 (5 out of 6 electrodes), and M1 (63 out of 63 electrodes). While PC1 (red) does not show a characteristic peak across frequencies (BB component), PCA 2 (blue) shows increased magnitude spanning the gamma band (NBG). The mean and standard deviation of the variance explained by the PC1 and PC2 components across channels are depicted in red and blue, respectively. Unlike PC1 and PC2, PC3 (gray) and PC4 (magenta) exhibit high channel variability without a characteristic peak.* ***(B)*** *Averaged traces for electrodes without a characteristic NBG component for S1 (2 out of 7 electrodes), S2 (1 out of 6 electrodes). The mean and standard deviation of the variance explained by the PC1 and PC2 components across channels are depicted in red and blue, respectively. In contrast, PC1 shows a broadband component, and PC2 mainly peaks in the lower-frequency range (<10 Hz).*

2. To further verify the frequency content of SPC1 and SPC2 in a more conventional analysis, we back-projected SPC1 and SCP2 using the grating stimuli (as gratings elicit NBG, as shown in the new **Figures S1 and S3A** [see response to **point 5**]) and projected them into a time-frequency chart. The results of this analysis and description of the method used are now reported in the new **Figure S2**:

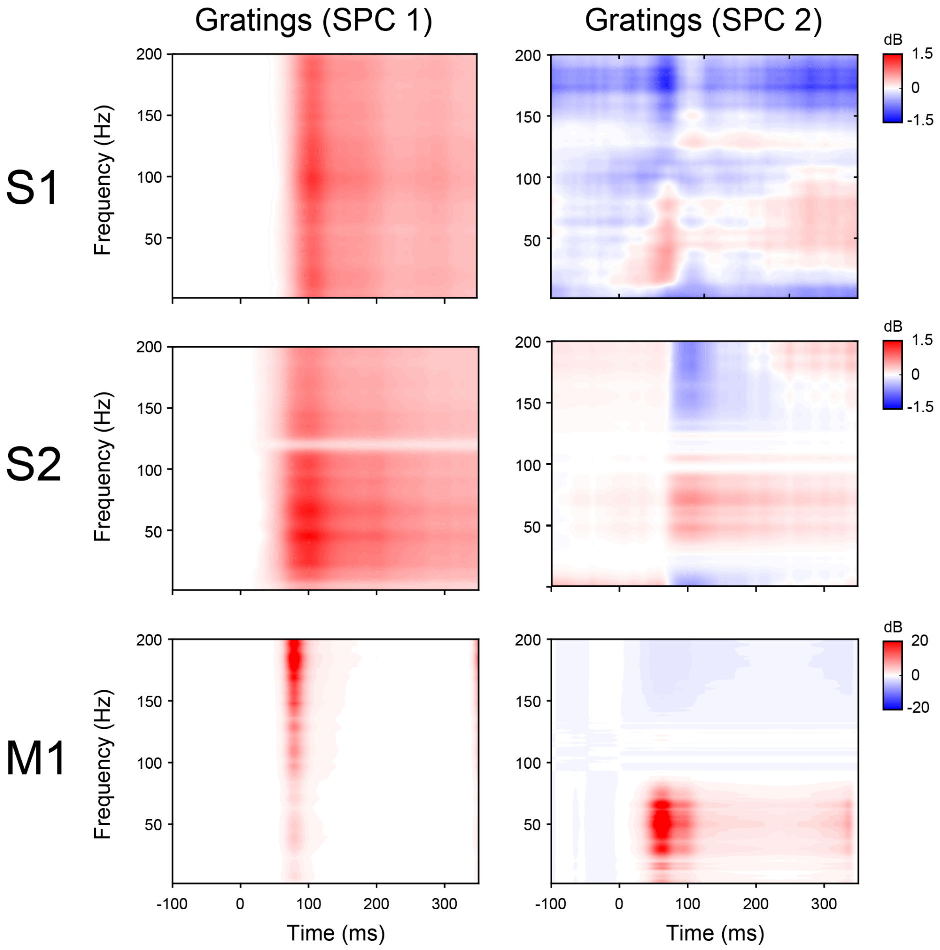

Figure S2. *Time–frequency spectral reconstruction using SPC1 and SPC2 in S1, S2, and M1. For each electrode and trial with grating stimuli, Welch power spectra were computed with a single full-length segment (nfft = 500), retaining positive-frequency bins up to 200 Hz. To capture spectral structure, spectra were log-normalized at each frequency, and spectral PCA (SPCA) was performed on the frequency–frequency covariance matrix. The first two components (SPC1 and SPC2) were treated as fixed spectral motifs. We then computed an SPC-specific time course: the signal was filtered with a bank of complex Morlet wavelets (5 cycles per frequency), the squared magnitude was taken to obtain band-power over time, convolution edge samples were discarded, each band's power trace was normalized to unit mean, a natural-log transform was applied, and the resulting log-power matrix was projected onto the SPC vector to yield a single time course for that component. To reconstruct absolute time–frequency (TF) power, we (1) paired this time course with the component's frequency profile to form a separable log-power map, (2) exponentiated that map to convert it into a multiplicative power modulation, and (3) multiplied, at each frequency, by the electrode mean Welch spectrum to return to absolute power units. We then baseline-normalized per frequency to −100–0 ms and reported the values in dB (10 log10 of the baseline ratio). Panels show TF maps averaged across channels. Power is expressed in decibels (dB) relative to the baseline period. While SPC1 spans most of the frequency axis, SPC2 shows non-zero power predominantly in the gamma range (30–80 Hz).*

4. Moreover, several electrodes were excluded from the analysis upon visual inspection, and no NBG activity was extracted in V4 of the macaque data. Given the substantial literature on visually induced gamma activity in macaque extrastriate cortex, this absence is surprising and calls into question whether the method is sensitive enough to reliably detect gamma activity (or oscillatory activity in general). The authors should at least provide a more rigorous validation of the SPCA results and explicitly compare them to alternative approaches (e.g., band-limited filtering or parameterized spectral decomposition) to resolve these issues.

Response: Thank you for the opportunity to explicitly report our findings on V4 and the literature discussing NBG in V4. We note that studies investigating gamma in V4 (and V1) usually optimize stimuli to induce narrow-band gamma. However, this was not done in the present macaque dataset. We note that several studies/datasets report V1 gamma, but not V4 gamma (e.g., Debes and Dragoi, 2023; Spyropoulos et al., 2024). This also includes data with grating stimuli (Spyropoulos et al., 2024). In other studies, V4 gamma was reported (Grothe et al., 2012; Vinck et al., 2013), although it is noted that the V4 gamma rhythm was reported to be substantially weaker than in area V1 (e.g., spike-field locking values are around 0.003 in area V4 but around 0.1 in area V1, Vinck et al., 2013; Onorato et al., 2020). The stimulus and the recording type both play essential roles. We also note that data from the Fries lab with simultaneous V1-V4 ECoG recordings and natural images does not reveal V4 gamma (Jarrod Dowdall, 2023, PhD thesis, Radboud Repository). Due to the lack of NBG in V4, as now shown in the new **Figure S4,** it was not possible to quantify redundant information between V1 and V4 in the macaque dataset.

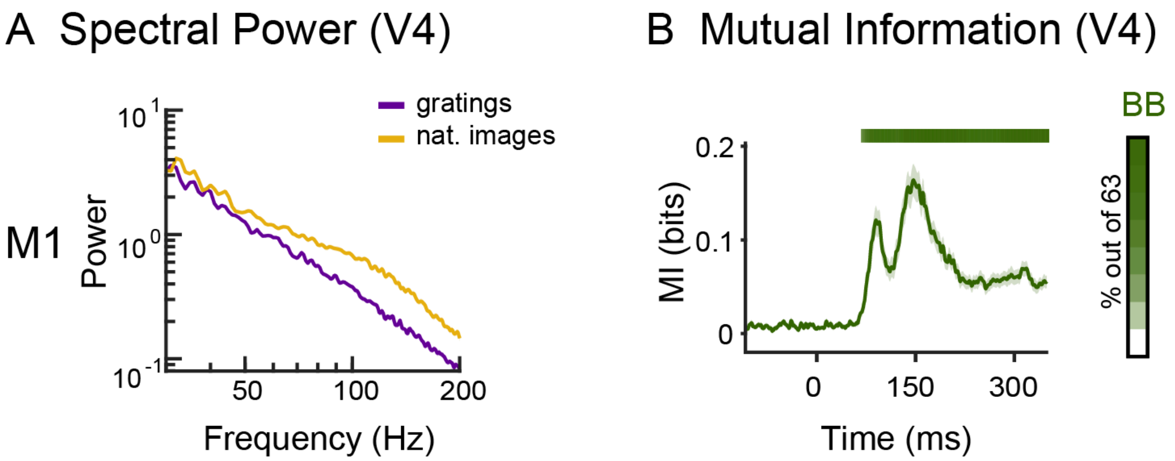

Figure S4. *Spectral power and mutual information for M1 in area V4.* ***(A)*** *Power spectral density for gratings (purple) and noise and natural images (yellow). Error bars represent the standard error of the mean (S.E.M) across electrodes in V4.* ***(B)*** *Mutual information analyses between gratings and natural images for M1 (dark green). Error bars represent S.E.M across electrodes. The shaded horizontal bars represent significant MI values at the single-electrode level, with the colors representing the percentage of electrodes showing a significant effect per time point for BB (dark green).*

Finally, to further show the reviewer the absence of NBG differences between gratings and natural images in V4, we have now also computed the MI between stimulus categories in the full time-frequency chart (i.e., the effect size of the difference in spectral power between gratings and natural images) in **Figure S5.**

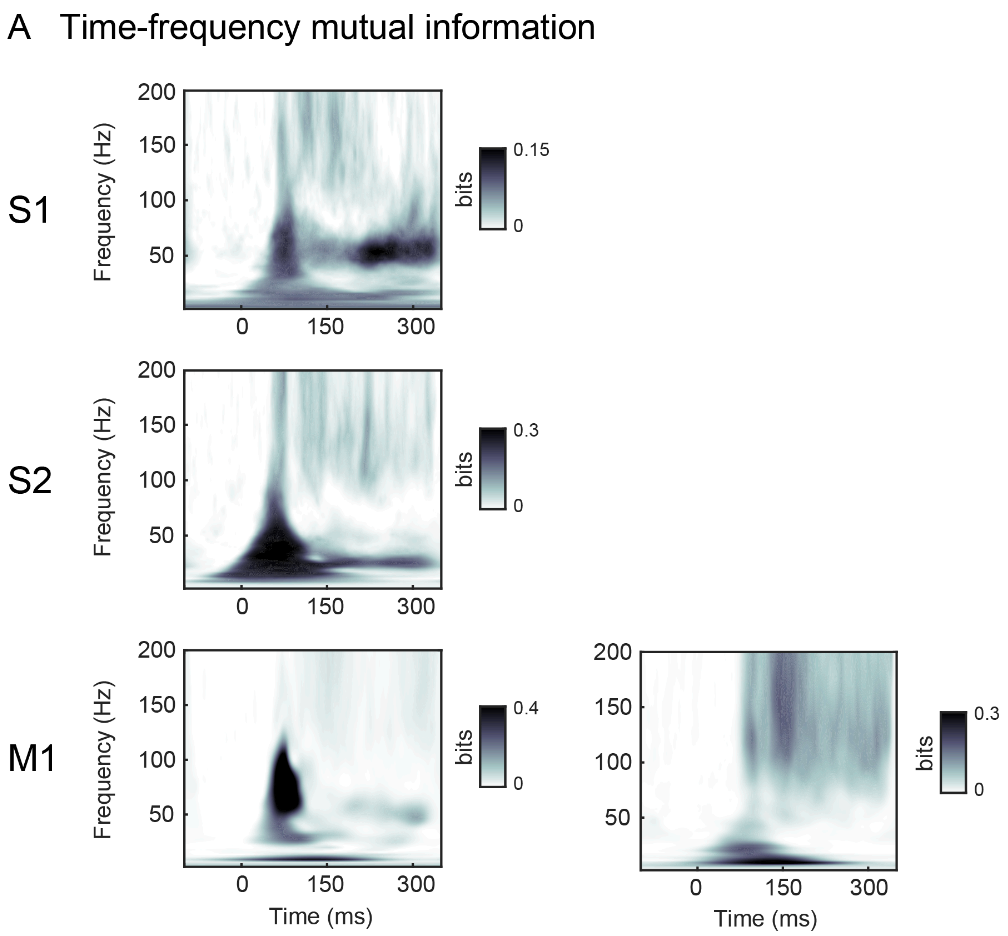

Figure S5. *Time-frequency mutual information for S1, S2, and M1. Mutual information (MI) between gratings and noise for S1 and S2, and between gratings and natural images for M1. MI was computed from power values across trials for each time-frequency bin from 1 to 200 Hz, within the -100 to 350 ms window around stimulus onset. MI represents the effect size (in bits) of the difference in spectral power for each corresponding stimulus comparison. Plots represent the average MI across electrodes (S1: 7 electrodes [V1 and V2 area]; S2: 6 electrodes [V1, V2 and V3 area], M1: 63 electrodes in V1 (left panel), and 63 electrodes in V4, right panel).*

References:

- Debes SR., Dragoi V (2023). Suppressing feedback signals to visual cortex abolishes attentional modulation.*Science* 379,468-473.
- Spyropoulos, G., Schneider, M., van Kempen, J., Gieselmann, M.A., Thiele, A., Vinck, M (2024). Distinct feedforward and feedback pathways for cell-type specific attention effects. *Neuron* 112, 2423-2434.
- Grothe I., Neitzel SD., Mandon S., Kreiter AK (2012). Switching Neuronal Inputs by Differential Modulations of Gamma-Band Phase-Coherence. *Journal of Neuroscience* 32, 16172-16180.
- Vinck M., Womelsforf T., Buffalo EA., Desimone R., Fries P (2013). Attentional Modulation of Cell-Class-Specific Gamma-Band Synchronization in Awake Monkey Area V4. *Neuron* 80, 1077-1089.

5. The dataset used in this study is small, consisting of only three recordings in total (two human, one macaque). The number of analyzed electrodes is also low, particularly in the human data. While this might be a limitation of the available data, it raises concerns about the robustness and generalizability of the results. Statistical tests are reported, but whether comparisons are across electrodes, sessions, or trials and basic information such as sample sizes is frequently missing. For example, Fig. 2 shows mutual information from individual electrodes, but it is not clear how these results generalize.

Response: Thank you for the opportunity to expand our analyses to include all available data to generalize the results. First, the nature of intracranial recordings means that, of course,

across-subject statistics are hard to obtain; hence, we have performed single-subject statistics, which is standard in the literature for this type of recording (e.g., Hermes et al., 2015; 2019; Gelens et al., 2024). We also want to mention that ECoG recordings in the occipital cortex in humans are rare (especially in V1). However, to demonstrate the generalizability of the results, we have now included two summary Figures (**Figure S3B** and **Figure S4B**) that show the MI effects observed in S1, S2, and M1, clarifying sample size and the dimensions where the tests were performed:

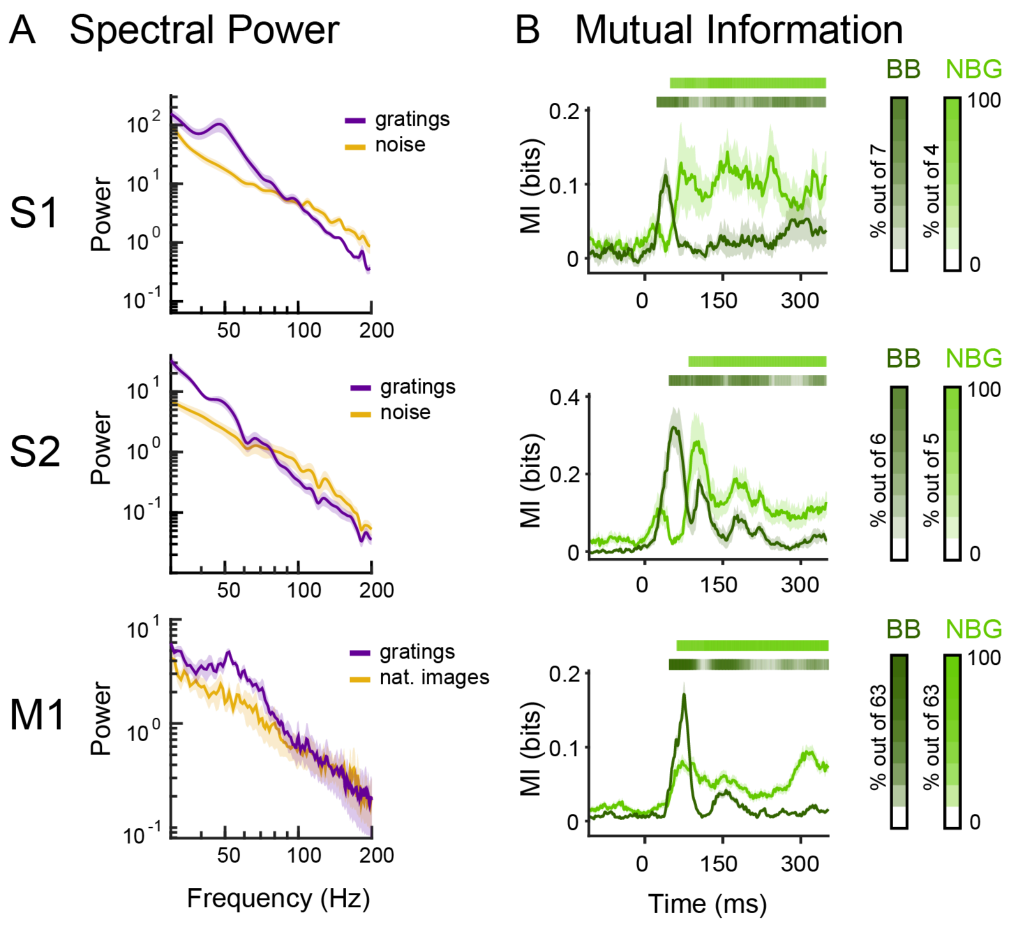

Figure S3. *Spectral power and mutual information for S1, S2, and M1.* ***(A)*** *Power spectral density for gratings (purple) and noise (yellow) stimuli for S1 and S2, and for gratings and natural images (yellow) for M1. Error bars represents the standard error of the mean (S.E.M) across electrodes (S1: V1,V2; S2: V1, V2, V3, and M1: V1).* ***(B)*** *Mutual information analyses between gratings and noise images for the BB component for S1 and S2 (dark green), and between gratings and natural images for M1 (dark green). Mutual information between gratings and noise images for the NBG component for S1 and S2 (light green), and between gratings and natural images for M1 (light green). The shaded horizontal bars represent significant MI values at the single-electrode level, with the colors representing the percentage of electrodes showing a significant effect per time point for BB (dark green) and NBG (light green). Error bars represent the S.E.M of the MI values across electrodes.*

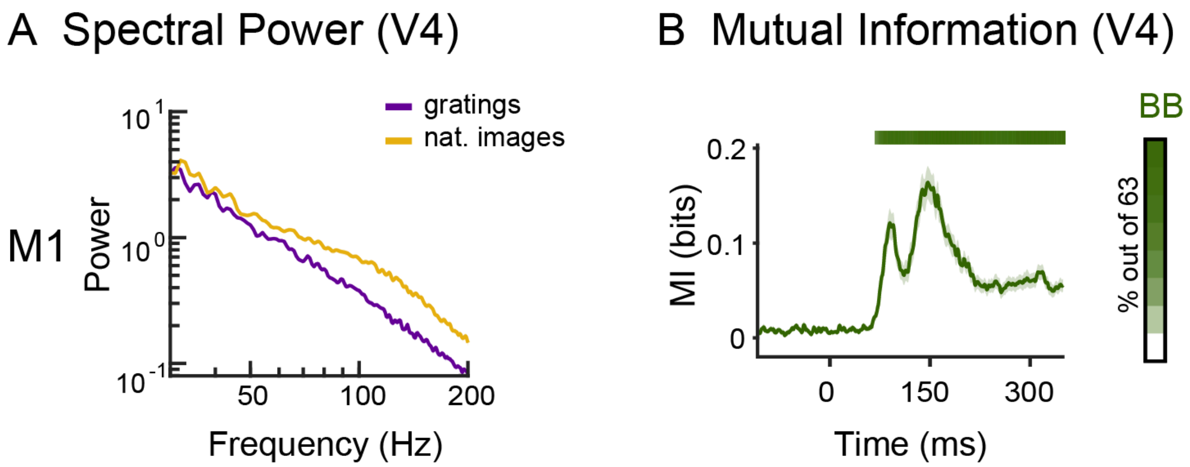

Figure S4. *Spectral power and mutual information for M1 in area V4.* ***(A)*** *Power spectral density for gratings (purple) and noise and natural images (yellow). Error bars represent the standard error of the mean (S.E.M) across electrodes in V4.* ***(B)*** *Mutual information analyses between gratings and natural images for M1 (dark green). The shaded horizontal bars represent significant MI values at the single-electrode level, with the colors representing the percentage of electrodes showing a significant effect per time point for BB (dark green). Error bars represent S.E.M of the MI values across electrodes.*

6. The interpretation of redundancy and synergy matrices is often ambiguous. The manuscript claims that BB signals show a temporally evolving redundancy profile along the diagonal, whereas NBG shows sustained redundancy over time. However, the actual co-information matrices (e.g., Fig. 3B, E or Fig. 5B) appear to show strong diagonal structure in the NBG component as well. If the authors want to support this distinction, they should separately quantify redundancy along and off the diagonal and show how these measures differ between signal types. Moreover, co-information in Fig. 5B is high even before the stimulus onset, which is difficult to reconcile with the idea that the signal reflects stimulus-driven information.

Response: We thank the reviewer for this important point and agree that interpreting diagonal structure by inspection can be ambiguous. In the revised manuscript we therefore clarified the temporal-generalization interpretation of co-information (co-I) matrices and added an explicit quantification. First, we now explain in the Results section that redundancy patterns are analogous to temporal-generalization "chains" versus "sustained" decoding (King et al., 2014): a narrow diagonal ridge indicates a continually evolving representation, whereas a broad off-diagonal block indicates a stable representation that generalizes across time; importantly, sustained redundancy will necessarily include a diagonal component, so the presence of a diagonal ridge in NBG does not contradict a sustained representation.

Consistent with this framework, BB co-I charts show redundancy largely confined around the diagonal, while NBG shows redundancy extending widely off-diagonal across most post-stimulus times (**Figure 3B,E; Figure 5B**). We believe our quantification of the extension of the significant effects support this distinction quantitatively: the temporal extension of redundancy as the normalized count (proportion) of significant positive co-I samples across the full time x time charts shows significantly more temporally extended redundancy in NBG than BB within areas and between areas (**Figure 3C,F**; **Figure 5C**; **Figure 4C,F**), confirming that NBG redundancy generalizes over time more strongly than BB.

Finally, regarding elevated pre-stimulus co-I in the unthresholded average matrix, we emphasize that all time series were baseline-corrected using −100–0 ms, and inference is based on

FWER-controlled permutation tests across all time×time points; thus pre-stimulus fluctuations that do not survive corrected significance are not interpreted as stimulus-driven. We have added text to make this explicit.

7. Both redundancy and synergy results in Fig. 6 are sustained over time, which is different from human results in Fig. 4, yet the authors describe them as consistent across species and different areas. This difference should be discussed and better explained. The statement in the discussion 'Notably, this broadband activity carried information especially early after stimulus onset, while oscillatory activity carried information substantially later' currently seems to go against the results shown in Fig. 6.

Response: We thank the reviewer for pointing this out. In **Figure 6** (macaque V1–V4), both synergistic and redundant BB interactions indeed extend over a larger portion of the time x time co-I charts than the human inter-areal BB interactions in **Figure 4**. We now discuss this explicitly. First, the macaque inter-areal analysis is necessarily restricted to BB–BB interactions, because no reliable NBG component was detected in V4 (0/63 electrodes; see Methods and Supplementary **Figure S4**), preventing a BB vs.NBG comparison across areas in M1. Second, the stimulus contrast differs across species (humans: gratings vs. noise; macaque: gratings vs. natural images), and in M1 and V4, BB MI shows relatively longer-lasting responses to this contrast compared to V1 and V2. Since co-I magnitude and extent are constrained by the time points at which each area carries MI, broader MI support in both areas naturally yields broader spatiotemporal co-I. Importantly, this ***does not*** contradict our cross-species conclusion about *which signal type carries which information*: wherever NBG is present (V1 in all subjects/species), it shows predominantly redundant, sustained representations, whereas BB tends to synergistic representations within and between areas. We have added a more nuanced part to the discussion:

Lines 324-339: *"We also note an apparent difference in the temporal extent of inter-areal BB interactions across species. In humans (**Figure 4**), BB synergy and redundancy between V1–V2/V3 are relatively confined in time, whereas in macaque (**Figure 6**) synergy between BB responses and redundancy between V1 and V4 span a broader post-stimulus window. Two factors likely contribute. First, in macaque V4, we did not detect a reliable NBG component (0/63 electrodes), so **Figure 6** reflects only BB–BB interactions, whereas **Figure 4** compares BB and NBG communication directly. Second, the macaque comparison (gratings vs natural images) produces relatively long-lasting MI in the BB response in area V4, and this increased MI in each area constrains spatiotemporal co-I. This may correspond to more temporally extended BB co-information without changing the qualitative conclusion that BB communication is primarily synergistic."*

8. The discussion presents a range of interesting ideas about the role of BB and NBG signals in nonlinear integration, resonance, and inter-areal communication. However, many of these claims are speculative and not clearly linked to the data. For example, the idea that BB reflects nonlinear recurrent dynamics and NBG reflects linear information transfer is not demonstrated directly. The interpretation of NBG as reflecting gamma oscillations is also questionable given the unclear spectral content. Overall, I think a more restrained interpretation of the findings would be appropriate unless stronger evidence can be provided to support these functional distinctions.

Response: We have addressed the point about NGB in our response to this and the other reviewer above. We agree that the latter part of the Discussion contains more speculative interpretations, although they are motivated by modelling studies. We have explicitly stated that these points are speculative.

Minor points:

Figure 2A lacks sufficient axis labeling to judge the frequency content of the BB and NBG components.

Response: We have added more ticks to **Figure 2A** for better interpretability of the frequency span, as well as to related new supplementary **Figure S1, S3** and **Figure S4**.

Figure references in the text are repeatedly mismatched (e.g., references to Fig. 1D seem to describe Fig. 1C. I dont think Fig. 1D is actually explained in the text). This should be corrected to improve clarity.

Response: Thanks. We have now corrected the repeatedly mismatched figures across the manuscript and explained **Figure 1D** in the Results.

Line 194-198: *"Co-I tests whether two neural responses carry the same information (redundant) or if the relationship between them carries extra (synergistic) information about the stimulus: positive values indicate redundancy; negative values indicate synergy (see Methods and Figure 1D)"*

It would be helpful to show the raw or filtered traces to Fig.2 to better illustrate how BB and NBG relate to the original data.

Response: Thanks. Raw PSD traces and are depicted now in **Figure S3A** and **Figure S4A**, and BB and NBG components across all channels and subjects are now depicted in **Figure S1**.

Please add individual data points for all electrodes to the box plots ,e.g. Fig. 3C.

Response: Thank you for the suggestion. After reviewing the initial figures with the dots included, we have decided against them as they look extremely cluttered. For example, in M1, there are plots with 63 x 63 = 3,969 points, which make them visually unintelligible.

The statement "While the BB response is induced by all images, including spatially heterogeneous images, the NBG response is predominantly induced by spatially homogeneous images such as gratings." does not seem match any of the results. Is this shown somewhere or only refers to other work?

Response: This statement is accompanied by citations and refers to prior work:

Lines 133-136: *"While the BB response is induced by all images, including spatially heterogeneous images (Hermes et al., 2015; 2019; Uran et al., 2022), the NBG response is predominantly induced by spatially homogeneous images such as gratings(Hermes et al., 2015; 2019; Uran et al., 2022)."*

It is not clear to me why the co-Information in Fig. 6 is so asymmetric. Why is there such strong difference when computing co-I between V1-V4 or vice versa? It would also be good to show statistical results for all electrodes (similar to earlier figures) to provide an overview if results are consistent.

Response: Co-information is non-directional and symmetric in its two neural variables: by definition, co-I(X;Y;S)=I(X;S)+I(Y;S)−I(X,Y;S), so exchanging X and Y (here V1 and V4) cannot change the values, it only transposes the time x time matrix. Thus, any apparent "asymmetry" in Figure 6 is not a V1 to V4 vs V4 to V1 effect of the estimator, but a consequence of different MI time courses in the two areas. In macaque BB, V1 carries stimulus information earlier and peaks sooner than V4 (**Figure 6,** MI traces), so the strongest co-I naturally occurs for time pairs that combine early informative V1 samples with later informative V4 samples, producing an off-diagonal synergistic lobe aligned with the feedforward delay. The opposite quadrant (early V4 with late V1) is weaker simply because those time pairs contain less MI to combine. We now clarify in the Results/Discussion that the observed structure reflects unequal timing of information across areas, not an order-dependence or directionality in co-information.

Lines 320-323: *"Note that co-I is non-directional; the apparent asymmetry reflects earlier V1 information and later V4 information, producing an off-diagonal synergistic pattern aligned with the feedforward delay."*

Regarding the quantification, please note that we did quantify these effects. Specifically, we quantify significance across all V1–V4 electrode pairs (63 × 63 = 3969 pairs) using the same FWER-controlled permutation framework, and report the proportion of pairs showing significant effects. We find that spatiotemporal redundancy is significant in 3597/3969 pairs (90.6%), overall co-information in 3686/3969 pairs (92.8%), and spatiotemporal synergy in 1721/3969 pairs (43.4%). These results show that a small subset of channels does not drive the patterns in Figure 6 but are broadly consistent across the electrode population. What we cannot do is to plot the direct comparison between BB and NBG synergistic and redundant effect since we did not observe gamma components in V4 (0/63 electrodes; Methods; **Figure S4**)

………
Reviewer #3 (Remarks to the Author):

The article by Roberts et al. examines how broadband signals and narrowband gamma oscillations contribute to the encoding of visual information. The authors analyzed electrocorticography (ECoG) recordings from two human subjects and local field potentials (LFPs) from one monkey and used information-theoretic approaches to examine whether signals convey redundant (shared) or synergistic (complementary) visual information related to image statistics namely, the spatial homogeneity of stimuli. Their analyses revealed that broadband signals encode synergistic information, whereas narrowband gamma oscillations primarily carry redundant information. Based on this the authors suggest that broadband dynamics subserve nonlinear mechanisms that serve pattern recognition, whereas narrow band oscillatory activity facilitates information sharing that can enhance robustness of encoded information. The paper is mostly well-written and puts forward an interesting proposal that can advance our understanding of neural coding. However, some questions pertaining to the generalization of the findings for visual information processing remain open. Additionally, there are several points where clarity could be improved to help the reader navigate through the analyses, results and their interpretation and appreciate their potential importance. We list below some suggestions toward this end.

Response: We thank the reviewer for their helpful feedback and constructive criticism. We appreciate that they found our manuscript well written and our conceptual proposal interesting. In the next section, we address the reviewers' comments point by point.

Major comments:

1. This may sound overly simplistic but the very idea of finding that a broadband (BB) signal which by definition holds a rich frequency content carries synergistic information does not sound extremely surprising. It is possible that I am missing something but it would help if the authors could interpret their results (and perhaps frame their hypothesis) taking into account how different the two signals (BB and narrowband) are and what kind of biases or limitations this difference imposes.

Response: We appreciate the reviewer's point and agree that differences in spectral content could, in principle, bias comparisons between BB and narrowband gamma (NBG). However, our synergy effects are not a trivial consequence of bandwidth. First, synergy is defined relative to the single-variable information terms and becomes negative only when the joint response carries *more* information than the sum of the parts; mere broadband power or SNR does not guarantee negative co-information. Second, BB and NBG were derived as separate one-dimensional time series via spectral decoupling (SPCA) and then analyzed identically with MI/co-I, so "more frequencies" do not directly enter the information calculations; what drives synergy, instead, is the trial-by-trial complementarity across time and recording sites, not the number of Fourier bins. We have clarified these points in the manuscript in the Discussion:

Line 561-574: "*One may argue that the BB component, by spanning a wide frequency range, necessarily contains more information or synergy. This is unlikely for two reasons. First, synergy depends on excess joint information beyond the sum of individual MIs. It therefore cannot be produced by a higher signal-to-noise ratio (SNR) or wider spectra alone. Second, our pipeline reduces both BB and NBG to a matched one-dimensional time series before the MI and co-I computations. Hence, the information measures capture trial-by-trial complementarity in the time domain rather than the spectral domain. Consistent with this, we observed BB off-diagonal synergy*

within and between areas. In contrast, NBG exhibited sustained redundancy even when its MI was strong, indicating distinct computational roles rather than purely bandwidth differences."

2. The authors use data from two human subjects and one monkey. Results are variable between the two human subjects as well as the monkey data in certain cases. For example, the data shown in Figure 2B for BB response for gratings and noise/natural images do not allow a solid conclusion for the role of BB signals in carrying information about homogeneous images. In S1 the response is earlier for gratings and the one for noise follows, in S2 there is a higher initial response for noise and in the monkey there is a higher response for gratings. This seems to be a representative electrode in V1 which raises the question of what other electrodes look like. The authors do not comment on these. In S2, one might even detect a later sustained higher response for gratings in BB (something that is typical for NBG signals). A more nuanced discussion of these would help.

Response: We really appreciate the reviewer's comment, as it first allowed us to notice several mistakes in plotting **Figure 2**. These errors are because we uploaded an older version of the figure. In the current (and incorrect) **Figure 2**, the columns of SPCA, BB, NBG, and MI are flipped between Subject 1 and 2. Furthermore, the labeling for gratings and noise trials was also flipped for S1. We have now corrected both issues in the **new Figure 2**, which resolves some of the variability described by the reviewer:

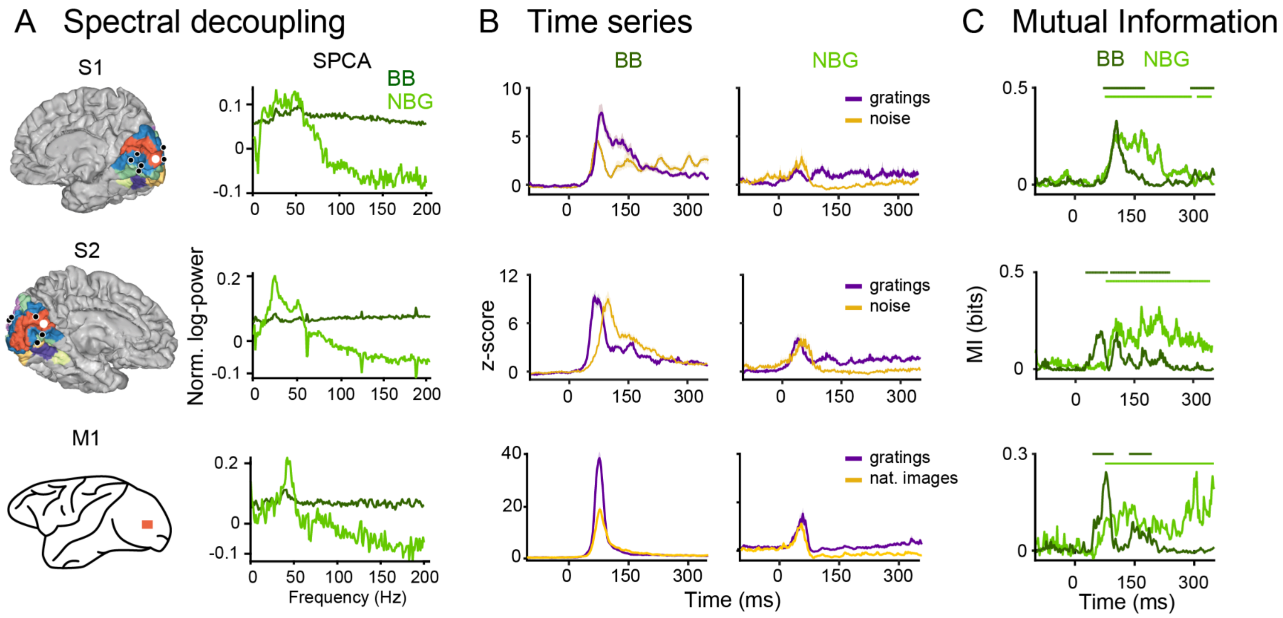

Figure 2. Spectral decoupling and mutual information analyses. (A) *Spectral decoupling from a V1 electrode in Subject 1 (S1) and Subject 2 (S2) (in white), and from a V1 electrode in Macaque 1 (M1) (in orange). The magnitude (i.e., the normalized log-power) of the first (dark green) and second (light green) spectral principal component (SPCA) is shown between 1 and 200 Hz. The first SPCA is the non-oscillatory, broadband (BB) component, and the second SPCA is the oscillatory, narrow-band gamma (NBG) component.* ***(B)*** *Time-series average across gratings (purple), and noise (yellow) trials, reconstructed with the BB (left panel) and NBG components (right panel) for S1, S2, and M1.* ***(C)*** *Mutual information analyses between gratings (dark green) and noise images (light green) for S1 and S2, and between gratings and natural images for M1. Mutual information (in bits) quantifies the effect size of the difference between the corresponding image categories, representing the amount of information encoded in the signals about the stimulus difference. The significant time points after a permutation test are shown as bars over the MI plots.*

Furthermore, to demonstrate consistency across electrodes and subjects, we have now plotted the SPCA with the raw traces and the MI differences for all electrodes and subjects in **Figure S3** and **Figure S4**. The figures and figure captions for the new figures now read like this:

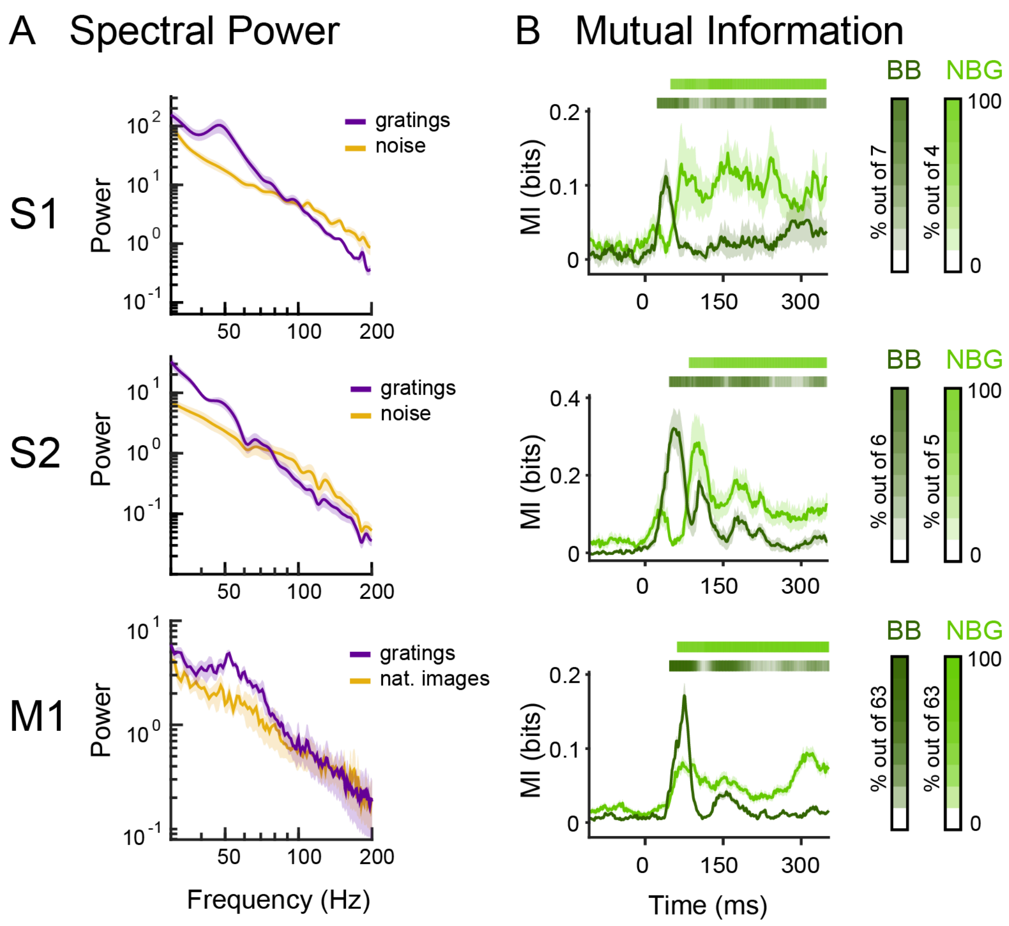

Figure S3. *Spectral power and mutual information for S1, S2, and M1.* ***(A)*** *Power spectral density for gratings (purple) and noise (yellow) stimuli for S1 and S2, and for gratings and natural images (yellow) for M1. Error bars represents the standard error of the mean (S.E.M) across electrodes (S1: V1,V2; S2: V1, V2, V3, and M1: V1).* ***(B)*** *Mutual information analyses between gratings and noise images for the BB component for S1 and S2 (dark green), and between gratings and natural images for M1 (dark green). Mutual information between gratings and noise images for the NBG component for S1 and S2 (light green), and between gratings and natural images for M1 (light green). The shaded horizontal bars represent significant MI values at the single-electrode level, with the colors representing the percentage of electrodes showing a significant effect per time point for BB (dark green) and NBG (light green). Error bars represent the S.E.M of the MI values across electrodes.*

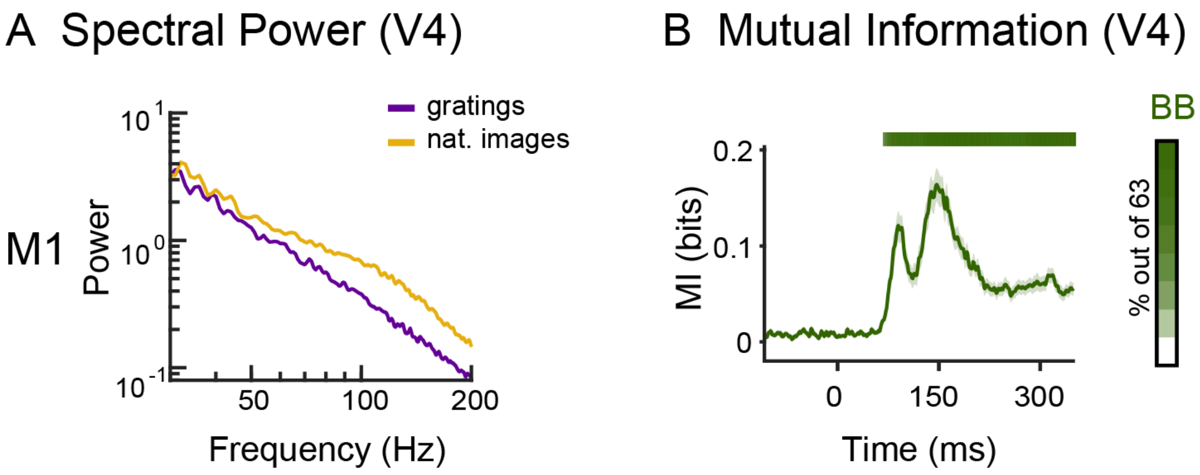

Figure S4. *Spectral power and mutual information for M1 in area V4.* ***(A)*** *Power spectral density for gratings (purple) and noise and natural images (yellow). Error bars represent the standard error of the mean (S.E.M) across electrodes in V4.* ***(B)*** *Mutual information analyses between gratings and natural images for M1 (dark green). The shaded horizontal bars represent significant MI values at the single-electrode level, with the colors representing the percentage of electrodes showing a significant effect per time point for BB (dark green). Error bars represent S.E.M of the MI values across electrodes.*

3. Previous work by the authors in human visual cortex (e.g., Hermes et al., 2015) and macaque V1 (e.g., Uran et al., 2022) has shown that gamma oscillations are induced by homogeneous, predictable stimuli (e.g., gratings), whereas unpredictable stimuli (e.g., complex natural scenes) evoke weak or no gamma oscillations. How do these previous results relate to the current findings presented in Figure 2B, C? Differences between gratings and noise, or between gratings and natural images, do not appear to emerge at the initial onset of narrowband gamma, but rather during the sustained phase. These effects appear less pronounced than the narrowband power spectra shown in Figure 1 of Hermes et al. (2015). This raises questions about the validity of the decoupling method. Is it possible that the method used to extract narrowband gamma in the current study takes into account a broader frequency range (30–80 Hz), whereas the actual effects may be confined to a narrower frequency band? At the very least, it would be useful to discuss how and why the current results may differ from those in previous studies.

Response: We thank the reviewer for relating our results to prior work showing strong gamma for homogeneous, predictable stimuli (gratings) and weak/absent gamma for unpredictable stimuli (complex natural scenes) (e.g., Hermes et al., 2015; Uran et al., 2022). In our figures, the narrowband-gamma (NBG) curves depict Mutual Information (MI), i.e., *category discriminability*, not raw gamma power. Early post-onset gamma transients can occur for both categories; only when responses diverge across categories show an MI increase, which naturally makes the sustained phase the main locus of MI differences in NBG (**Figure 2B,C**). Regarding extraction, our NBG component is not defined by an arbitrary wide band: it is the second spectral principal component with a peaked weight in the gamma range, and electrodes without a characteristic gamma peak were excluded from NBG analyses:

The NBG traces in **Figure 2B-C** plot MI, which indexes category-specific separation (gratings vs. noise images), not the absolute gamma envelope. Consequently, an initial gamma onset that is common to both categories contributes little to MI; category differences emerge during the sustained phase, when responses diverge. This is consistent with prior findings that gratings evoke strong, sustained gamma, whereas complex natural images often do not (Hermes et al., 2015;

Uran et al., 2022), while also explaining why our MI effect is temporally later than the initial gamma onset.

References:

- Hermes, D., Miller, K. J., Wandell, B. A., & Winawer, J. (2015). Stimulus dependence of gamma oscillations in human visual cortex. *Cerebral cortex*, *25*(9), 2951-2959.
- Uran, C., Peter, A., Lazar, A., Barnes, W., Klon-Lipok, J., Shapcott, K. A., ... & Vinck, M. (2022). Predictive coding of natural images by V1 firing rates and rhythmic synchronization. *Neuron*, *110*(7), 1240-1257.

Second, in the new **Figure S1**, to further visualize that the method used to extract narrowband gamma in the current study accounts for a range of 30–80 Hz, we have plotted SPCs for all electrodes, including the variance explained by the BB and NBG components. We have also plotted in the new **Figure S2**, a time-frequency reconstruction of SPC1 and SPC2 for stimuli that evoked most of the sustained gamma (gratings). We have also plotted the effect size of the difference between grating vs noise/natural images (i.e., the Mutual Information) in the time-frequency power to show the narrowband effects observed in the 30-80 Hz in S1, S2, and M1 (**Figure S5**).

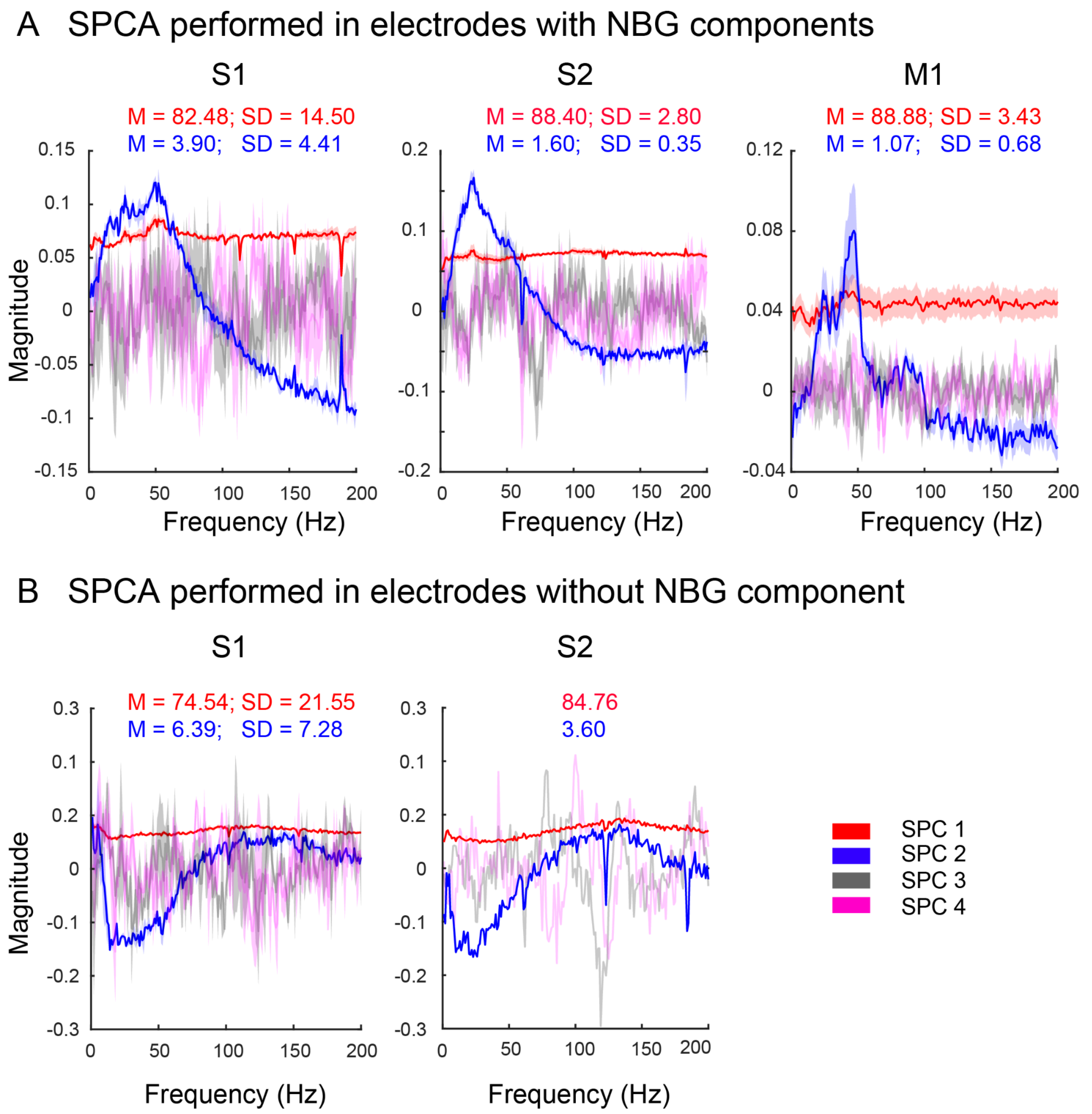

Figure S1. *SPCA component for S1, S2, and M1.* ***(A)*** *Averaged traces showing a characteristic NBG component for S1 (4 out of 7 electrodes), S2 (5 out of 6 electrodes), and M1 (63 out of 63*

electrodes). While PC1 (red) does not show a characteristic peak across frequencies (BB component), PCA 2 (blue) shows increased magnitude spanning the gamma band (NBG). The mean and standard deviation of the variance explained by the PC1 and PC2 components across channels are depicted in red and blue, respectively. Unlike PC1 and PC2, PC3 (gray) and PC4 (magenta) exhibit high channel variability without a characteristic peak. ***(B)*** *Averaged traces for electrodes without a characteristic NBG component for S1 (2 out of 7 electrodes), S2 (1 out of 6 electrodes). The mean and standard deviation of the variance explained by the PC1 and PC2 components across channels are depicted in red and blue, respectively. In contrast, PC1 shows a broadband component, and PC2 mainly peaks in the lower-frequency range (<10 Hz).*

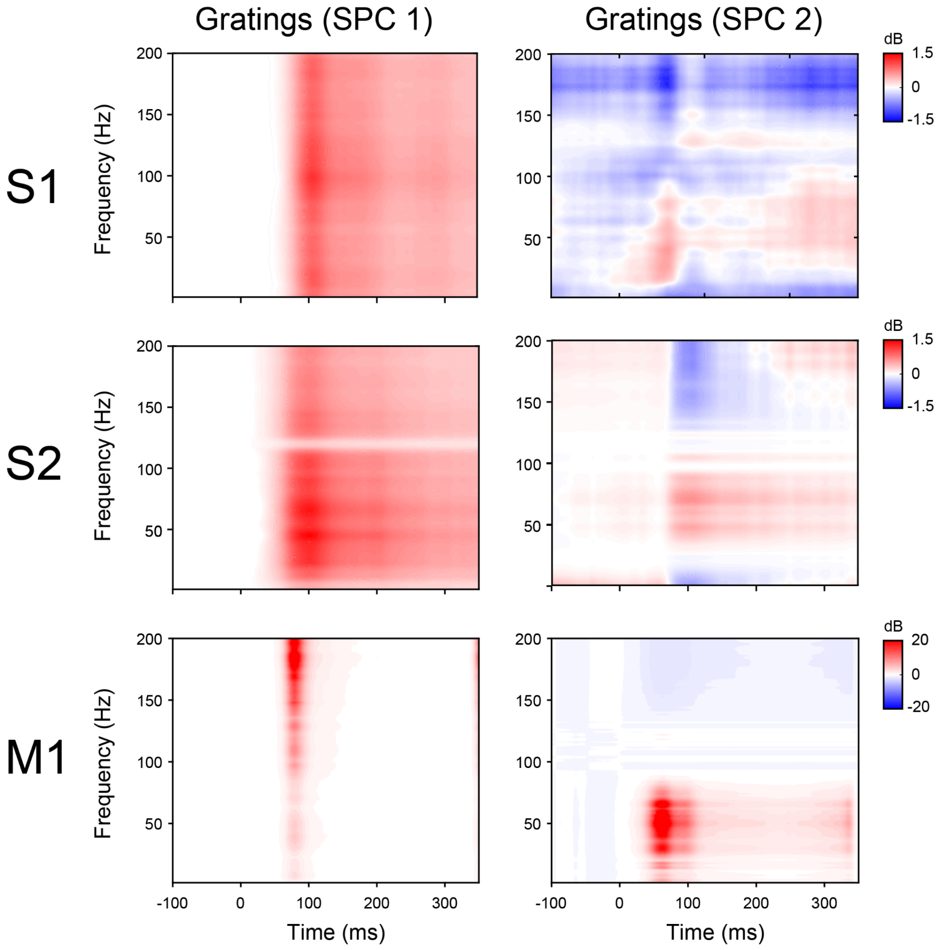

Figure S2. *Time–frequency spectral reconstruction using SPC1 and SPC2 in S1, S2, and M1. For each electrode and trial with grating stimuli, Welch power spectra were computed with a single full-length segment (nfft = 500), retaining positive-frequency bins up to 200 Hz. To capture spectral structure, spectra were log-normalized at each frequency, and spectral PCA (SPCA) was performed on the frequency–frequency covariance matrix. The first two components (SPC1 and SPC2) were treated as fixed spectral motifs. We then computed an SPC-specific time course: the signal was filtered with a bank of complex Morlet wavelets (5 cycles per frequency), the squared magnitude was taken to obtain band-power over time, convolution edge samples were discarded, each band's power trace was normalized to unit mean, a natural-log transform was applied, and the resulting log-power matrix was projected onto the SPC vector to yield a single time course for that component. To reconstruct absolute time–frequency (TF) power, we (1) paired this time course with the component's frequency profile to form a separable log-power map, (2) exponentiated that map to convert it into a multiplicative power modulation, and (3) multiplied, at each frequency, by the electrode mean Welch spectrum to return to absolute power units. We then baseline-normalized per frequency to −100–0 ms and reported the values in dB (10 log10 of the baseline ratio). Panels show TF maps averaged across channels. Power is expressed in decibels*

(dB) relative to the baseline period. While SPC1 spans most of the frequency axis, SPC2 shows non-zero power predominantly in the gamma range (30–80 Hz).

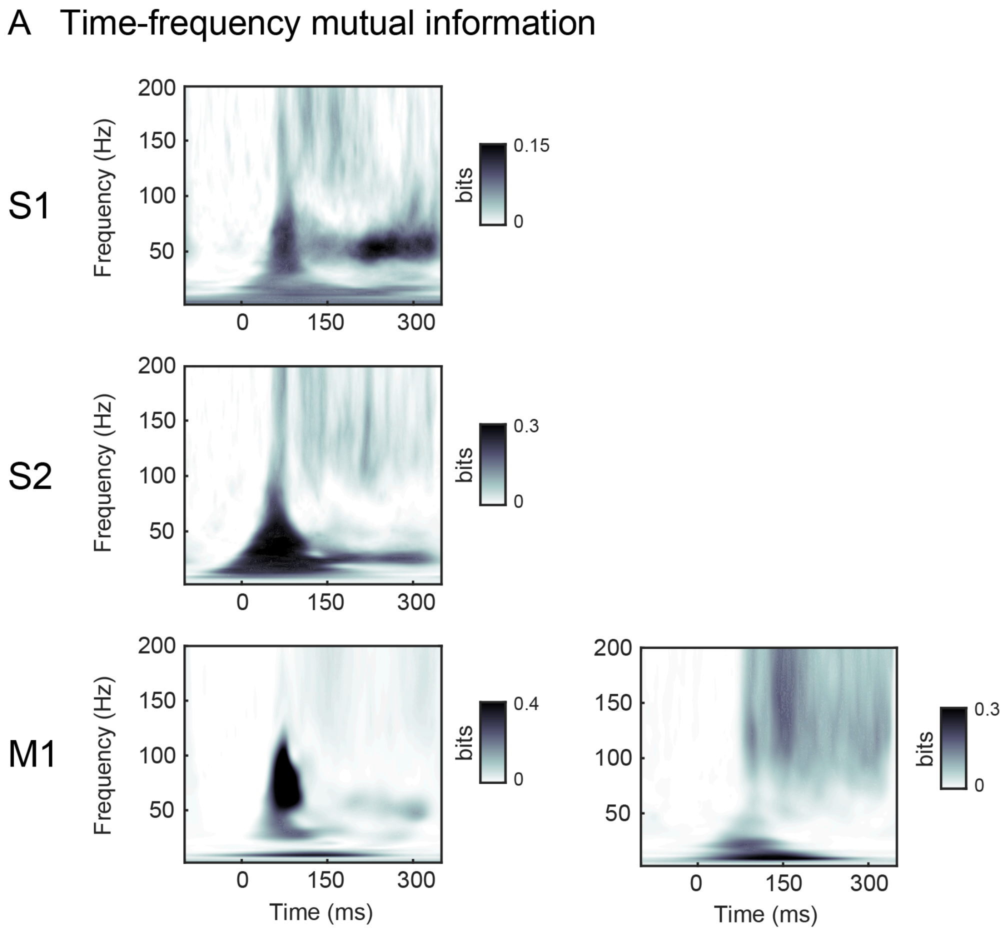

Figure S5. *Time-frequency mutual information for S1, S2, and M1. Mutual information (MI) between gratings and noise for S1 and S2, and between gratings and natural images for M1. MI was computed from power values across trials for each time-frequency bin from 1 to 200 Hz, within the -100 to 350 ms window around stimulus onset. MI represents the effect size (in bits) of the difference in spectral power for each corresponding stimulus comparison. Plots represent the average MI across electrodes (S1: 7 electrodes [V1 and V2 area]; S2: 6 electrodes [V1, V2 and V3 area], M1: 63 electrodes in V1 (left panel), and 63 electrodes in V4, right panel).*

4. In Figure 2C, the authors report that the onset of mutual information differs for BB and NBG signals, however, no statistical comparison of relative latencies is carried out. This should be provided in order to support the relevant claim.

Response: Thanks, we have now reported the exact onset of the significant MI effects based on the full results across electrodes anda datasets showin in the new supplementary **Figure S3**, showing the full results across electrodes and subjects.

__**Line 171-186:**__ *"We observed significant MI encoded in the BB (dark green) and NBG (light green) signals after contrasting gratings versus noise in S1 and S2, and gratings versus natural images in M1 (**Figure 2C**, and **Figure S3B**). Although both BB and NBG responses encoded significant information about the spatial homogeneity of images, we did observe some differences in latencies between the two types of dynamics (**Figure 2C**, and **Figure S3B**). Across electrodes and subjects (**Figure S3B**), the earliest significant MI effects appeared in the BB signals, while the MI effects in the NBG signals emerged later. Specifically, in S1, MI effects in the BB signals emerged as early*

*as 28 ms after stimulus onset compared to the 60 ms onset of the NBG signals (**Figure S3B;** upper panel**). Similarly, in S2, BB effects began at 48 ms while NBG effects began at 80 ms (**Figure S3B;** middle panel). In M1, BB effects began at 50 ms and NBG effects at 66 ms (**Figure S3B;** lower panel).”*

5. The authors show synergistic information for BB signals that appears to be transient in most cases. It is hard to conceive what this would reflect in terms of information processing and the relevant dynamics. It would be helpful to comment on that.

Response: We thank the reviewer for raising this important point. We have now expanded the Discussion to clarify how transient synergistic BB signals may relate to underlying computational dynamics, drawing on the transient vs sustained activity framework proposed by Vinck et al. (2025, TICS).

Lines 492-502: *“We speculate that early, largely transient BB synergy might reflect rapid perceptual inference supported by nonlinear, recurrent interactions across neural populations (Vinck et al., 2025). In our results, BB synergy typically emerges between early and later time points off-diagonally, consistent with the idea that a brief input-driven event pushes the system into a new multiareal attractor-like state, in which information about the stimulus is encoded in the joint activity pattern across nodes rather than at any single site. In such a regime, readout requires nonlinear integration of population activity (e.g., XOR-like computations), which is naturally captured by synergistic information.”*

References:

- Vinck, M., Uran, C., Dowdall, J. R., Rummell, B., & Canales-Johnson, A. (2025). Large-scale interactions in predictive processing: Oscillatory versus transient dynamics. Trends in Cognitive Sciences, 29(2), 133–148. https://doi.org/10.1016/j.tics.2024.09.013

6. When examining interactions between areas, the authors mention pairs of areas in which redundant or synergistic information was found. It would be extremely helpful to better appreciate this finding to provide a supplementary figure with examples of pairs of areas where no significant interactions were found. Although electrodes in human subjects are reported to cover several different areas it is not clear what the results looked like for pairs other than V1-V2/V3.

Response: In humans, data were limited to V1–V2 (for Subject S1) and V1–V2/V3 (for Subject S2), and all inter-areal comparisons showed significant effects. In macaque M1, on the other hand, inter-areal analyses were restricted to V1–V4 (**Figure 1A**). Accordingly, all inter-areal results pertain only to these recorded pairs. In M1, however, there were non-significant pairs. A representative non-significant example of co-I between V1-V4 pairs is shown below:

M1 (BB response): co-I between electrode 10 and 41:

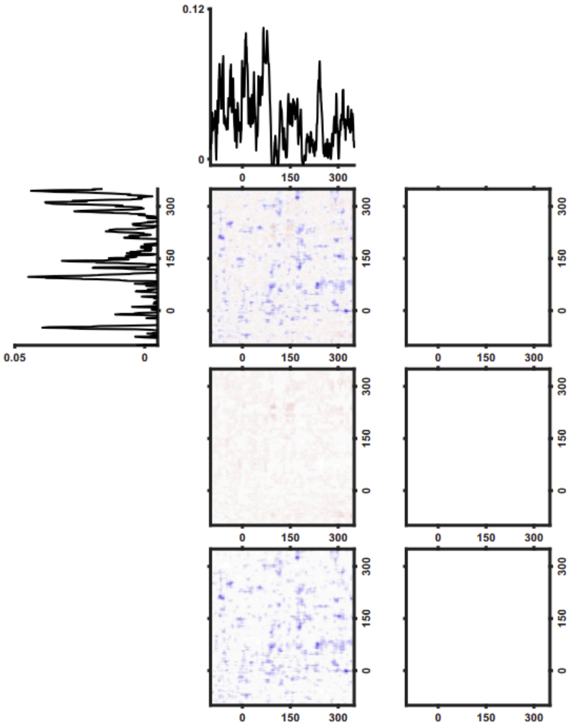

7. Previous studies have reported that interareal functional connectivity (e.g. spike-LFP coherence) is stronger for sites with overlapping RFs. Did the authors examine whether and how RF location of different sites influences co-information? This is critical to understand the potential role of distinct neuronal dynamics in a distributed network. If the sites sampled did not have overlapping RFs, this should be discussed and mentioned as a potential limitation.

Response: To understand the degree to which different electrodes had overlapping population receptive fields (pRFs), we plot the pRFs calculated using the image-derived encoding model from the original study (**Figure S6**) (Hermes et al., 2019). This figure shows that electrodes in both subjects had pRFs that overlapped to different degrees. For example, in subject 1, pRFs were all in the upper left quadrant and varied in degrees of overlap. In subject 2, the first 4 electrodes had overlapping pRFs in the upper right quadrant, and the other two electrodes had overlapping pRFs in the lower right quadrant. Therefore, in each subject, there were some electrodes with overlapping pRFs, we now report this in the methods:

Line 625-632: *"In each human subject, some electrodes showed overlapping population receptive fields. For each electrode, the location (x,y) and size (sigma) of the population receptive field (pRF) were taken from the previous calculation of a second-order contrast (SOC) model (Hermes et al., 2019), representing the location of the image to which the electrode responds. The pRFs of each electrode used in S1 and S2 can be found in* ***Figure S6****"*

References:

- Hermes, D., Petridou, N., Kay, K. N., & Winawer, J. (2019). An image-computable model for the stimulus selectivity of gamma oscillations. *eLife, 8*, e47035. https://doi.org/10.7554/eLife.47035

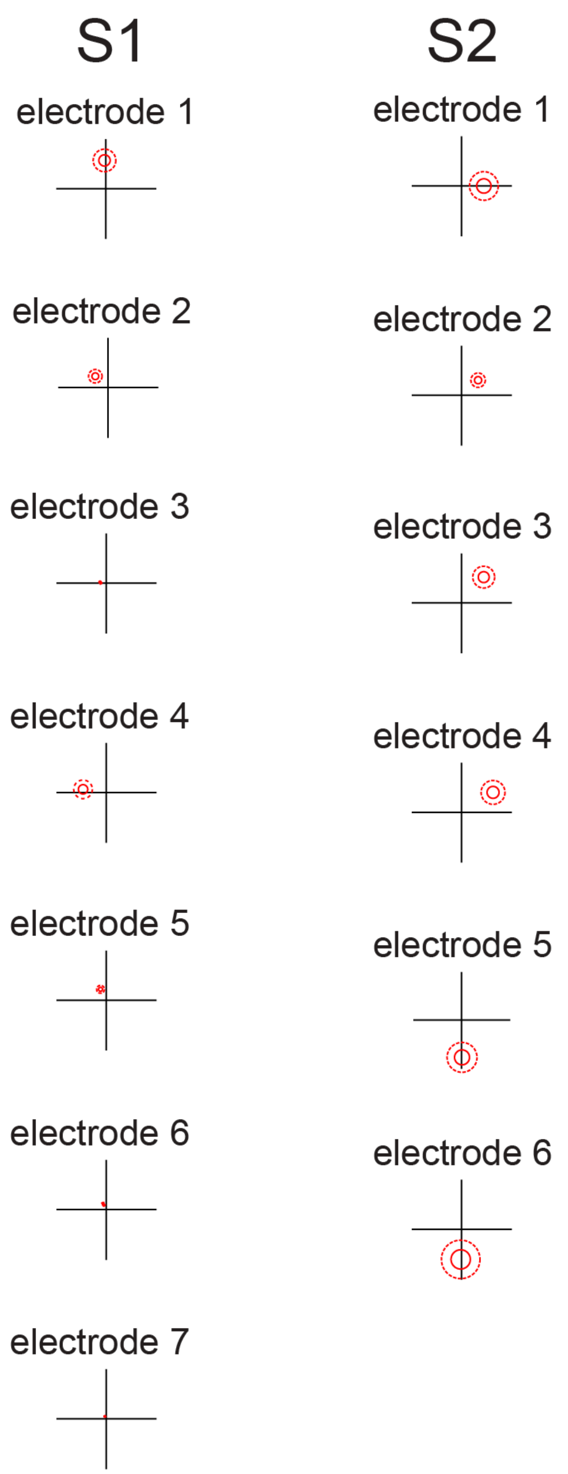

Figure S6. *Location of population receptive fields (pRF) for S1 and S2. The population receptive field for each electrode was defined by a Gaussian, indicated by the 1- and 2-sd contours (solid and dotted red lines.*

8. The study did not find narrowband gamma in macaque V4, although gamma power in V4 has been found in previous studies even if this is weaker than in V1. This difference should be stated and some speculation on that should be provided to avoid confusion. Moreover, the fact that NBG was not found in V4 did not allow the authors to confirm the finding that inter-areal interactions as reflected on NBG activity carry redundant information. This should be acknowledged.

Response: Thank you for the opportunity to explicitly report our findings on V4 and the literature discussing NBG in V4. We note that studies investigating gamma in V4 (and V1) usually optimize

stimuli to induce narrow-band gamma. However, this was not done in the present macaque dataset. We note that several studies/datasets report V1 gamma, but not V4 gamma (e.g., Debes and Dragoi, 2023; Spyropoulos et al., 2024). This also includes data with grating stimuli (Spyropoulos et al., 2024). In other studies, V4 gamma was reported (Grothe et al., 2012; Vinck et al., 2013), although it is noted that the V4 gamma rhythm was reported to be substantially weaker than in area V1 (e.g., spike-field locking values are around 0.003 in area V4 but around 0.1 in area V1, Vinck et al., 2013; Onorato et al., 2020). The stimulus and the recording type both play essential roles. We also note that data from the Fries lab with simultaneous V1-V4 ECoG recordings and natural images does not reveal V4 gamma (Jarrod Dowdall, 2023, PhD thesis, Radboud Repository). Due to the lack of NBG in V4, as now shown in the new **Figure S4,** it was not possible to quantify redundant information between V1 and V4 in the macaque dataset.

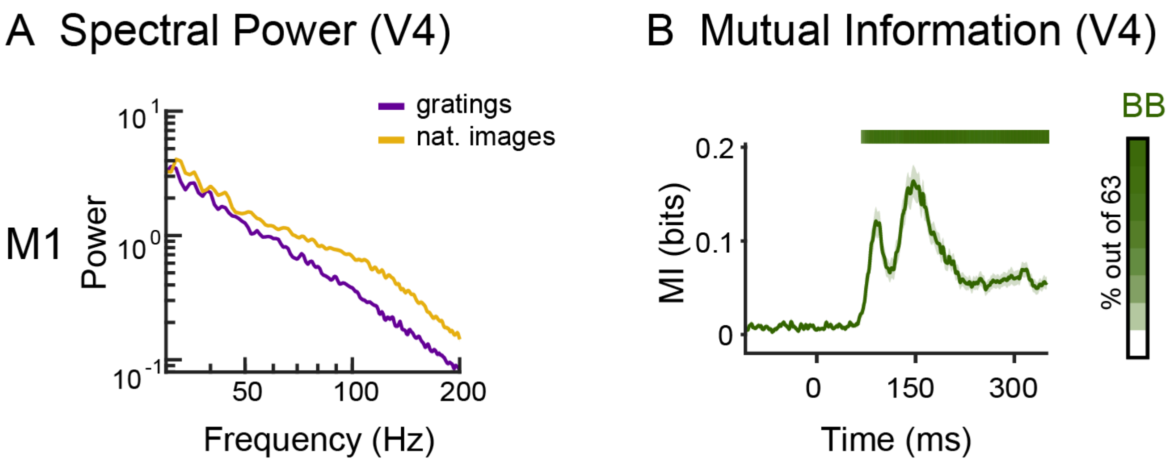

Figure S4. *Spectral power and mutual information for M1 in area V4.* ***(A)*** *Power spectral density for gratings (purple) and noise and natural images (yellow). Error bars represent the standard error of the mean (S.E.M) across electrodes in V4.* ***(B)*** *Mutual information analyses between gratings and natural images for M1 (dark green). Error bars represent S.E.M across electrodes. The shaded horizontal bars represent significant MI values at the single-electrode level, with the colors representing the percentage of electrodes showing a significant effect per time point for BB (dark green).*

Finally, to further show the reviewer the absence of NBG differences between gratings and natural images in V4, we have now also computed the MI between stimulus categories in the full time-frequency chart (i.e., the effect size of the difference in spectral power between gratings and natural images) in **Figure S5.**

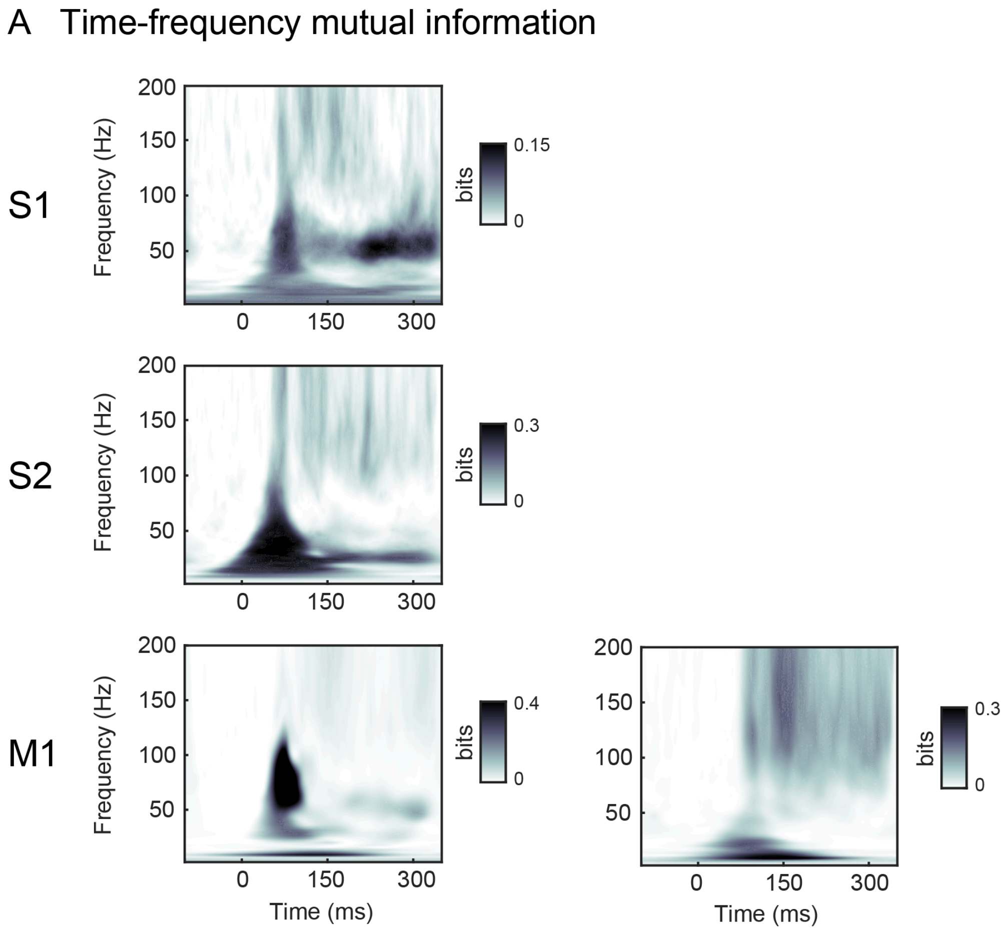

Figure S5. *Time-frequency mutual information for S1, S2, and M1. Mutual information (MI) between gratings and noise for S1 and S2, and between gratings and natural images for M1. MI was computed from power values across trials for each time-frequency bin from 1 to 200 Hz, within the -100 to 350 ms window around stimulus onset. MI represents the effect size (in bits) of the difference in spectral power for each corresponding stimulus comparison. Plots represent the average MI across electrodes (S1: 7 electrodes [V1 and V2 area]; S2: 6 electrodes [V1, V2 and V3 area], M1: 63 electrodes in V1 (left panel), and 63 electrodes in V4, right panel).*

References:

- Debes SR., Dragoi V (2023). Suppressing feedback signals to visual cortex abolishes attentional modulation.*Science* 379,468-473.
- Spyropoulos, G., Schneider, M., van Kempen, J., Gieselmann, M.A., Thiele, A., Vinck, M (2024). Distinct feedforward and feedback pathways for cell-type specific attention effects. *Neuron* 112, 2423-2434.
- Grothe I., Neitzel SD., Mandon S., Kreiter AK (2012). Switching Neuronal Inputs by Differential Modulations of Gamma-Band Phase-Coherence. *Journal of Neuroscience* 32, 16172-16180.
- Vinck M., Womelsforf T., Buffalo EA., Desimone R., Fries P (2013). Attentional Modulation of Cell-Class-Specific Gamma-Band Synchronization in Awake Monkey Area V4. *Neuron* 80, 1077-1089.

Minor comments:

Computing a measure of image homogeneity:

1. Abbreviations S1 and S2 for human subjects 1 and 2, and M1 for the macaque are used before being defined in the main text.

Response: Thanks. This has now been corrected and defined at the end of the introduction:

Line 69-73: *"Here, we analyzed electrocorticography (ECoG) and local field potentials (LFP) signals in the visual cortex of humans (subjects S1 and S2) and non-human primates (macaque M1) and decomposed signals into broadband and narrowband activity."*

2. "gratings had the highest image homogeneity () and noise () patterns had the lowest image homogeneity". Maybe move the word patterns before the parenthesis.

Response: Thanks. For clarity and readability, we have now moved all stimulus descriptive statistics to the Methods section.

3. Including an explanation in the Methods section of what the degrees of freedom in the t-tests correspond to would improve clarity.

Response: Thanks. We have now included these descriptions in the Methods section.

Decoupling broadband and oscillatory responses from the ECoG and LFP signals
4. Please rephrase: "In each electrodes and datasets".

Response: Thanks. Done.

5. When discussing the decoupling of signals, the authors refer to Figure 1D, when they should be referring to Figure 1C.

Response: Thanks. Fixed.

6. Figure 2A shows some noticeable noise peaks for S1. Would notch filtering these peaks affect the results?

Response: We used a notch filter for S1 to remove line noise energy (now plotted as S2 due to a mistake in the Figure, see **point 2** of the reviewer). This can also be seen in the PSD for this subject plotted in Supplementary **Figure S3**. It stands to reason that **not** applying notch filtering might introduce distortions that can affect the results, but there is no reason to suspect that applying notch filtering to remove an artifact would distort the results.

Temporal redundancy in BB and NBG signals

7. A brief overview of co-information as an extension of mutual information to more than two variables in the main text would help the reader follow the subsequent analyses more easily.

Response: We thank the reviewer for the possibility to clarify the co-information analysis, as they are a vital part of the analytical methods and the results. We have added a brief description in the Results section:

Line 194-198: *"Co-I tests whether two neural responses carry the same information (redundant) or if the relationship between them carries extra (synergistic) information about the stimulus: positive values indicate redundancy; negative values indicate synergy (see Methods and Figure 1D)"*

8. Are the diagonal vs. square patterns observed here related to those found in cross-temporal decoding analyses (e.g., Meyers et al., 2008)? Making this connection would help readers who are more familiar with decoding approaches better follow the results.

Response: Yes, they are. However, it is worth noting that while more traditional decoding approaches such as Meyers et al. (2008) or King & Dehaene (2014) capture only how the redundant information encoding evolves over time, our approach also captures how synergistic representations evolve across time. The redundant patterns here should correspond to evolving information representations captured by cross-temporal decoding analyses, and the relationship between co-I patterns (square vs diagonal) and time-generalization patterns resulting from MVPA decoding has been explicit in the Results section:

Lines 203-235: "*Regarding the dynamics of redundant information within BB and NBG signals, we observed different patterns of temporal redundancy for the two signals. Redundancy quantifies the degree to which two time points carry the same trial-by-trial information about the stimulus category (i.e., high vs. low homogeneity).*

This is conceptually similar to the temporal generalisation method (King et al., 2014), which quantifies the similarity of a multivariate predictive response pattern over time. Similarly, different temporal patterns of redundancy can reveal different signatures of information representation. A diagonal pattern indicates what King et al. (2014) term a chain, in which a sequence of discrete generators represents the stimulus over time. In contrast, a square pattern indicates a sustained response, which is more likely to result from a single process maintained over time.

*In the case of redundancy, a diagonal pattern indicates a continually evolving representation of information, distributed over time. In such a system, a downstream readout would benefit from integrating the signal over time, as later time points continue to provide new information. In the BB signals, diagonal redundancy was observed in S1 and S2 (**Figure 3A, D**) and M1 (**Figure 5A**).*

*Instead, a sustained block pattern was observed in the NBG signals. Redundancy over a large temporal region suggests a unitary information processing event in which the same information is obtained, whichever time point you sample from. In this case, a downstream processor would only need to observe one time point to get all the information, although integration might still have benefited from an SNR perspective. In the NBG signals, we observed sustained redundancy over time across the entire period after stimuli presentation in S1 and S2 (**Figure 3B, E**); and M1 (**Figure 5B**).*"

9. There are several figure-related issues: (a) Top panels of Figure 3 display green pixels that are not indicated in the color bar; (b) the error bar for V1 MI in the top panel of Figure 4D appears misaligned; (c) Figure 6 probably lacks an error bar for V1 MI and (d) the reference to Figure 3A,C for diagonal redundancy in BB signals of S1 and S2 should probably be Figure 3A,D.

Response: Thanks. All of these issues have now been corrected. The alleged green dots are not really there, as the colorbar spans from blue to white to red without any green. However, when zooming in close, the pixelation may give an impression of colour disturbance. This is due to the sampling rate of the co-information analysis, but does not reflect any errors in plotting/analysis.

10. In Figures 3C and 3F, does the y-axis represent 0.4% or 40% of significant time points? If it is 40%, please correct the axis or label accordingly. Same applies to Figures 4 and 5.

Response: Thanks for noticing this mistake. Our quantification actually refers to the "proportion" of significant points and not the percentage "%". We have now corrected this in all figures.

Spatiotemporal redundancy in BB and NBG signals
The reference to specific panels of Figure 4 in the second paragraph should be corrected.

Response: Thanks. It has now been corrected.

Spatiotemporal synergy in BB and NBG signals
11. The text currently reads: “In contrast, and consistent with the findings within regions, interareal synergistic information was mainly observed in the BB signals in S1 and S2 (Figure 4A,C)”. Should the reference be to panels A and B instead of A and C? This is repeated in the following sentences.

Response: Indeed. This has now been corrected.

Methods
Macaque recordings

12. It should be justified why previous related studies by some of the authors (e.g., Uran et al., 2022) used data from three monkeys whereas the current study reports data from only one animal. If the third animal in the Uran et al., 2022 study is the same as the one included here, it appears that it was implanted with Utah arrays. This needs to be stated.

Response: Thank you. We now clarify that the macaque dataset comprises sessions from a single animal (Monkey H), selected because they included both a grating condition and natural images required for our image-homogeneity contrast, and passed all quality controls. Uran et al. (2022) reported results across three animals for broader questions; here, we reuse only the subset of sessions from Monkey H that match our design and preprocessing. The other monkeys were implanted with semi-chronic microelectrode array Microdrives (SC32-1, Gray Matter Research) and only covered a smaller subset of stimuli per unit. We also state explicitly that recordings were obtained with chronically implanted microelectrode arrays in the visual cortex (as in Uran et al., 2022). This is now specified in *Methods/Macaque recordings*:

Line 636-640: “*The animal was implanted with a Utah array with 64 microelectrodes (inter-electrode distance 400~μm, half of them with a length of 1 mm and half with a length of 0.6 mm, Blackrock Microsystems), and inserted a reference wire under the dura towards the parietal cortex.”*

13. The text reads “Monkeys self-initiated trials”. It should be “monkey” instead.
Stimuli and task

Response: Corrected to *“Monkey self-initiated trials.”*

14. The acronym LPIPS is not defined.

Response: We now define LPIPS (Learned Perceptual Image Patch Similarity) at first use and cite it as the metric used to select the least-predictable natural image per channel for the macaque contrast.

Separating BB from NBG signals

The text reads: “3 electrodes (S1: V3 ventral; S2: V2 ventral, V2 ventral”. The closing parenthesis is missing and “V2 ventral” is repeated.

Response: Thanks. This section has been rephrased.

………..

Reviewer #4 (Remarks to the Author):

I co-reviewed this manuscript with one of the reviewers who provided the listed reports. This is part of the Nature Communications initiative to facilitate training in peer review and to provide appropriate recognition for Early Career Researchers who co-review manuscripts.

Reviewer #5 (Remarks to the Author):

This manuscript presents an intriguing and conceptually rich investigation into the distinct roles of narrowband gamma (NBG) oscillations and broadband (BB) signals in encoding and transmitting visual information. The authors leverage information-theoretic measures—redundancy and synergy—to probe how these signals differentially contribute to visual processing, with the hypothesis that NBG reflects feedforward sensory input and network coordination, while BB may index nonlinear dynamics supporting recurrent, modulatory feedback. Using ECoG and LFP recordings from nonhuman primates viewing homogenous (gratings) and heterogeneous (naturalistic or noise) images, the study applies spectral PCA to dissociate NBG and BB signals and uses DNN-derived predictability scores to quantify image homogeneity. This combination of signal decomposition and information-theoretic analysis provides a promising framework for disentangling the functional contributions of different neural signals. That said, there are some inconsistencies in the exposition and rationale that reduce clarity. In several cases, key concepts, figure captions, or methodological choices required inference on the part of the reader. I also have concerns about the limitations of the methods that are not sufficiently addressed. With clearer justification and validation for some of the analytical decisions, the manuscript would be substantially strengthened.

Response: We thank the reviewer very much for finding the manuscript intriguing and conceptually rich, and for pointing out inconsistencies in the paper. The following comments will address these inconsistencies and further clarify any methodological or theoretical issues.

1. The report contains no assurances of ethical oversight or approval for research with human or animal subjects.

Response: We thank the reviewer for pointing out this deficit. No new data were collected for this study; all analyses were performed on data from previous studies. However, we have added information regarding ethical care and informed consent in the Methods section (“Data acquisition”):

Lines 610-614: *“Subjects gave informed consent and the study was approved by the Stanford University IRB and the ethics committee at the University Medical Center Utrecht in accordance with the 2013 provisions of the Declaration of Helsinki.”*

Lines 655-657: *“All procedures complied with the German and European regulations for the protection of animals and were approved by the regional authority.”*

2. The use of spatial homogeneity as a stimulus dimension for manipulation is not motivated in the report. Why is this important, interesting, ethological, etc.?

Response: We apologize for not explaining this point clearly enough. We first want to clarify an important point: the central topic of the present study is not the stimulus correlates of NGB - unfortunately, the beginning of the results section could have given this impression.

In fact, the stimulus correlates of NGB have been extensively studied in much previous work. This prior work (which is further supported by the present study) has highlighted that a key factor to predict NGB is the structural predictability of local image patches given the surround, i.e. the extent to which the image in the surround predicts the center, which relates to the homogeneity of orientation across space (Uran et al., 2022, Hermes et al., 2019, Vinck & Bosman, 2016, Ray paper, 2022, Coen-Cagli et al., 2012). This was extensively controlled for other image factors like luminance contrast, firing rates, spatial frequency, etc. in Uran et al. (2022), who showed that among many image features, structural predictability is the best predictor of NGB.

In this study, we stick to the neutral descriptor **homogeneity**, which we define in the Results (see below).

Importantly, the key topic of the present study is the information redundancy and information synergy in BB and NGB signals. Studying information redundancy and synergy requires examining a stimulus dimension for which NGB contains information. This motivated us to examine stimulus homogeneity, enabling us to further investigate redundancy and synergy and to compare them with BB signals.

To make this more apparent to the reader, we have revised the start of the Results section:

Lines 83-108: *"We focused on a feature of images that is known to be reliably encoded by NGB in both humans and macaques, namely the structural homogeneity of images (Hermes et al., 2019; Shirhatti et al., 2022; Uran et al., 2022; Vinck and Bosman, 2016). We operationalize homogeneity as the extent to which the image in the surround predicts the precise structure of receptive field inputs in the center (Uran et al., 2022), which relates to the homogeneity of structural features such as orientations across space (Coen-Cagli et al., 2012; Hermes et al., 2019). Uran et al. (2022) have shown that, among many image features, structural predictability is the best predictor of NGB. This approach allowed us to compare the nature of information representations between BB and NBG in terms of synergy and redundancy.*

In both humans, we analyzed a previously reported dataset of bandpass-filtered grayscale images that varied along several parametric dimensions. We compared gratings to a set of images with low spatial predictability. Using a deep neural network (DNN) to predict the visual input in the center from the surround (similar to Uran et al., 2022), we verified that gratings had higher homogeneity than noise images (S1: $p < 0.001$; S2: $p = 0.002$: see methods). In macaques, we analyzed gratings and grayscale natural images previously reported in macaques by Uran et al. (2022). We compared gratings to a set of natural images that had low homogeneity, as identified with the DNN (difference gratings and natural images: $p < 0.001$: see methods)."

3. The use of the term "homogeneity" by the authors seems to conflict with my intuitive understanding of the term. For example, generally pure "noise" I think would be considered more

homogenous than a grating. Sure, it's harder to predict the exact pixel values given the surround, but the statistical *texture* of the image is more spatially uniform.

Response: Thanks for the comment. We understand the reviewer's point. In the literature, studies have used both the terms homogeneity (Coen-Cagli et al., 2012; Hermes et al., 2019) and predictability (Vinck & Bosman, 2016; Uran et al., 2022). The advantage of the term homogeneity is that it avoids explicit associations with the predictive coding literature, which may be perceived as misleading; however, it can be confusing, as a noisy texture can be homogeneous. The homogeneity referred to earlier pertained to stimulus orientation features across space; this homogeneity relates to structural predictability, i.e., the extent to which the surround can predict the center.

To avoid these confusions and debates, we have simply defined homogeneity for this study at the beginning of the results, relating these two perspectives explicitly:

Lines 87-92: *"We operationalize homogeneity as the extent to which the image in the surround predicts the precise structure of receptive field inputs in the center (Uran et al., 2022), which relates to the homogeneity of structural features such as orientations across space (Coen-Cagli et al., 2012; Hermes et al., 2019)."*

References:

- Coen-Cagli, R., Dayan, P., & Schwartz, O. (2012). Cortical surround interactions and perceptual salience via natural scene statistics. *PLOS Computational Biology, 8*(3), e1002405. https://doi.org/10.1371/journal.pcbi.1002405
- Hermes D., Petridou N., Kay KN., Winawer J (2019) An image-computable model for the stimulus selectivity of gamma oscillations, eLife 8:e47035.
- Uran, C., Peter, A., Lazar, A., Barnes, W., Klon-Lipok, J., Shapcott, K. A., ... & Vinck, M. (2022). Predictive coding of natural images by V1 firing rates and rhythmic synchronization. *Neuron*, *110*(7), 1240-1257.
- Vinck, M., & Bosman, C. A. (2016). More gamma more predictions: Gamma-synchronization as a key mechanism for efficient integration of classical receptive field inputs with surround predictions. *Frontiers in Systems Neuroscience, 10*, 35.

4. It is not apparent to me why a DNN should be expected to complete the center of an image in a way that is texturally homogenous with its surround. For example, if given a picture of a sunflower in which the central disk has been masked leaving only the surrounding ray petals, my impression is that the DNN should not fill in the center with more yellow petals, but rather complete the image as a sunflower with a dark central disk. But this image would not be spatially homogenous.

Response: Please check our response to point 3.

5. Was the image model trained on images of gratings and noise patterns? The relative image completion performance for different classes of images might be explained not by differences intrinsic to the test images, but rather to the training history of the DNN. Can the authors rule this in/out? Rather than actually tapping into image homogeneity, might the authors just be tapping into how familiar the test images are to the DNN, for reasons not due to their image statistics but rather due only to the decisions of the people that trained the model?

Response. Thanks for the comment. These networks were trained on natural images. Evidently, such networks will extrapolate structure from the surround to the center (filling in), which is possible for gratings but not for noise patterns. The loss function then is the similarity of the filled-in patch to the ground-truth patch (extensively described in Uran et al., 2022, Neuron). The confusion here stems from the terminology "homogeneity," which suggests a noise image is also homogeneous.

Moreover, we emphasize that the purpose of our study, as explained in point 2 of reviewer 5, is to study information structure, not stimulus correlates of NBG.

Reference:

- Uran, C., Peter, A., Lazar, A., Barnes, W., Klon-Lipok, J., Shapcott, K. A., ... & Vinck, M. (2022). Predictive coding of natural images by V1 firing rates and rhythmic synchronization. *Neuron*, *110*(7), 1240-1257.

6. The authors report "As expected, gratings had the highest image homogeneity (S1: xbar = 0.171…)...". What is x-bar? What are the units? Can any context be given for these numbers? It looks like maybe lower numbers imply greater homogeneity? Are these values bounded in some way? These numbers are completely uninterpretable as reported. Actually, now I'm really confused, because for the human subjects the authors report "x-bar" values around 0.17 for gratings, and claim that those images have greater homogeneity than noise, which had "x-bar" values of around 0.3 (implying an inverse relationship between x-bar and homogeneity); however, for the monkey subject, the authors report "predictability" (which I think they're using interchangeably with "homogeneity", but it would be better to just pick one term and stick with it consistently) of x-bar around 0.24 for gratings and say that implies greater homogeneity than natural images with an x-bar of 0.009, which implies a direct relationship between x-bar and homogeneity. So, I really cannot make heads nor tails of this.

Response: We thank the reviewer for their comment. We first want to clarify an important point: the central topic of the present study is not the stimulus correlates of NGB - unfortunately, the beginning of the results section could have given this impression.

The stimulus correlates of NGB have been extensively studied in much previous work. This prior work (which is further supported by the present study) has highlighted that a key factor to predict NGB is the structural predictability of local image patches given the surround, i.e. the extent to which the image in the surround predicts the center, which relates to the homogeneity of orientation across space (Uran et al., 2022, Hermes et al., 2019, Vinck & Bosman, 2016, Ray paper, 2022, Coen-Cagli et al., 2012). This was extensively controlled for other image factors, such as luminance contrast, firing rates, spatial frequency, etc., in Uran et al. (2022), who showed that, among many image features, structural predictability is the best predictor of NGB.

In this study, we stick to the neutral descriptor **homogeneity**, which we define in the Results (see below).

Importantly, the key topic of the present study is the information redundancy and information synergy in BB and NGB signals. Studying information redundancy and synergy requires examining a stimulus dimension for which NGB contains information. This motivated us to examine stimulus homogeneity, enabling us to further investigate redundancy and synergy and to compare them with BB signals.

To make this more apparent to the reader, we have revised the start of the Results section:

Lines 87-108: *"We focused on a feature of images that is known to be reliably encoded by NGB in both humans and macaques, namely the structural homogeneity of images (Hermes et al., 2019; Shirhatti et al., 2022; Uran et al., 2022; Vinck and Bosman, 2016). We operationalize homogeneity as the extent to which the image in the surround predicts the precise structure of receptive field inputs in the center (Uran et al., 2022), which relates to the homogeneity of structural features such as orientations across space (Coen-Cagli et al., 2012; Hermes et al., 2019). Uran et al. (2022)*

have shown that, among many image features, structural predictability is the best predictor of NGB. This approach allowed us to compare the nature of information representations between BB and NBG in terms of synergy and redundancy.

In both humans, we analyzed a previously reported dataset of bandpass-filtered grayscale images that varied along several parametric dimensions. We compared gratings to a set of images with low spatial homogeneity. Using a deep neural network (DNN) to predict the visual input in the center from the surround (similar to Uran et al., 2022), we verified that gratings had higher homogeneity than noise images (S1: $p < 0.001$; S2: $p = 0.002$; see Methods). In macaques, we analyzed gratings and grayscale natural images previously reported in macaques by Uran et al. (2022). We compared gratings to a set of natural images that had low homogeneity, as identified with the DNN (difference gratings and natural images: $p < 0.001$; see Methods)."

And in the Methods section:

Lines 690-719: *"LPIPS (Learned Perceptual Image Patch Similarity) predictability scores measure how different the predicted center of an image is from its true center, based on the surrounding context. Low scores indicate that the prediction is similar to the true center. High scores mean the prediction is dissimilar to the true center. Hence, according to our definition of image homogeneity, low scores indicate high homogeneity, while high scores indicate low homogeneity.*

Thus, to identify images with the highest and lowest homogeneity in human subjects S1 and S2, we first ranked all images by their predictability scores. Oriented grating images (80 images) showed the lowest scores (i.e., high homogeneity). Noise images (80 images) showed the highest scores (i.e., low homogeneity). We then selected these two image categories for further analysis. As a result, the MI and co-I analyses were performed on 80 gratings and 80 noise trials per subject. To confirm that the predictability scores resulted in significant differences between categories, we used a two-sided t-test to compare the scores of the pre-selected images. Gratings showed the highest image homogeneity (i.e., lowest scores) in S1 (mean = 0.171; SD = 0.081) and S2 (mean = 0.179; SD = 0.082), and noise showed the lowest homogeneity (i.e., highest scores) in S1 (mean = 0.343; SD = 0.304) and S2 (mean = 0.307; SD = 0.274) (Figure 1B). As expected, we observed higher predictability for gratings compared to noise images across images and electrodes in S1 ($t(1,110) = 4.101$, $p < 0.001$) and in S2 ($t(1,94) = 3.08$, $p = 0.002$).

Unlike the human participants, in the original study by Uran et al. (2022), the macaque M1 was shown a set of natural images in a total of 20 sessions. In each of the first four recording sessions, M1 was also shown a single oriented grating. There were 20 trials per image. To identify images with the highest and lowest homogeneity in M1, we used LPIPS to determine the highest and lowest predictable natural stimuli in each session. This resulted in 80 gratings (20 images x 4 sessions = 80 images) and 80 natural-image trails (4 images with the lowest scores per session x 20 sessions = 80 images) being selected for further MI and co-I analyses. When comparing the predictability scores, we observed higher homogeneity for gratings (mean = 0.009; SD = 0.013) than for natural images (mean = 0.244; SD = 0.146) across images and electrodes ($t(1,470) = 24.45$, $p < 0.001$)."

References:

- Hermes, D., Petridou, N., Kay, K. N., & Winawer, J. (2019). An image-computable model for the stimulus selectivity of gamma oscillations. *eLife, 8*, e47035. https://doi.org/10.7554/eLife.47035
- Shirhatti, V., Ravishankar, P., & Ray, S. (2022). Gamma oscillations in primate primary visual cortex are severely attenuated by small stimulus discontinuities. *PLOS Biology, 20*(6), e3001666. https://doi.org/10.1371/journal.pbio.3001666

- Uran, C., Peter, A., Lazar, A., Barnes, W., Klon-Lipok, J., Shapcott, K. A., Roese, R., Fries, P., Singer, W., & Vinck, M. (2022). Predictive coding of natural images by V1 firing rates and rhythmic synchronization. *Neuron, 110*(7), 1240–1257.e8. https://doi.org/10.1016/j.neuron.2022.01.002
- Vinck, M., & Bosman, C. A. (2016). More gamma more predictions: Gamma-synchronization as a key mechanism for efficient integration of classical receptive field inputs with surround predictions. *Frontiers in Systems Neuroscience, 10*, 35. https://doi.org/10.3389/fnsys.2016.00035
- Coen-Cagli, R., Dayan, P., & Schwartz, O. (2012). Cortical surround interactions and perceptual salience via natural scene statistics. *PLOS Computational Biology, 8*(3), e1002405. https://doi.org/10.1371/journal.pcbi.1002405

7. It is not clear to me why it should be the case, mathematically, that the first SPC always captures the broadband activity and the second SPC always captures the narrow band gamma activity. I could believe this could be the case in practice, but not mathematically guaranteed. What's to prevent the NBG from being captured by the third SPC instead in some cases? It strikes me that rather than simply taking the components with the top two eigenvalues, it would make more sense to scrutinize the properties of the components to see which, if any, have the expected properties of broadband or NBG activity.

Response: We agree with the reviewer that the first and second components do not mathematically guarantee a separation between the BB and NBG components. Hence, our approach was a data-driven one in which we indeed scrutinized the properties of the components for their selection based on the method described below, which has now been explicitly defined in the Methods section:

Line 793-816: "*We identified NBG components using a run-length threshold applied independently to each spectral principal component (SPC) and electrode. For each SPC, we evaluated the frequency range 30–80 Hz (51 samples). An NBG component in a given electrode was marked present if this window contained a consecutive run spanning at least 75% of the window with magnitudes strictly greater than 0 (i.e., ≥ 39 consecutive samples within 30–80 Hz). Applying this criterion independently yielded a single NBG component in 4/7 electrodes in S1, 5/6 electrodes in S2, 63/63 electrodes in M1 (V1), and 0/63 electrodes in M1 (V4). Across S1, S2, and M1, the NBG component corresponded to SPC 2 in all cases. Similarly, the BB components were selected with the same procedure over 1–200 Hz (200 samples). A BB component in a given electrode was marked present if this window contained a consecutive run spanning at least 75% of the window with magnitudes strictly greater than 0 (i.e., ≥150 consecutive samples within 1–200 Hz). Using this criterion, we found a single BB component in 7/7 electrodes in S1, 6/6 electrodes in S2, and 63/63 electrodes in M1 in both V1 and V4. The BB component corresponded to SPC 1 across S1, S2, and M1. The average across channels for NBG and BB components following this procedure is depicted in* ***Figure S1****.*"

We have also included in **Figure S1** the variance explained by the BB and NBG components selected using the method described above, along with a visualization of the SPC3 and SPC4 components.

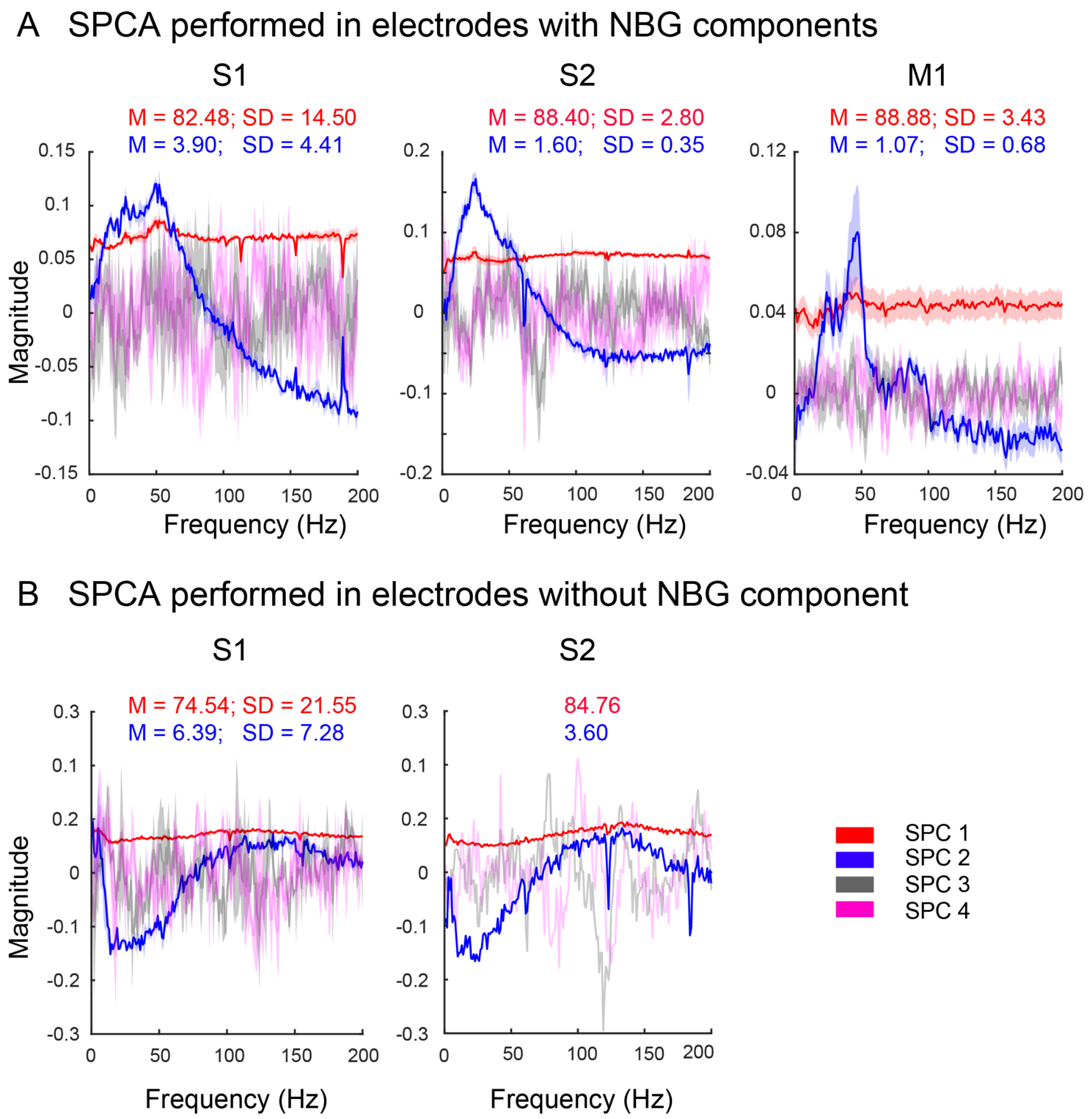

Figure S1. *SPCA component for S1, S2, and M1.* ***(A)*** *Averaged traces showing a characteristic NBG component for S1 (4 out of 7 electrodes), S2 (5 out of 6 electrodes), and M1 (63 out of 63 electrodes). While PC1 (red) does not show a characteristic peak across frequencies (BB component), PCA 2 (blue) shows increased magnitude spanning the gamma band (NBG). The mean and standard deviation of the variance explained by the PC1 and PC2 components across channels are depicted in red and blue, respectively. Unlike PC1 and PC2, PC3 (gray) and PC4 (magenta) exhibit high channel variability without a characteristic peak.* ***(B)*** *Averaged traces for electrodes without a characteristic NBG component for S1 (2 out of 7 electrodes), S2 (1 out of 6 electrodes). The mean and standard deviation of the variance explained by the PC1 and PC2 components across channels are depicted in red and blue, respectively. In contrast, PC1 shows a broadband component, and PC2 mainly peaks in the lower-frequency range (<10 Hz).*

8. On that note, since the objective is to separate components that are assumed to be superimposed, would not independent components analysis (or factor analysis), rather than principal components analysis, be the more appropriate method for the stated objectives? Principal component analysis does not include source separation as an explicit objective (i.e., mixtures of sources can be captured in a single PC if the covariance is large enough), whereas ICA or FA do so.

Response: We thank the reviewer for this relevant comment, as it is vital to consider the suitability of the analytical technique to the research questions and possible alternatives. Our choice of spectral PCA (SPCA) to separate broadband and rhythmic components follows extensive prior work showing that SPCA isolates a broadband component that robustly tracks local cortical activation and task engagement across tasks and areas (e.g., Miller et al., 2009; 2014; 2019; Sabra et al., 2020; Canales-Johnson et al., 2021; Gelens et al., 2024; Jensen et al., 2024). Critically, SPCA-based separation has been successfully applied to intracranial recordings, including human ECoG recordings in motor (individual finger movements) and language (verb generation) tasks (Miller et al., 2009; Miller et al., 2014). It has also been extended to non-human primate recordings in the auditory cortex (Canales-Johnson et al., 2021; Gelens et al., 2024). In these SPCA decompositions, the leading component (PSC1) typically captures the broadband shift, while PSC2 typically captures a narrow-band rhythmic pea. Thus, we chose SPCA due to its strong theoretical and empirical backing.

Noteworthily, we are not claiming strict neural source separation; instead, we view broadband and oscillatory components as distinct stages of sensory inference, with broadband supporting rapid sensory inference and oscillations maintaining information in local circuits, consistent with recent theoretical accounts that delineate transient (aperiodic) versus oscillatory dynamics (Vinck et al., 2023; Dowdall, Schneider & Vinck, 2023; Vinck et al., 2025). Accordingly, we do not believe that the lack of strict source separation with PCA renders our claims invalid.

References:

- Canales-Johnson, A., Teixeira Borges, A. F., Komatsu, M., Fujii, N., Fahrenfort, J. J., Miller, K. J., & Noreika, V. (2021). Broadband dynamics rather than frequency-specific rhythms underlie prediction error in the primate auditory cortex. Journal of Neuroscience, 41(45), 9374–9391. https://doi.org/10.1523/JNEUROSCI.0367-21.2021
- Dowdall, J. R., Schneider, M., & Vinck, M. (2023). Attentional modulation of inter-areal coherence explained by frequency shifts. NeuroImage, 277, 120256. https://doi.org/10.1016/j.neuroimage.2023.120256
- Gelens, F., Äijälä, J., Roberts, L., Komatsu, M., Uran, C., Jensen, M. A., Miller, K. J., Ince, R. A. A., Garagnani, M., Vinck, M., & Canales-Johnson, A. (2024). Distributed representations of prediction error signals across the cortical hierarchy are synergistic. Nature Communications, 15, 3941. https://doi.org/10.1038/s41467-024-48329-7
- Miller, K. J., Zanos, S., Fetz, E. E., den Nijs, M., & Ojemann, J. G. (2009). Decoupling the cortical power spectrum reveals real-time representation of individual finger movements in humans. Journal of Neuroscience, 29(10), 3132–3137. https://doi.org/10.1523/JNEUROSCI.5506-08.2009
- Miller, K. J., Honey, C. J., Hermes, D., Rao, R. P. N., den Nijs, M., & Ojemann, J. G. (2014). Broadband changes in the cortical surface potential track activation of functionally diverse neuronal populations. NeuroImage, 85(Part 2), 711–720. https://doi.org/10.1016/j.neuroimage.2013.08.070
- Sabra, Z., Bonilha, L., & Naselaris, T. (2020). Spectral encoding of seen and attended object categories in the human brain. Journal of Neuroscience, 40(2), 327–342. https://doi.org/10.1523/JNEUROSCI.0900-19.2019
- Vinck, M., Uran, C., Spyropoulos, G., Onorato, I., Broggini, A. C., Schneider, M., & Canales-Johnson, A. (2023). Principles of large-scale neural interactions. Neuron, 111(7), 987–1002. https://doi.org/10.1016/j.neuron.2023.03.015
- Vinck, M., Uran, C., Dowdall, J. R., Rummell, B., & Canales-Johnson, A. (2025). Large-scale interactions in predictive processing: Oscillatory versus transient dynamics. Trends in Cognitive Sciences, 29(2), 133–148. https://doi.org/10.1016/j.tics.2024.09.013

9. How did the authors determine whether NBG was “observed” in a component? Was this done just by visual inspection, or was some objective, quantitative testing done? Ideally this should be objective. What was the authors’ criterion for considering a gamma band to be “narrow”? For example, in figure 2a the bandwidth of the NBG curves is, in the opinion of this reviewer, quite wide (especially for the human subjects: for S2 the magnitude of the signal is roughly the same from 12–60 Hz (please add finer graduations to the abscissas on these plots, which would greatly ease our ability to interpret these).

Response: Thanks for the opportunity to clarify the criteria for selecting NBG components in the Methods section, including a new Supplementary **Figure S1** that provides detailed analyses. See our response to **point 7**.

Similarly, we have now added finer graduations to the abcissas in **Figure 2** and in **Supplementary Figures S3 and S4,** which relate to this and other points (see below).

10. It is not clear if the authors calculate MI at the single-channel level, or if this is multivariate.

Response: We thank the reviewer for pointing this out. We have edited the following paragraph to add more detail about the MI analysis in the Methods section:

LINES 816-830: *"In the present study, S represents the experimental contrast between grating and noise (or natural images) stimuli, and R corresponds to either the broadband (BB) or narrowband gamma (NBG) univariate response (i.e., single-electrode)"*

11. On p.3 when the authors describe "[quantifying] the difference between image categories", it is not clear if they mean e.g. gratings vs. noise, or the various categories of natural images, or high or low homogeneity, or anything else. It should be clearer what are the categories being compared.

Response: We thank the reviewer for pointing out this ambiguity. The "image categories" referred to here are gratings and noise for humans and gratings and natural images for the macaque: the analytical contrast was always the highest homogeneity (i.e., gratings) against the lowest homogeneity (i.e., noise or natural images). We have rephrased the sentence as follows:

Lines 161-171: *"Specifically, we computed MI between the neural response R (BB or NBG) and the stimulus category S (gratings vs noise in humans; grating vs natural image in macaque). MI quantifies, in bits, the reduction in uncertainty about S given R and was estimated with the Gaussian Copula MI framework (see Methods). This provides a continuous measure of category discriminability and is evaluated at each time point per electrode with FWER-controlled permutation testing. Thus, we used MI separately for the BB and NBG responses to quantify differences between image categories on a standardized effect-size scale."*

12. In the results (p. 3) when the authors report "We observed significant information encoded in…" not enough information is given about the nature of the statistical test here, in the figure legend, or in the methods. The results text doesn't even state what the type of test was (permutation test we eventually learn), but we should also know which variables were shuffled and along which dimensions, the number of iterations that were performed, etc.

Response: We thank the reviewer for this comment and acknowledge how this might make interpreting the findings difficult. As per journal convention, we reported the methods only in the methods section. However, to add clarity, we have now inserted a paragraph describing the methods (both information-theoretical analysis and statistics) in more detail into the results section as well:

Line 818-848: *"Within the framework of information theory, Mutual Information (MI) between the neural response (R) and the stimulus category (S) quantifies the reduction in uncertainty about one variable given knowledge of the other. Formally, MI can be written in several equivalent forms:* $I(R;S) = H(S) - H(S|R) = H(R) - H(R|S) = H(S) + H(R) - H(R,S)$*, where* $H(X)$ *denotes the marginal entropy of variable X,* $H(X|Y)$ *the conditional entropy, and* $H(X,Y)$ *the joint entropy. Intuitively,* $I(R;S)$ *measures how much information (in bits) the neural response conveys about the stimulus category. In the present study, S represents the experimental contrast between grating and noise (or natural images) stimuli, and R corresponds to either the broadband (BB) or narrowband gamma (NBG) univariate (i.e., single-electrode) neural response. Thus, MI provides a measure of effect size that*

quantifies how strongly neural activity discriminates between stimulus classes on a common information-theoretic scale. A value of 1 bit indicates that the response completely resolves the uncertainty between two equally probable stimulus categories, whereas smaller values indicate partial reductions in uncertainty. This formulation enables a direct comparison of BB and NBG responses in terms of their relative encoding strength. MI was estimated using the Gaussian Copula Mutual Information (GCMI) framework (Ince et al., 2017), which provides bias-corrected, rank-based estimates suitable for neural time-series data. Statistical significance across time points was assessed via 1000 permutations (shuffling stimulus label) and a maximum-statistics correction controlling the family-wise error rate at α = 0.05."

13. There seems to be a bit of inconsistency between the result description and the figure captions for their MI data (figure 2). In page 3: "We observed significant information encoded in the BB (dark green) and NBG (light green) ..."; but then in the figure caption (fig. 2c): "between gratings (dark green) and noise images (light green)". The legend inset in the plots also says dark green = BB (not dark green = gratings, as implied in the figure legend). Is the figure legend incorrect? It's hard to understand how MI would be computed *within* a stimulus category, since I think the MI is about image category itself.

Response: We sincerely thank the reviewer for noting the issue with Figure 2. This allowed us to notice that, beyond what the reviewers mention, we plotted an older version of the figure. In the current **Figure 2**, the columns of SPCA, BB, NBG, and MI are flipped between Subject 1 and 2. Furthermore, the labeling for gratings and noise trials was also flipped for S1. We have now corrected both issues in the **new Figure 2**, which resolves some of the variability described by the reviewer. We have also corrected the figure legend accordingly. The new Figure 2 and legend are plotted below:

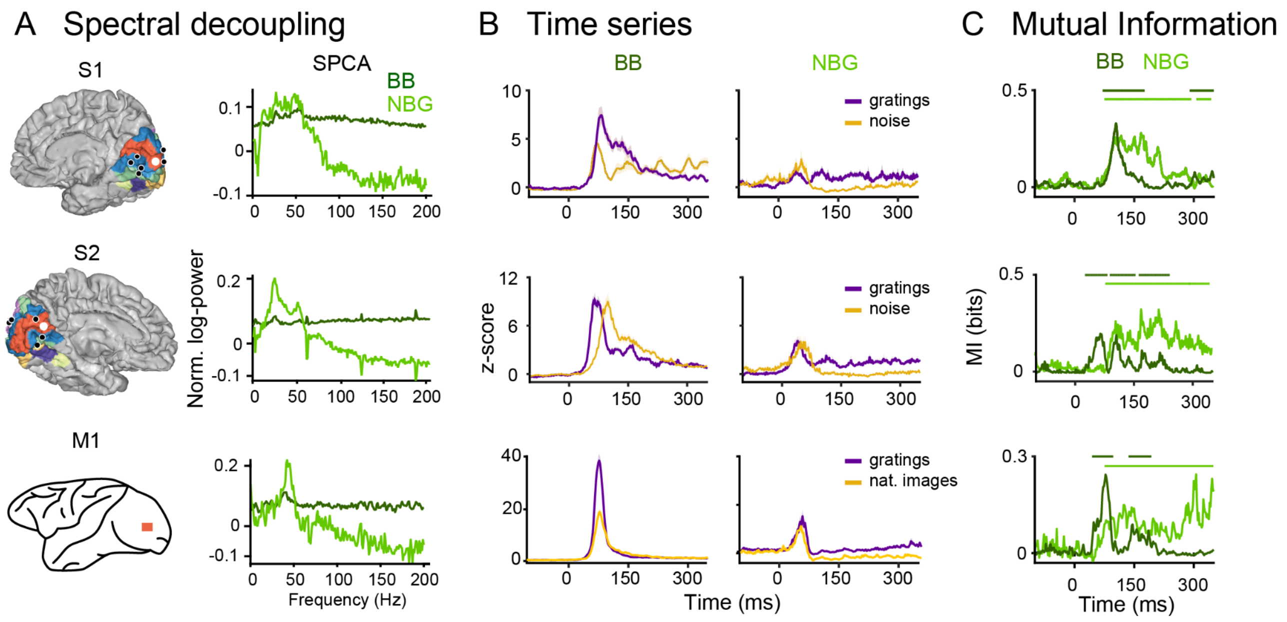

***Figure 2.** Spectral decoupling and mutual information analyses. **(A)** Spectral decoupling from a V1 electrode in Subject 1 (S1) and Subject 2 (S2) (in white), and from a V1 electrode in Macaque 1 (M1) (in orange). The magnitude (i.e., the normalized log-power) of the first (dark green) and second (light green) spectral principal component (SPCA) is shown between 1 and 200 Hz. The first SPCA is the non-oscillatory, broadband (BB) component, and the second SPCA is the oscillatory, narrow-band gamma (NBG) component. **(B)** Time-series average across gratings (purple), and noise (yellow) trials, reconstructed with the BB (left panel) and NBG components (right panel) for S1, S2, and M1. **(C)** Mutual information analyses between gratings (dark green) and noise images (light green) for S1 and S2, and between gratings and natural images for M1.*

Mutual information (in bits) quantifies the effect size of the difference between the corresponding image categories, representing the amount of information encoded in the signals about the stimulus difference. The significant time points after a permutation test are shown as bars over the MI plots.

14. Throughout the manuscript, the authors report approximate values where it seems they should have made precise measurements under quantifiable uncertainty. For example, "This was observed in S1 (BB: ~40-150 ms)..." (p. 3). It would be more professional to report the actual measures of central tendency and the dispersion, such as "...S1 (BB: 39.6(0.2) ms [mean(SD)] to 150.2(0.6) ms…"

Response: Thanks, we have now reported the exact onset of the significant MI effects, and a new supplementary Figure (Figure S3), showing the full results across electrodes and subjects.

Line 171-186: *"We observed significant information encoded in the BB (dark green) and NBG (light green) signals after contrasting gratings versus noise in S1 and S2, and gratings versus natural images in M1 (**Figure 2C**, and **Figure S3B**). Although both BB and NBG responses encoded significant information about the spatial homogeneity of images, we did observe some differences in latencies between the two types of dynamics (**Figure 2C**, and **Figure S3B**). Across electrodes and subjects (**Figure S3B**), the earliest significant MI effects appeared in the BB signals, while the MI effects in the NBG signals emerged later. Specifically, in S1, MI effects in the BB signals emerged as early as 28 ms after stimulus onset compared to the 60 ms onset of the NBG signals (**Figure S3B;** upper panel). Similarly, in S2, BB effects began at 48 ms while NBG effects began at 80 ms (**Figure S3B;** middle panel). In M1, BB effects began at 50 ms and NBG effects at 66 ms (**Figure S3B;** lower panel)."*

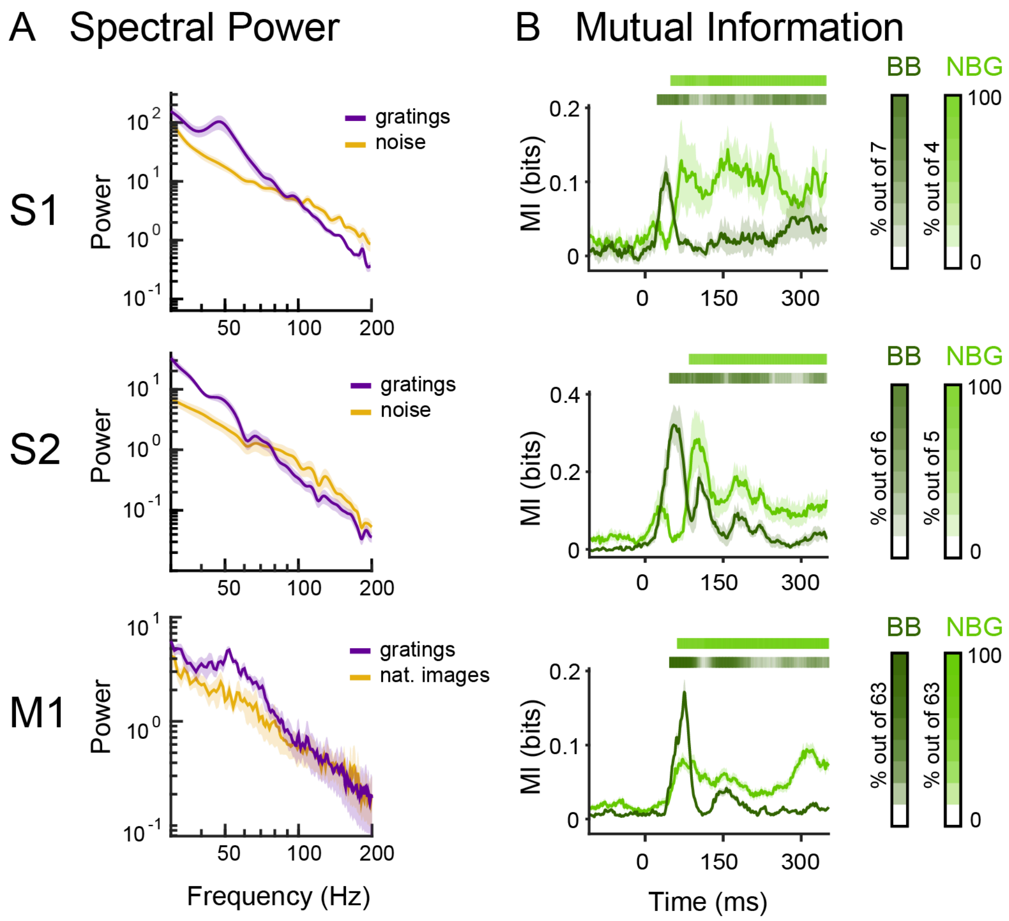

Figure S3. *Spectral power and mutual information for S1, S2 and M1.* ***(A)*** *Power spectral density for gratings (purple) and noise (yellow) stimuli for S1 and S2, and for gratings and natural images (yellow) for M1. Error bars represents the standard error of the mean (S.E.M) across visual areas (S1: V1,V2; S2: V1, V2, V3, and M1: V1).* ***(B)*** *Mutual information analyses between gratings and noise images for the BB component for S1 and S2 (dark green), and between gratings and natural images for M1 (dark green). Mutual information between gratings and noise images for the NBG component for S1 and S2 (light green), and between gratings and natural images for M1 (light green). Error bars represent S.E.M across electrodes. The shaded horizontal bars represent significant MI values at the single-electrode level, with colors indicating the percentage of electrodes showing a significant effect at each time point for BB (dark green) and NBG (light green).*

15. P.4: “Redundancy quantifies the degree to which two time points carry the same trial-by-trial information about the stimulus contrast.” What do the authors mean by “contrast” here? Luminance contrast? Or a specific type of comparison between conditions maybe? Perhaps the authors intended “homogeneity” rather than “contrast”?

Response: Here, “stimulus contrast” refers to the analytical contrast between the two classes of images. In other words, redundancy quantifies the degree to which two time points carry the same trial-by-trial information about whether the stimulus belonged to class 1 or class 2. We appreciate how, in the context of a visual paper, the word “contrast” can be misleading and thank the reviewer for pointing this out. The sentence has been reformulated:

LINES 205-208: *“Redundancy quantifies the degree to which two time points carry the same trial-by-trial information about the stimulus category (i.e., high vs. low homogeneity).*

16. How do the authors determine whether a pattern of redundancy/synergy is “diagonal” or “blocky”? Is this by visual inspection? Or is there an objective, quantitative test? For this claim, the test should be objective.

Response: We understand the concern about describing qualitative patterns. However, in this manuscript, we follow the established practice introduced in the temporal-generalisation framework (King & Dehaene, 2014): “diagonal” versus “block” patterns are descriptive, qualitative labels applied to the full time x time matrices. King & Dehaene (2014), and various other researchers in the field (Ranieri et al., 2022; Hebart et al., 2018; Mohsenzadeh, 2018; Spaak et al., 2017; Stokes, 2015) likewise interpret these shapes visually and do not define a separate numeric “diagonality/blocky” test/quantitative index. Our usage mirrors this convention: we present the complete matrices and use the same terminology solely as standard descriptors of their organisation. As our co-information analysis is conceptually similar to the temporal-generalisation framework (but captures additional synergistic information beyond temporal generalisation, which captures redundant representations), we believe this is the correct language to use to relate the findings to the existing literature. We have now explicitly stated these are qualitative terms:

Line 212-218: *“In qualitative terms, a diagonal is a pattern described by King and Dehaene (2014) as a chain, where a sequence of discrete generators represent the stimulus over time. In contrast, a square pattern is a qualitative term that indicates a sustained response, which is more likely to result from a single process maintained over time.”*

References:

- King, J. R., & Dehaene, S. (2014). Characterizing the dynamics of mental representations: the temporal generalization method. *Trends in cognitive sciences*, *18*(4), 203-210.

- Hebart, M. N., Bankson, B. B., Harel, A., Baker, C. I., & Cichy, R. M. (2018). The representational dynamics of task and object processing in humans. eLife, 7, e32816. https://doi.org/10.7554/eLife.32816
- Mohsenzadeh, Y., Qin, S., Cichy, R. M., & Pantazis, D. (2018). Ultra-rapid serial visual presentation reveals dynamics of feedforward and feedback processes in the ventral visual pathway. eLife, 7, e36329. https://doi.org/10.7554/eLife.36329
- Ranieri, G., Benedetto, A., Ho, H. T., Burr, D. C., & Morrone, M. C. (2022). Evidence of serial dependence from decoding of visual evoked potentials. Journal of Neuroscience, 42(47), 8817–8825. https://doi.org/10.1523/JNEUROSCI.1879-21.2022
- Spaak, E., Watanabe, K., Funahashi, S., & Stokes, M. G. (2017). Stable and dynamic coding for working memory in primate prefrontal cortex. Journal of Neuroscience, 37(27), 6503–6516. https://doi.org/10.1523/JNEUROSCI.3364-16.2017
- Stokes, M. G. (2015). 'Activity-silent' working memory in prefrontal cortex: A dynamic coding framework. Trends in Cognitive Sciences, 19(7), 394–405. https://doi.org/10.1016/j.tics.2015.05.004

17. P.4: "We observed more temporal redundancy in NBG…" this phrasing is ambiguous as to whether "more redundancy" means greater intensity, longer duration, some combination thereof, or something else. If I'm piecing this together correctly, what the authors mean here is that they found a greater cardinality of significant time points.

Response: We thank the reviewer for pointing out this ambiguity. They were indeed right. We have edited the paragraph to state:

LINES 240-241: *"We observed more temporal redundancy, as measured by the number of significant time points, in NBG…"*

18. In figure 2, what is "Magnitude"? Given the methods –which describes computing the PSD and then performing PCA –I was expecting something in volts (or perhaps volts squared, or volts^2/Hz). There is no method described for normalizing or scaling the SPCs into something called "magnitude".

Response: We thank the reviewer for pointing this out. The y-axis in Figure 2 ("Magnitude") shows the normalized log-power over frequencies. To clarify this, we have changed the y-axis to "norm. log-power" and added the following sentence to the figure caption: *"Y-axis shows unitless SPC normalized log-power; positive values indicate frequencies that increase when the component's activation is positive."*

19. When computing the PSDs for the SPCA procedure, isn't the phase information lost? I don't understand how an inverse transformation into the time domain could be done properly if the phase information is discarded (should result in complex values in the time domain). I would appreciate more clarity on this method.

Response: We thank the reviewer for the comment and for pointing out the ambiguity in the method. It is worth noting that our analysis differs from those that reconstruct the time-frequency-domain waveform. In our study, SPCA is applied to normalized power spectra. We never reconstruct the time-frequency-domain waveform; instead, we take the principal component and, for each time point, estimate how much of it is present in the log-power spectra. This yields a single activation number for each time point, which can be used to reconstruct the power spectrum. Notably, we never invert to the time domain; thus, no phase is needed, and no complex values appear. More information about the SPCA method has been added to the methods section:

Line 759-785: ***"****To separate broadband (BB) from narrowband gamma (NBG) activity, we applied spectral principal component analysis (SPCA) to the trial-wise power spectral densities (PSDs) obtained from each electrode. For each subject, we first computed the PSD of the ECoG signal for every trial and concatenated these spectra into a two-dimensional matrix (frequency × trials). Each column represents the spectral power profile of a single trial, and each row represents the distribution of power across trials at a given frequency. We then normalized the PSD at each*

*frequency by dividing by the mean power across trials and log-transformed the resulting values to stabilize variance. From this normalized log-power matrix, we computed the covariance between all pairs of frequencies. Thus, each element of the covariance matrix reflects how fluctuations in power at one frequency covary with those at another frequency across trials. This frequency-by-frequency covariance matrix was then decomposed into eigenvectors, yielding orthogonal spectral principal components (SPCs) ordered by the variance they explain. The first SPC typically captured broadband (aperiodic) modulations spanning 1–200 Hz, while the second SPC isolated narrowband gamma activity (30–80 Hz) with a characteristic spectral peak (**Figure S1**). For each component, we reconstructed the corresponding time-domain signal by weighting and summing the original ECoG time series according to the component's loading vector, thereby obtaining separate BB and NBG time courses for subsequent analyses."*

20. The data are initially and exclusively presented at a high level of processed abstraction. Inclusion of less processed results (e.g., spectrograms of example channels) would improve the report and make readers better able to interpret the quality of the data.

Response: We thank the reviewer for this comment and agree that examples of more "raw" data would make the results and data quality more interpretable. We have added the raw spectra and MI differences for all electrodes and subjects, and summarized them in the new **Figures S3, S4,** and **S5:**

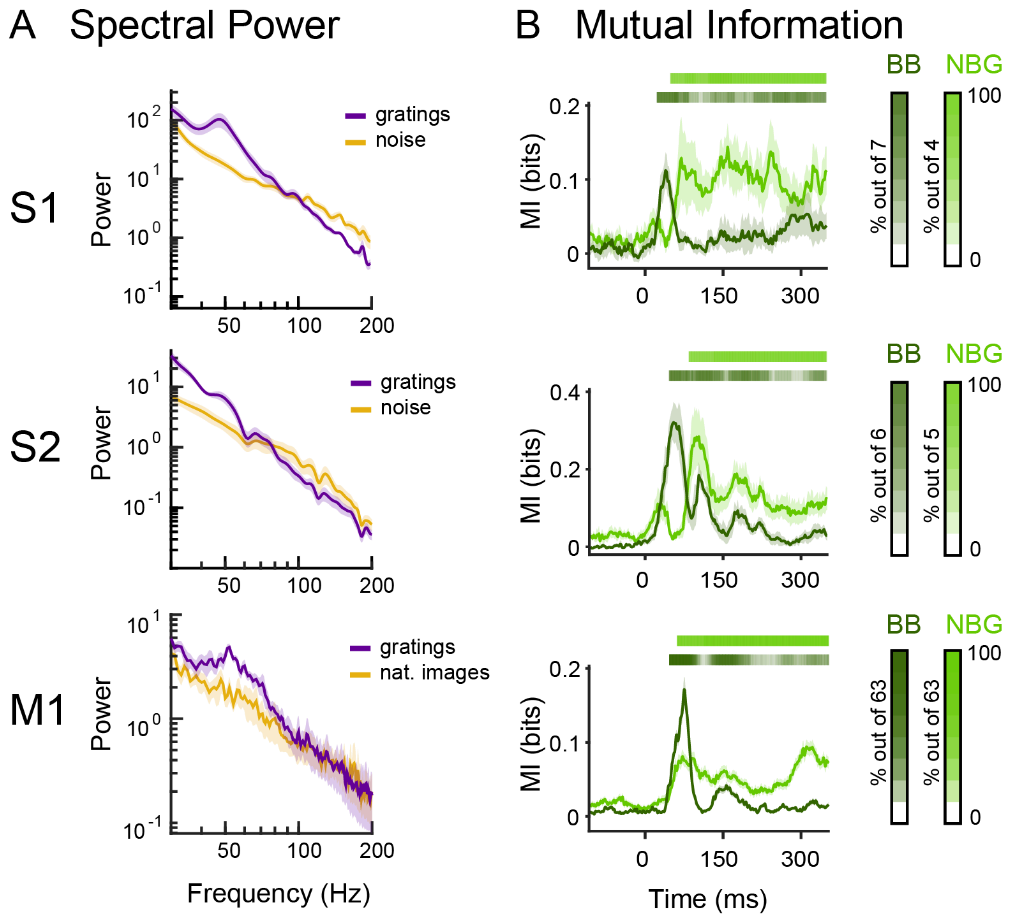

***Figure S3.** Spectral power and mutual information for S1, S2 and M1. **(A)** Power spectral density for gratings (purple) and noise (yellow) stimuli for S1 and S2, and for gratings and natural images (yellow) for M1. Error bars represents the standard error of the mean (S.E.M) across visual areas (S1: V1,V2; S2: V1, V2, V3, and M1: V1). **(B)** Mutual information analyses between gratings and noise images for the BB component for S1 and S2 (dark green), and between gratings and natural images for M1 (dark green). Mutual information between gratings and noise images for the NBG*

component for S1 and S2 (light green), and between gratings and natural images for M1 (light green). Error bars represent S.E.M across electrodes. The shaded horizontal bars represent significant MI values at the single-electrode level, with colors indicating the percentage of electrodes showing a significant effect at each time point for BB (dark green) and NBG (light green).

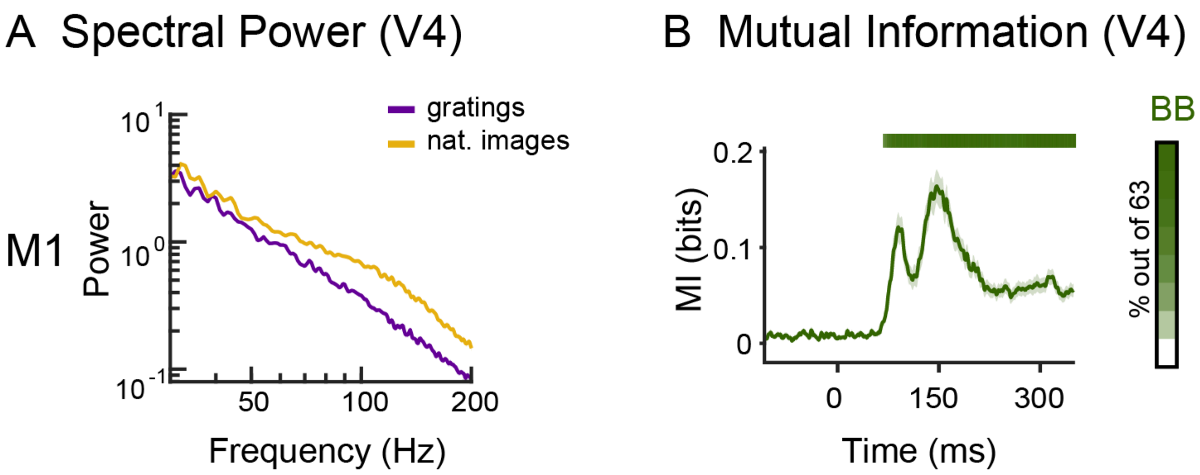

Figure S4. *Spectral power and mutual information for M1 in area V4.* ***(A)*** *Power spectral density for gratings (purple) and noise and natural images (yellow). Error bars represent the standard error of the mean (S.E.M) across electrodes in V4.* ***(B)*** *Mutual information analyses between gratings and natural images for M1 (dark green). Error bars represent S.E.M across electrodes. The shaded horizontal bars represent significant MI values at the single-electrode level, with the colors representing the percentage of electrodes showing a significant effect per time point for BB (dark green).*

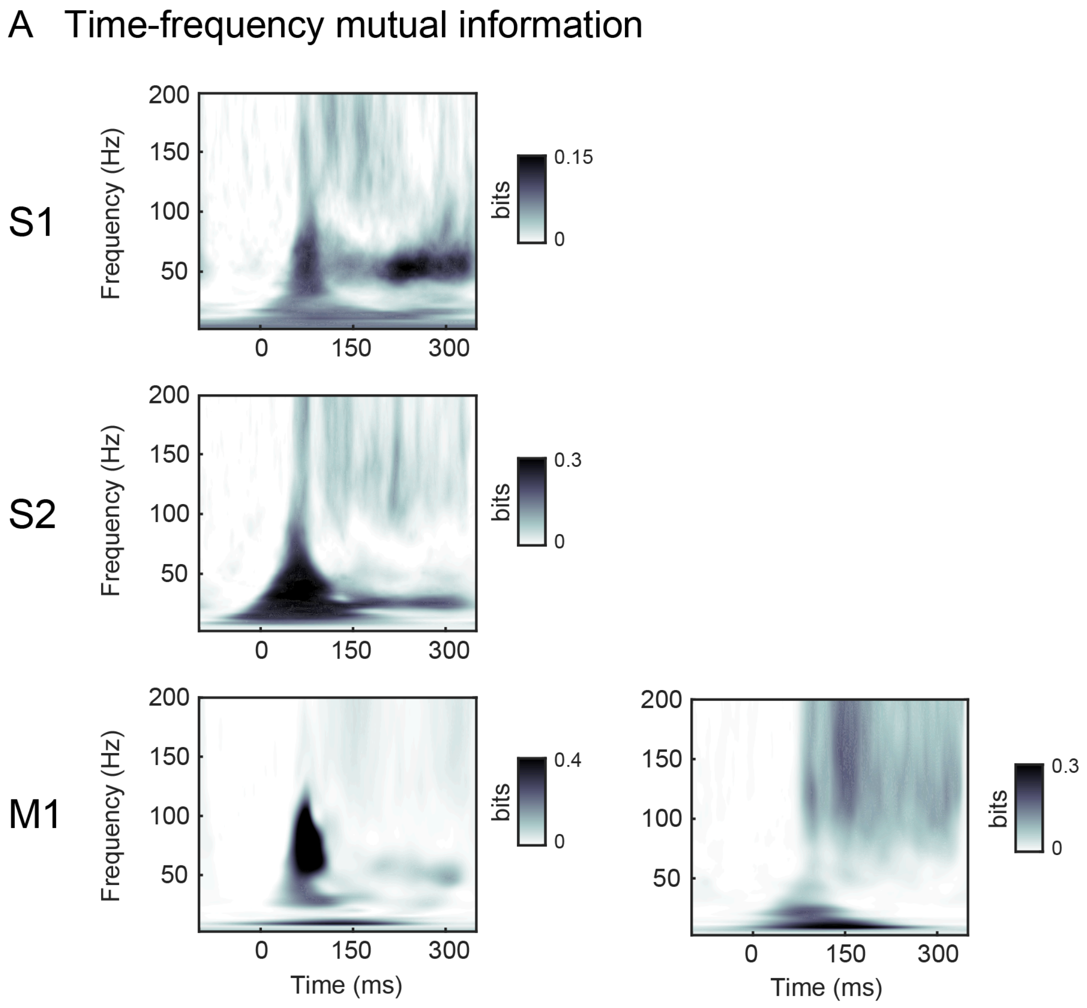

Figure S5. Time-frequency mutual information for S1, S2 and M1. (A) Mutual information (MI) between gratings and noise for S1 and S2, and between gratings and natural images for M1. MI was computed from power values across trials for each time-frequency bin from 1 to 200 Hz and for the -100 to 350 ms window around stimulus onset. MI represents the effect size (in bits) of the difference in spectral power for each corresponding stimulus comparison. Plots represent the average MI across electrodes (S1: 7 electrodes [V1 and V2 area]; S2: 6 electrodes [V1, V2 and V3 area], M1: 63 electrodes in V1 (left panel), and 63 electrodes in V4, right panel).

21. Can the authors rule out the influence of microsaccades? One could imagine that microsaccades when viewing a spatially heterogeneous image would lead to a greater change in the retinal input than microsaccade when viewing a spatially homogenous image. Here I mean homogeneity in my intuitive understanding in that a white noise pattern has a more homogenous texture than a grating. The predicted pattern of results under such an interpretation would look exactly like the NBG plots in figure 2B.

Response: The effect of microsaccades on these neural markers is in itself an interesting methodological question, but for the current experiment, we believe it to be irrelevant for the following reasons: Firstly, microsaccade-related artifacts affect both gamma-band and broadband dynamics both for EEG/MEG (Schwartzman et al., 2011; Kupers et al., 2018) and intracranial recordings (Lower et al., 2016), making a selective influence on only one of the components improbable. Secondly, a single ocular artifact provides no precise mechanism for producing opposing information-theoretic signatures; redundancy in gamma and synergy in broadband. Because our question centers on the information content of these signals, and the observed dissociation is not plausibly explained by microsaccades, any residual contribution (if present) is

inconsequential for our conclusions. We further note that, firing rates are generally low for grating stimuli (e.g. see Zhou, 2008; Gieselmann &Thiele 2008), which runs counter to the explanation of the reviewer. We also emphasize that the "noise" stimuli here are not white noise, but are band-pass filtered for S1 and S2, and for M1, are natural images.

References:

- Gieselmann, M. A., & Thiele, A. (2008). Comparison of spatial integration and surround suppression characteristics in spiking activity and the local field potential in macaque V1. *European Journal of Neuroscience*, *28*(3), 447-459.
- Kupers, E. R., Wang, H. X., Amano, K., Kay, K. N., Heeger, D. J., & Winawer, J. (2018). A non-invasive, quantitative study of broadband spectral responses in human visual cortex. *PLoS One*, *13*(3), e0193107.
- Lowet, E., Roberts, M. J., Bosman, C. A., Fries, P., & De Weerd, P. (2016). Areas V1 and V2 show microsaccade-related 3–4-Hz covariation in gamma power and frequency. *European Journal of Neuroscience*, *43*(10), 1286-1296.
- Schwartzman, D. J., & Kranczioch, C. (2011). In the blink of an eye: the contribution of microsaccadic activity to the induced gamma band response. *International Journal of psychophysiology*, *79*(1), 73-82.
- Zhou, Z., Bernard, M. R., & Bonds, A. B. (2008). Deconstruction of spatial integrity in visual stimulus detected by modulation of synchronized activity in cat visual cortex. *Journal of Neuroscience*, *28*(14), 3759-3768.

22. The vertical axis in figure 3c is labeled as "%", but the maximum value on that axis is less than 0.5, i.e. less than half of one percent, which would be quite a small value for this. Do the authors perhaps mean to label this rather as a *proportion* of significant time points, rather than percent? This comment applies to like figures.

Response: We thank the reviewer for pointing out this mistake. We did indeed mean "proportion", and this has been fixed in the figures.

23. P.5: "This off-diagonal synergy has a characteristic pattern…" How did the authors determine that this pattern is characteristic of temporal synergy within BB signals. Is there an objective, quantitative test for this, or is this a qualitative opinion of the authors? Later in the same sentence: "...aligned with each temporal axis…" Again, is this handwaving, or is there some quantitative testing behind this claim? For this claim its probably acceptable to use qualitative interpretation by the authors, but it should be clear what the basis for the claim is.

Response: We thank the reviewer for this helpful suggestion and agree that clearer wording would be beneficial. We use the phrase "characteristic off-diagonal synergy" as a qualitative descriptor within the temporal-generalization framework (King & Dehaene, 2014), in which off-diagonal structure indicates cross-temporal interactions, whereas diagonal structure reflects strictly time-locked codes (Please also see our response to **point 16**) . Notably, a similar off-diagonal synergy motif has been reported in an ECoG dataset from marmosets performing auditory oddball tasks analyzed with a comparable information-theoretic pipeline (Gelens et al., 2024), suggesting that this pattern generalizes across species and recording modalities. We have revised the manuscript accordingly.

24. Figure 2 (and others): it is not reported which measure of dispersion is represented by the shading.

Response: We thank the reviewer for spotting this mistake; it has now been clarified in all figures that the shading represents the standard error of the mean (S.E.M) and the corresponding relevant dimension of the mean.

25. P.7: "Similarly to the results observed within areas, interareal interactions…" What evidence do the authors have for interaction between the brain areas here? If I'm following correctly, this same analysis could be applied, e.g., to two populations recorded in different monkeys viewing the same

stimulus set on different days in different laboratories. The analysis tests whether the information in two populations is redundant or synergistic, but does not depend on interaction between the populations, as far as I can tell.

Response: We thank the reviewer for pointing out this ambiguous phrasing. We would like to emphasize that here we use the term "interaction" to refer to a statistical interaction between cortical sites. We would also like to point out that, in our analysis, we do not simply relate neural activity across different nodes as many classical network analyses do. Instead, we compute the MI/Co-i between neural activity and stimulus identity. In other words, we show what kind of information different neural signals/regions **carry about the stimulus**. As a result, any synergistic information between regions necessarily reflects a superadditive increase in information **about the stimulus** when both signals are considered together.

This experimental control puts us in the regime of "randomised control trial", the gold standard of experimental design, instead of the "correlational study", which would reflect more classical network analyses. However, as the reviewer rightfully points out, no mechanistic accounts of brain function can be drawn from synergy itself, as any number of mechanisms could give rise to it. Yet, in support of our interpretation, a possible candidate mechanism for synergy has been identified in a study that employed an identical information theoretic analytical pipeline: Gelens et al. (2024) show that in a brain-constrained neurocomputational model, synergistic information between areas only emerges when there are are long-range recurrent anatomical connections between different levels of the model.

To emphasise both of these points, we have added the following paragraph in the discussion:

LINES 420-438: *"We would like to emphasize that synergy in our study is a statistical descriptor of joint neural interactions, not a mechanistic model of the underlying neurophysiology. Mechanistic inferences, therefore, require converging evidence from modelling and/or targeted causal perturbations. In support of our interpretation of synergy as recurrent processing between brain areas, Gelens et al. (2024) showed that in a brain-constrained neurocomputational model matched to marmoset ECoG, temporofrontal synergy emerged only when strong long-range recurrent (feedback and feedforward) "jumping" connections were included; purely feedforward or locally recurrent variants did not produce synergy. Thus, this observed inter-areal synergy is consistent with an interpretation of recurrent processing across cortical levels. We note, however, that synergy can also arise from global state fluctuations (e.g., attention/arousal) or other non-stimulus-specific factors, so we present this as a candidate mechanism to be tested with targeted perturbations and further modelling."*

References:

- Gelens, F., Äijälä, J., Roberts, L., Komatsu, M., Uran, C., Jensen, M. A., ... & Canales-Johnson, A. (2024). Distributed representations of prediction error signals across the cortical hierarchy are synergistic. *Nature Communications*, *15*(1), 3941.

26. Figure 6, the ordinate tick marks are unlabeled.

Response: Thanks. This has now been fixed.

27. First sentence of Methods says the recordings in monkey were ECoG, whereas the Results says the recordings in monkey were LFP. Please clarify. Note also that Hermes et al., 2019 does not seem to report monkey (Methods says "data acquisition details are reported in full in [that paper]").

Response: We thank the reviewer for pointing out this mistake. The monkey recordings were LFP, and this has now been fixed.

28. Hermes et al., 2019 –to which we are directed to learn the full methodological details –describes three human subjects, not two. Was one subject excluded from this, and why?

Response: This project aimed to quantify co-I ***both*** within and between electrodes. Hence, our criteria for selecting subjects were that each subject must have at least one electrode in two *distinct* visual areas. The subject the reviewer is referring to did not match this criterion (i.e., it has been implanted with two electrodes in V1). Hence, we did not include this patient.

29. The Methods reports the monkey subject as "Monkey H" whereas the rest of the report uses a different identifier ("M1"). However, in order to understand the methods, which are very brief in this report, the authors point to Peter et al., 2019, which refers to one subject as "Monkey H", which I'm assuming is this same monkey. So, the authors should probably use "Monkey H" throughout the report to refer to this subject.

Response: We have kept the identifier M1 throughout the manuscript to match our human labeling (i.e., S1 and S2), but explicitly clarify that M1 refers to Monkey H in Peter et al., 2019 in the Methods section.

Line 657-659: *"For further details, see (Peter et al., 2019; Uran et al., 2022). Note that M1 is referred to as Monkey H in these studies."*

30. Methods: "Trials were aborted during which the eye position deviated…" How was this determined? There is no reporting of gaze tracking methods in either this manuscript or in Hermes et al., 2019. Edit: it looks like eye tracking is reported in Peter et al., 2019. I've been really thrown off by the first sentence of the Methods that includes "monkey" and then points to Hermes et al., 2019 for "details… reported in full." Better organization and clarity would help a lot, and the authors should include enough information in this manuscript itself to understand the key details (such as that the monkey's gaze was tracked with an optical eye-tracker, that it had a Utah array implanted, etc.)

Response: Thanks. We have now made our best effort to improve readability in the revised Methods section of the manuscript.

31. Methods: "Human subjects were repeatedly presented with each type of stimulus image (15 trials per image) for a period of 500 ms…" What is the order of presentation? Are the trials randomly interlaced? Is it blocked?

Response: Trials were shown in randomised order. We have now rephrased it as:

Line 661-663: *"Human subjects were repeatedly presented with each type of stimulus image (15 trials per image) presented in a randomised order for a period of 500 ms…"*

32. "...presented… at an approximate visual angle of 20 [degrees]..." Is this an angle of eccentricity or an angle of distention? In either case, the other value should also be reported. I.e., if the stimulus is 20 degrees in distention, where in the visual field was it presented? If the stimulus was presented at 20 degrees eccentricity, then how big was it?

Response: We now report that the stimulus spanned 20 degrees of visual angle from left to right. The text now reads:

Line 663-664: *"... presented at an approximate visual angle of 20 degrees of visual angle left-to-right"*

33. What was the nature of the stimulus display (I'm guessing a computer monitor, but it isn't specified)? What was displayed between stimuli? Was there any kind of fixation point? These details are also not found in Hermes et al. 2019

Response: During the ECoG experiments, images were displayed on a computer screen placed in front of the participant. Images were presented for 500 ms (stimulus period), followed by a gray screen for 500 ms (baseline period). A small fixation dot was present at the center of the screen throughout the entire experiment. We have now added this description in the Methods:

Line XXX-YYY: *"During the ECoG experiments for S1 and S2, images were displayed on a computer screen placed in front of the participant. Images were presented for 500 ms (stimulus period), followed by a 500 ms gray screen (baseline period). A small fixation dot was present at the center of the screen throughout the entire experiment."*

34. Methods describe stimuli with curved lines, but the rest of the report only mention gratings, noise, and natural images. Please clarify.

Response: Thank you for pointing out this mistake. While the original paper (Hermes et al. 2015) also employed curved lines as stimuli, they were not analysed in this study. The reason for this is that we aimed to contrast the effects of maximally homogeneous and non-homogeneous stimuli, noise, and gratings. We have removed the section on curved lines entirely, as they are not used in this paper.

35. P.11: "...including special aperture…" I do not understand what "special" means here.

Response: Yes, we meant "spatial" aperture, thanks. Corrected in the main text now.

36. On p. 11, beginning from where it says "Unlike the human subjects, Monkey-H was presented with…" to the end of that paragraph is extremely hard to understand. The monkey only ever saw maximum of one grating in a session (possibly repeated 20 times), is that right? The whole thing about the "mode least predictable natural stimuli" and "thus selecting 80 trials" has me completely flummoxed. I cannot understand what happened here, and why.

Response: We regret the reviewer felt flummoxed by our lack of clarity. We have now clarified the entire section as follows:

Line 720-734: **"***Unlike the human participants in the original study by Uran et al. (2022), the macaque M1 was shown a set of natural images across 20 sessions. A total of 340 natural images were presented (20 trials per image). In each of the first four recording sessions, M1 was also shown a single oriented grating (20 trials per session). To identify images with the highest and lowest homogeneity in M1, we used LPIPS to determine the highest and lowest predictable natural stimuli in each session. This resulted in 80 gratings (20 images x 4 sessions = 80 images) and 80 natural-image trails (4 images with the lowest scores per session x 20 sessions = 80 images) being*

selected for further MI and co-I analyses. When comparing the predictability scores, we observed higher homogeneity for gratings (mean = 0.009; SD = 0.013) than for natural images (mean = 0.244; SD = 0.146) across images and electrodes (t(1,470) = 24.45, p < 0.001).”

37. On page 11 there’s a part that starts, “In brief, this network models the visual system as an OR-CNN,...” and then the paragraph abruptly ends mid-sentence, then there’s a one-sentence paragraph that doesn’t seem to follow, and then yet another new paragraph that also seems a bit disconnected. Something seems to be missing around here.

Response: Thanks, this phrase has been now removed.

38. Methods say “Each image was masked 26 pixels from its center in width and height”. When I first read that I was confused as to whether 26 pixels was analogous to radius or diameter. However, I become more confused when later in that paragraph a 104 x 104 pixel mask was described, which wouldn’t be consistent with 26 pixels as *either* a radius or diameter.

Response: We thank the reviewer for pointing out the need for clarification. We edited the sentence in the main text for clarification:

Lines 687-690: *“Each image was masked 26 pixels from its center in width and height (radius).”*

We corrected the part later in the paragraph:

"LPIPS predictability scores were then calculated between the model's predicted value for that central area and the true pixel values within the region for each image.”

The central area here corresponds to the 52 x 52 pixel mask, which was mistakenly typed as 104 x 104 pixel mask. We removed it altogether for clarity.

39. In the methods, when the authors write “We ranked the image categories by their predictability…” what are the categories being ranked, exactly? Just the two categories of “gratings” and “noise” (or “gratings” and “natural images”)? What is the point of this ranking step?

Response: Thanks for the comment. We have now rephrased that entire section for clarity. The new Methods section now reads like this:

Lines 690-719: *“LPIPS (Learned Perceptual Image Patch Similarity) predictability scores measure how different the predicted center of an image is from its true center, based on the surrounding context. Low scores indicate that the prediction is similar to the true center. High scores mean the prediction is dissimilar to the true center. Hence, according to our definition of image homogeneity, low scores indicate high homogeneity, while high scores indicate low homogeneity.*

Thus, to identify images with the highest and lowest homogeneity in human subjects S1 and S2, we first ranked all images by their predictability scores. Oriented grating images (80 images) showed the lowest scores (i.e., high homogeneity). Noise images (80 images) showed the highest scores (i.e., low homogeneity). We then selected these two image categories for further analysis. As a result, the MI and co-I analyses were performed on 80 gratings and 80 noise trials per subject. To confirm that the predictability scores resulted in significant differences between categories, we used a two-sided t-test to compare the scores of the pre-selected images. Gratings showed the highest image homogeneity (i.e., lowest scores) in S1 (mean = 0.171; SD = 0.081) and

S2 (mean = 0.179; SD = 0.082), and noise showed the lowest homogeneity (i.e., highest scores) in S1 (mean = 0.343; SD = 0.304) and S2 (mean = 0.307; SD = 0.274) (Figure 1B). As expected, we observed higher predictability for gratings compared to noise images across images and electrodes in S1 (t(1,110) = 4.101, p < 0.001) and in S2 (t(1,94) = 3.08, p = 0.002).

Unlike the human participants in the original study by Uran et al. (2022), the macaque M1 was shown a set of natural images across 20 sessions. A total of 340 natural images were presented (20 trials per image). In each of the first four recording sessions, M1 was also shown a single oriented grating (20 trials per session). To identify images with the highest and lowest homogeneity in M1, we used LPIPS to determine the highest and lowest predictable natural stimuli in each session. This resulted in 80 gratings (20 images x 4 sessions = 80 images) and 80 natural-image trails (4 images with the lowest scores per session x 20 sessions = 80 images) being selected for further MI and co-I analyses. When comparing the predictability scores, we observed higher homogeneity for gratings (mean = 0.009; SD = 0.013) than for natural images (mean = 0.244; SD = 0.146) across images and electrodes (t(1,470) = 24.45, p < 0.001).”

40. The claim that Spectral Principal Components “[represent] distinct neural processing components” seems to reach beyond its grasp. What is a “neural processing component”? What would make such components distinct?

Response: We thank the reviewer for pointing out this ambiguity. Here, we are interpreting the findings in the light of recent theoretical accounts (Vinck et al. 2023; Vinck et al., 2025) that propose that transient and oscillatory components of the neural signal perform distinct functions. Namely, transient activation (i.e., broadband in our study) reflects fast stimulus inference, while oscillatory dynamics reflect sustained neural states. The finding that broadband signals encode synergistic information, and (gamma) oscillations encode redundant information neatly aligns with this interpretation: stimulus inference should consist of rapid integration of complementary, stimulus-specific evidence carried by brief, asynchronous transients across sites, thereby yielding synergy, followed by the sustained maintenance and coordination of the inferred state through rhythmic synchronization that communicates information across populations. Hence, by referring to "distinct neural processing components”, we are relating our work to these recent theoretical accounts. To illustrate these points, we have added several points discussing more nuanced interpretations in therevised Discussion section.

References:

- Vinck, M., Uran, C., Dowdall, J. R., Rummell, B., & Canales-Johnson, A. (2025). Large-scale interactions in predictive processing: oscillatory versus transient dynamics. *Trends in cognitive sciences*, *29*(2), 133-148.

41. The authors perform SPCA, select components, and then perform the inverse transform back into the time domain. How is phase information used for this? Is this inverse transformation a “causal” operation, or could late time points come to influence earlier time points (acausality) through this process? More generally, to what extend does this transform/manipulate/inverse transform method have the potential to *introduce* information redundancy (or synergy) in the time domain? Have the authors tried this on simulated data reflecting the null hypothesis, or for which the ground truth of the underlying encoding mechanisms is known?

Response: We thank the reviewer for this comment, which raises legitimate concerns about the analytical method rather than the results. However, the underlying problem stems from a misunderstanding of how SPCA functions. We would like to reiterate the points made in comments 18 and 19 that no inverse transformation into the time domain is required, as SPCA operates

purely in the frequency domain. SPCA yields frequency‑domain loadings (one per component) and corresponding scores over time. The "time course" of a component at time *t* is simply its projection onto the spectral vector at *t* (i.e., a weighted sum of powers across frequency), not an inverse Fourier transform and not a reconstruction of a phase-bearing voltage signal. Phase is never estimated or used. As a result, reconstructing the component time course is simply a deterministic linear causal mapping applied pointwise in time. Thus, SPCA itself adds no acausality.

Another point of consideration is the theorem of data processing inequality, which states that post-processing cannot increase the information content of a signal (Ince et al., 2027; Nalewajski, 2011): while the content of the signal can be re-shuffled or reduced, it cannot be increased. In simple terms, the SPCA cannot introduce synergy/redundancy out of thin air. However, it is worth noting that the information can be redistributed across the components: this is indeed the rationale of the experiment, as we want to investigate whether different components SPC1 (Broadband) / SPC2 (NBG) encode different types of information (synergistic/redundant).

References:

- Ince, R. A., Giordano, B. L., Kayser, C., Rousselet, G. A., Gross, J., & Schyns, P. G. (2017). A statistical framework for neuroimaging data analysis based on mutual information estimated via a gaussian copula. *Human brain mapping*, *38*(3), 1541-1573.
- Nalewajski, R. F. (2011). Elements of information theory. In *Perspectives in Electronic Structure Theory* (pp. 371-395). Berlin, Heidelberg: Springer Berlin Heidelberg.

42. In order to compute MI, the probability of observing a particular neural datum needs to be known/estimated. Because the voltage values are continuously valued, it is highly unlikely that the exact same voltage appears more than once in the data, which prohibits estimating the probabilities from the neural data sample itself. To deal with this issue, people usually discretize their neural data observations (such as by computing a histogram and then using the number of observations in each bin to estimate probabilities). In order to interpret and replicate this analysis, we need to know how the neural data were discretized. This information is not reported.

Response: We thank the reviewer for this comment. Indeed, many methods of computing MI require binning/discretizing data. However, the lack of reporting on how the data were binned/discretized stems directly from how the Gaussian Copula Mutual Information (GCMI) method operates. A copula is a statistical structure that maps the relationship between any two marginal distributions. Any multivariate joint distribution can be expressed as a set of univariate marginal distribution functions and a copula that captures the relationship structure (Ince et al., 2017; Durante et al., 2013). As MI is a measure that depends on the relationship between two signals (distributions), it can be estimated from this relationship structure.

GCMI estimates mutual information by first converting each variable's samples to rank percentiles (empirical CDF values) and then mapping those percentiles to z-scores via the inverse standard normal CDF. This produces variables with standard-normal marginal distributions while preserving all monotonic dependence (i.e, the copula is unchanged). After this copula normalization, MI is computed from the covariance of the z-scores using the closed-form Gaussian entropy formulas (Ince et al., 2017). This yields a conservative lower bound on the true MI, with no binning required.

References:

- Durante, F., Fernandez-Sanchez, J., & Sempi, C. (2013). A topological proof of Sklar's theorem. *Applied Mathematics Letters*, *26*(9), 945-948.
- Ince, R. A., Giordano, B. L., Kayser, C., Rousselet, G. A., Gross, J., & Schyns, P. G. (2017). A statistical framework for neuroimaging data analysis based on mutual information estimated via a gaussian copula. *Human brain mapping*, *38*(3), 1541-1573.

43. Does the GCMI toolbox have any user-configurable options? If so, these should be reported.

Response: The GCMI toolbox is open-source MATLAB/Python code that is configured via function arguments (not a GUI or global settings). We used the toolbox as-is, without modifying internal code. We set only the arguments required by the public API and left the others at their defaults. Estimator choice followed the study design: because we estimate mutual information between continuous neural data and discrete stimulus classes, we used `gcmi_model_cd`. This function effectively compares a Gaussian fit (ignoring stimulus category) with a Gaussian mixture model, where each stimulus class is modelled with a separate Gaussian distribution. The analytic bias-correction option was left at its default setting (biascorrect = true). No non-default algorithmic parameters were used. Github for GCMI: https://github.com/robince/gcmi

44. Methods: "... the number of electrode pairs analyzed for the between-electrode co-I analyses were 12 for BB and 4 for NBG signal pairs for S1 (Figure 5), and 8 BB and 6 NBG signal pairs for S2 (Figure 6)." Figures 5 and 6 show monkey data; however, not human data.

Response: Thanks. This has now been fixed, and the text can be read in the reviewer's response to **point 7.**

REVIEWER COMMENTS

—---

Reviewer #1 (Remarks to the Author):

I think the authors have done a good job in responding to the reviewers' comments and suggestions. I accept the revisions in response to my own comments. Regarding those of the other reviewers I can only say they seem thorough to me, including several instances of clarifying misunderstandings, but of course I cannot speak for them. I did notice one typo in the revised ms: line 94 NGB instead of NBG - this mistake occurred in several places in the responses to reviewers so I guess it was also missed in this case in the ms.

Response: We are grateful for the reviewer's constructive criticism. We have now replaced the misspelt NGB with NBG throughout the text.

—---

Reviewer #2 (Remarks to the Author):

I appreciate the authors' effort to clarify their spectral decoupling approach for separating broadband (BB) from narrowband oscillations and more explicitly describe how narrow-band gamma oscillations (NBGs) were identified. The additional text in the results and methods sections substantially improves the readability of the analysis and strengthens the overall quality of the manuscript. The new supplementary figures are also helpful, in particular Fig. S1 for demonstrating the separation of BB and NBG in SPC1 and 2.

Overall, I think that the manuscript has much improved and it is now easier to follow the general logic and main conclusion. Below are some remaining points that I believe would further strengthen the paper:

Response: We appreciate the reviewer's constructive criticism, and we address the remaining points below.

1. When using the OR-CNN to compute homogeneity, I wonder what extent predictability depends on the training data used to construct the model. Specifically, to what degree might the model's performance reflect learned priors about natural image structure (e.g., inferring missing parts such as wheels on a car)? More generally, how sensitive is the LPIPS-based homogeneity measure to the specific training regime of the network?

Response: The OR-CNN/LPIPS pipeline is used solely to rank stimuli by predictability (LPIPS) within each dataset; no DNN features are used in neural analyses. We used the published UNet (Uran et al., 2022) with fixed weights/hyperparameters and centered inputs on each electrode's pRF. The neural MI/co-I effects reported here replicate across distinct image ensembles (band-passed gratings/noise in humans; gratings vs natural images in macaque), indicating that our conclusions do not hinge on model-specific training priors. We have clarified this point now in the Methods section:

"The OR-CNN/LPIPS pipeline was used solely to rank stimuli by predictability (LPIPS) within each dataset; no DNN features are used in neural analyses. We used the published UNet (Uran et al., 2022) with fixed weights/hyperparameters and centered inputs on each electrode's pRF. The neural MI/co-I effects reported here replicate across distinct image ensembles (band-passed

gratings/noise in humans; gratings vs natural images in macaque), indicating that our conclusions do not hinge on model-specific training priors."

2. Despite the additional descriptions, it is still unclear why the narrowband component in SPC2 is specifically referred to as a gamma oscillation. Although NBGs were identified with a threshold in the range from 30-80 Hz, the example SPCs in Figure 2 and S1 appear to have significant contributions from lower frequencies. For instance, the NBG for S2 in Fig. 2a and Fig. S1 appears to peak around ~25 Hz and falls off at higher frequencies, while the NBG for S1 rises near ~10 Hz and remains elevated only up to ~50 Hz. Specifically referring to these components as gamma oscillations therefore appears to be a potentially misleading. If the authors feel strongly that NBG is the right label to use to align with the literature I would suggest to at least change lines 128-130 which explicitly states that SPCA2 peaks in the gamma range which seems to be at odds with these results, especially the S2 spectra.

Response: We agree with the reviewer that, unlike for S1 and M1, the average SPCA2 in S2 shows in Figure S1, does not *peak* in gamma. Hence, we have rephrased lines 128-130 as follows:

"In contrast, the second spectral component exhibited a narrowband profile, spanning frequencies in the range of the oscillatory, narrowband gamma band (NBG: ~30–80 Hz)"

3. I appreciate the inclusion of the two summary figures in S3B and S4B to show that results from individual electrodes appear to be consistent across electrodes within the same object. To further improve clarity, it would be good to provide more information in the results on which statistical test was done on which data and also name the sample size. For example, for the result in lines 267-271 I assume from the text in the methods that a two-sided t-test was used to compare BB to NGB across n = 7 versus 4 across electrodes. Throughout the manuscript, it would be easier to understand if a test was done across trials, electrodes or electrode pairs by directly adding the n (e.g. n = 4 electrodes or 80 trials, 80 images etc) in the text.

Response: We now name the test, the analysis unit, and sample size inline at first mention for each result. For the specific case at lines **267–271**, we explicitly state that the comparison is a two-sided t-test on the percentage of significant time points, with the unit being electrodes (within-area) or electrode pairs (between-area), and we report n (e.g., S1: BB n = 7 electrodes vs NBG n = 4 electrodes; S2: 6 vs 5; M1/V1: 63 vs 63). We also apply the same reporting pattern to all other statistical summaries in Results and ensure that captions match Methods:

"*To directly compare potential differences in how BB and NBG signals encode redundant representations about image predictability, we quantified and statistically contrasted the temporal extension of redundancy between BB and NBG by a two-sided t-test on the percentage of significant time points per electrode (see Methods). We observed more temporal redundancy, as measured by the number of significant time points, in NBG compared to BB signals in S1 ($t(1,9) = 2.83$; $p = 0.019$; n = 4 vs. n = 7; Figure 3C); and S2 ($t(1,12) = 3.90$; $p = 0.003$; n = 5 vs. n = 6; Figure 3F); and M1: ($t(1,62) = 8.30$; $p < 0.001$; n = 63 vs. n = 63; Figure 5C), and also more generally in co-I (S1: $t(1,9) = 2.30$; $p = 0.046$; S2: $t(1,12) = 3.28$; $p = 0.009$; M1: $t(1,62) = 7.70$ $p < 0.001$)."*

"To compare these synergistic patterns to the redundant ones, we quantified and statistically contrasted the temporal extension of synergy between BB and NBG signals. Again, we compared BB and NBG by a two-sided t-test on the percentage of significant time points per electrode (see Methods). Interestingly, and in contrast to temporal redundancy, we observed more temporal

synergy in BB compared to NBG signals in S1 ($t(1,9) = 3.06$, $p272 = 0.017$; $n = 7$ vs. $n = 4$; Figure 3C); and S2 ($t(1,12) = 3.27$, $p = 0.012$; $n = 6$ vs. $n = 5$; Figure 3F), and M1 ($t(1,62) = 2.42$, $p = 0.016$; $n = 63$ vs. $n = 63$; Figure 5C)."

"We quantified and statistically contrasted differences between BB and NBG signals as described above, this time between the corresponding visual areas. We compared BB and NBG by a two-sided t-test on the percentage of significant time points per electrode pair (see Methods). Again, we observed higher temporal redundancy in NBG compared to BB signals in S1 ($t(1,9) = 3.90$; $n = 6$ pairs vs. $n = 21$ pairs; $p = 0.003$; Figure 4C); and S2 ($t(1,12) = 7.39$; $p < 0.001$; $n = 10$ pairs vs. $n = 15$ pairs; Figure 4F), and also in co-I (S1: $t(1,9) = 6.46$; $p < 0.001$; S2: $t(1,12) = 6.10$; $p < 0.001$)."

Reviewer #3 (Remarks to the Author):

The authors have addressed in a satisfactory manner all of my concerns and suggestions.

Response: We are grateful for the reviewer's constructive criticism.

Reviewer #4 and #5 (Remarks to the Author):

Note to the editor: Please note we have numbered the reviewer's comments.

I have a mixed reaction to this revision. Some of the more straightforward remedies have been tackled, but the more fundamental issues have not been satisfyingly addressed. The methods reporting is greatly improved. However, the greater clarity has exacerbated, rather than assuaged, my concerns about how the authors are constructing isolated-component time series in their analysis. I appreciate the inclusion of some less-processed, descriptive analyses of the data. I continue to disagree with the use of "homogeneity" here, and find that that terminology confuses, rather than clarifies. I continue to think that the use of homogeneity as an independent variable has not been adequately motivated. Some of the responses from the authors do not seem to engage with the concerns substantively (e.g., on the training history of the DNN and the potential impacts, or on the impacts of microsaccades). The response about microsaccades was especially disappointing, considering that the authors reported being in possession of eyetracking data that could speak to this rigorously.

1. I'm still not on board with the authors' use of the term "homogeneity", which simply seems incorrect. They have not persuaded me otherwise with the revision. The methods refers to a "predictability score"... why not just use this, more intuitive label for the metric of interest? It seems unnecessarily confusing to re-label this measure as something unintuitive.

The authors seem to have made no attempt to address my question about the behavioral relevance / ethological validity of their "homogeneity" measure. Why is this interesting/important? Why "homogeneity" as opposed to any other stimulus property? The authors' guiding thesis seems to be that BB is more internal/inferential sort of processing whereas NBG is more external/stimulus processing, so shouldn't the same analysis work for practically any stimulus feature you might want to try this with? Why or why not? The authors' motivation for "homogeneity"

seems to boil down to (paraphrasing): “we looked at this because we thought it would be correlated with at least one of our dependent measures given the literature”, which is not satisfying. They also say that they’re not interested in homogeneity itself, but are instead interested in NBG and BB activity. However, why should the readers be interested in those phenomena either? If the only motivation is to link them to another phenomenon that we also haven’t been given any reason to care about, then that is not effective.

Response:

1. In our paper, “homogeneity” was *operationally defined* as center predictability from the surround measured with LPIPS on the OR-CNN outputs (i.e., low LPIPS implies high predictability, hence, high “homogeneity”) and is used strictly to rank stimuli; the neural inferences (MI/co-I) are computed on ECoG/LFP only. This operationalization and its implementation details were explicitly described in Methods: Image homogeneity analysis, and the Results describe the category checks in both humans and macaques. This makes the construct transparent and separable from the neural effects. However, following the reviewer's advice, we have changed to “structural predictability”. This is similar to the terminology we have used in other papers (Vinck & Bosman, 2016; Peter et al., 2019; Uran et al., 2022).

2. Regarding the relevance of the structural predictability variable, it is well established that structural predictability is a strongly validated driver of narrow-band gamma in visual cortex across species and paradigms (e.g., Hermes et al., Uran et al., Shirhatti et al.; cited in our *Introduction/Results*). We explicitly leverage this fact to place BB vs NBG on a shared, information-bearing axis—so that their redundancy/synergy profiles can be meaningfully compared. Concretely, we confirm that structural predictability ranks stimuli as expected (gratings with high predictability vs. noise/natural stimuli with low predictability) and then show that *both* BB and NBG carry MI about that contrast before decomposing co-information. This is the minimum principled setup for testing the computational roles (redundant vs. synergistic) of BB and NBG. The argument and statistics are already present in the text.

3. Regarding the question “could any feature have worked?” Not necessarily. The literature shows that NBG is selectively modulated by specific *structural* image statistics (including homogeneity/predictability), whereas BB responds broadly to visual input. Our data reproduce this pattern: MI is present in both signals, yet co-I reveals qualitatively different dynamics (i.e., sustained redundancy for NBG; off-diagonal/transient synergy for BB). The point of our design is precisely to contrast how each signal family represents a *shared*, behaviorally relevant dimension, rather than to scan features arbitrarily.

2. I am not at all moved by the authors’ response about accounting for the training history of the model (i.e., that the model was trained on natural images but not the other stimulus classes, and how might this impact things). They seem to have made no real attempt to address this. Another reviewer (at least one) also raised this issue.

Response: The OR-CNN/LPIPS pipeline is used solely to rank stimuli by predictability (LPIPS) within each dataset; no DNN features are used in neural analyses. We used the published UNet (Uran et al., 2022) with fixed weights/hyperparameters and centered inputs on each electrode’s pRF. The neural MI/co-I effects reported here replicate across distinct image ensembles (band-passed gratings/noise in humans; gratings vs natural images in macaque), indicating that our conclusions do not hinge on model-specific training priors. We have clarified this point now in the Methods section:

“The OR-CNN/LPIPS pipeline was used solely to rank stimuli by predictability (LPIPS) within each dataset; no DNN features are used in neural analyses. We used the published UNet (Uran et al., 2022) with fixed weights/hyperparameters and centered inputs on each electrode’s pRF. The neural MI/co-I effects reported here replicate across distinct image ensembles (band-passed gratings/noise in humans; gratings vs natural images in macaque), indicating that our conclusions do not hinge on model-specific training priors.”

3. In their rebuttal the authors state that they do not inverse transform back to the time domain after their SPCA procedure. However, the legend to Figure 1 clearly states: “BB signals are computed by reconstructing the time series of each stimulus category trial with the first spectral principal component (SPCA) of the ECoG/LFP signal”, which implies that the time-domain signal of the BB component is reconstructed. If this is not, in fact, what was done, the authors should revise this (and similar) statement(s) to not be so misleading. Actually, now that I’ve had another pass of the revision after originally writing this point, I see that the authors took their SPC loadings and multiplied those by the original time-domain data to reconstruct BB time series alone (for example). This raises a number of new issues that I deal with separately below.

On the point of time-domain signal reconstruction, the authors have revised to write: “For each component, we reconstructed the corresponding time-domain signal by weighting and summing the original ECoG time series according to the component’s loading vector, thereby obtaining separate BB and NBG time courses for subsequent analyses.” This seems improper, as described. After the PSD step, polarity information at the electrode would be lost if the authors are just taking the magnitudes (unless the “loadings” used by the authors are complex-valued, which they wouldn’t seem to be –this still has not been cleared up in the revision). I don’t understand how this could work. The math is well-formed, but the result would in no way reflect a reconstruction of the broad-band activity alone. Can the authors show their analysis on some realistic simulated data where the ground truth is known and demonstrate that this does, in fact, reconstruct the correct broadband time series? Also, the fact that I still can’t tell whether the PSD is complex-valued in this case or not is another concern. I would have a lot easier time of this if the authors simply reported the sequence of equations that they used, and explained what each variable was, its dimensionality and units etc. E.g., “ ‘A’ is a nTrials x nFrequencies matrix of complex-valued Fourier coefficients in microvolts….” Finally, tying into my point from the previous round of reviews about how ICA would be a more appropriate approach for this study (which the authors dismissed in their rebuttal), because the spirit of the analysis as described by the authors assumes independence that PCA does not guarantee.

In one of their rebuttals the authors write “...as SPCA operates purely in the frequency domain…” This conflicts with the description for reconstructing the time-domain signal of the broadband activity using the SPCA loadings. Granted, the SPCA results are just frequency-domain, but then the authors did this time-domain reconstruction. I understand that constructing the time series of the component scores doesn’t require phase information, but I’m not concerned about the time series of component scores. Throughout these concerns about time-domain reconstruction I have been specifically concerned with the step now described on line 781 of the revised manuscript. That step does not seem proper as described (albeit mathematically well-formed), and the rebuttal does not address this. Also, in reading line 117 (“The time series for each trial was then reconstructed by using the the weights from the first and second SPC, separately…”), the typical way to “reconstruct” a time series from some PC result is to take some set of component scores and multiply those times the loadings to get back into the original domain. In the absence of more clarification, this would be the consensus assumption as to what was done. However, the authors

are not doing that. Rather, they are multiplying the SPC loadings by the original time-series data, which, even if it wasn't highly dubious, is at least highly atypical, and thus behooves a bit more detail as to precisely what was done.

Response: The reviewer's critique rests on a misinterpretation we can eliminate by clarifying the exact sequence of operations, which confirms that our approach is proper:

1. For each electrode and trial, we compute complex Morlet wavelet transforms, square magnitudes to obtain band-power over time, discard edges, normalize each band's power to unit mean, and take the log (a normalization step).

2. We form a log-power time-by-frequency matrix and then project it onto a spectral principal component (SPC), i.e., we take the dot product of each time slice with the SPC frequency-loading vector, yielding a single-component time course (a time series of ***scores*** in log-power units).

This further clarifies that we do not reconstruct raw voltage; rather, we reconstruct component scores in log-power space. This is a power-domain method; phase/polarity are irrelevant to that quantity and are correctly omitted. The full description, including the dimensionalities already laid out in Figure S2, will be reproduced verbatim in Methods as equations to avoid ambiguity. This addresses the reviewer's specific concern that "polarity information… would be lost," as we are not reconstructing polarity-bearing voltages. We will also replace any residual phrasing that could be read as "multiplying SPC loadings by the raw time-domain voltage" with the precise description above. Where the main text currently says "reconstructed using the weights of the first/second SPC" (e.g., in Fig. 1C legend and §Results opening), we will change it to "*projected log-power onto the SPC frequency loading to obtain component time courses.*" The specific changes in the Methods section can be found below:

Spectral PCA on trial-wise power spectra. For each electrode, we computed a power spectral density (PSD) for every trial and stacked these spectra into a frequency-by-trials matrix $P(f, q)$, where f indexes frequency bins (retaining positive frequencies up to 200 Hz) and q indexes trials ($q = 1, \ldots, N_q$). We normalized each frequency bin by its mean power across trials and applied a natural-log transform:

$$\hat{P}(f, q) = \ln P(f, q) - \ln\left(\frac{1}{N_q}\sum_q P(f, q)\right). \tag{1}$$

We then formed a frequency–frequency covariance matrix across trials,

$$C(f, f') = \sum_q \hat{P}(f, q)\,\hat{P}(f', q), \tag{2}$$

and performed eigendecomposition,

$$C\,\mathbf{e}_j = \lambda_j\,\mathbf{e}_j, \qquad \lambda_1 \geq \lambda_2 \geq \ldots, \tag{3}$$

yielding orthogonal spectral principal components (SPCs) $\mathbf{e}_j(f)$ ordered by explained variance. In our datasets, the first component (SPC1) captured broadband (aperiodic) modulations spanning 1–200 Hz, while the second component (SPC2) isolated a gamma-band spectral motif concentrated in the 30–80 Hz range.

Component-specific time courses (projection in the time–frequency domain). To obtain a component-specific univariate time series per trial, we computed a time–frequency representation for each trial using a bank of complex Morlet wavelets (5 cycles per frequency). Let $V(f, t, q)$ denote the complex wavelet coefficient at frequency f, time t, and trial q. We computed band-power over time, discarded convolution edge samples, normalized each band to unit mean over time, and applied a natural-log transform:

$$\hat{P}(f, t, q) = \ln\left(\frac{|V(f, t, q)|^2}{\frac{1}{N_t}\sum_t |V(f, t, q)|^2}\right), \tag{4}$$

where N_t is the number of time samples in the analyzed window. We then projected the log-power matrix onto each SPC vector to yield a single component time course:

$$a_j(t, q) = \sum_f e_j(f)\,\hat{P}(f, t, q). \tag{5}$$

We treated $a_1(t, q)$ as the broadband (BB) component time course and $a_2(t, q)$ as the narrowband gamma (NBG) component time course. These component time series were epoched from -100 ms to 350 ms around stimulus onset and baseline-corrected by subtracting the mean of the pre-stimulus interval (-100 to 0 ms). For macaque data, reconstructed time courses were downsampled to 500 Hz to match the human sampling rate used in downstream analyses.

Second, regarding the use of SPCA rather than ICA, our goal is not to assume statistical independence among generators but to obtain orthogonal spectral motifs that capture the covariance structure of band-power with trial-level temporal precision. **SPCA does exactly that and is the field-standard for dissociating aperiodic BB and narrow-band oscillations in ECoG/LFP** (Miller 2009; Miller 2019; Canales-Johnson et al., 2021; Gelens et al., 2024; Sabra et al., 2020; Jensen et al., 2025, etc, all cited in Methods and reiterated below). ICA on band-power time-frequency data is not guaranteed to yield physiologically interpretable *spectral* motifs and imposes non-Gaussian/independence assumptions that are unnecessary for our objective.

References:

- Canales-Johnson, A., Teixeira Borges, A. F., Komatsu, M., Fuji, N., Fahrenfort, J. J., Miller, K. J., & Noreika, V. (2021). Broadband dynamics rather than frequency-specific rhythms underlie prediction error in the primate auditory cortex. **Journal of Neuroscience**, 41(45), 9374–9391. https://doi.org/10.1523/JNEUROSCI.0367-21.2021
- Gelens, F., Äijälä, J., Roberts, L., Komatsu, M., Uran, C., Jensen, M. A., Miller, K. J., Ince, R. A. A., Garagnani, M., Vinck, M., & Canales-Johnson, A. (2024). Distributed representations of prediction error signals across the cortical hierarchy are synergistic. **Nature Communications**, 15, 3941. https://doi.org/10.1038/s41467-024-48329-7
- Jensen, M. A., Fine, A., Kerezoudis, P., Wong-Kisiel, L., Alden, E. C., Hermes, D., & Miller, K. J. (2025). Functional mapping of movement and speech using task-based electrophysiological changes in stereoelectroencephalography. **Journal of Neurosurgery**, 142(2), 311–323. https://doi.org/10.3171/2024.5.JNS24434.
- Miller, K. J., Zanos, S., Fetz, E. E., den Nijs, M., & Ojemann, J. G. (2009). Decoupling the cortical power spectrum reveals real-time representation of individual finger movements in humans. **Journal of Neuroscience**, 29(10), 3132–3137. https://doi.org/10.1523/JNEUROSCI.5506-08.2009
- Miller, K. J. (2019). A library of human electrocorticographic data and analyses. **Nature Human Behaviour**, 3(11), 1225–1235. https://doi.org/10.1038/s41562-019-0678-3
- Miller, K. J., Hermes, D., Pestilli, F., Wig, G. S., & Ojemann, J. G. (2017). Face percept formation in human ventral temporal cortex. **Journal of Neurophysiology**, 118(5), 2614–2627. https://doi.org/10.1152/jn.00113.2017.
- Sabra, Z., Bonilha, L., & Naselaris, T. (2020). Spectral encoding of seen and attended object categories in the human brain. **Journal of Neuroscience**, 40(2), 327–342. https://doi.org/10.1523/JNEUROSCI.0900-19.2019

4. The authors rebuttal about microsaccades is not persuasive. They write:
"Firstly, microsaccade-related artifacts affect both gamma-band and broadband dynamics both for EEG/MEG (Schwartzman et al., 2011; Kupers et al., 2018) and intracranial recordings (Lower [sic] et al., 2016), making a selective influence on only one of the components improbable. …"
First, how improbable is "improbable"? This comment is not rigorous. Also, are both NBG and BB affected in precisely the same way? All we need for a confound is a differential effect. Also, did those previous studies cited by the authors use the same/similar stimuli as the current report? Because the interaction with stimulus processing is a central component of the study, and so comparison to those precedents needs to account for the stimulus differences. The authors rebuttal continues:
"....Secondly, a single ocular artifact provides no precise mechanism for producing opposing information-theoretic signatures; redundancy in gamma and synergy in broadband."
Again, we don't need microsaccades to simultaneously have opposing effects on two phenomena, they only need to associate with one of the phenomena to produce a confound. Moreover, this claim that a singular artifact cannot provide opposing effects on the two spectral components is given simply as conjecture without support, and so is not persuasive.
".... We further note that, firing rates are generally low for grating stimuli (e.g. see Zhou, 2008; Gieselmann &Thiele 2008), which runs counter to the explanation of the reviewer. We also emphasize that the "noise" stimuli here are not white noise, but are band-pass filtered for S1 and S2, and for M1, are natural images." My comment made no mention of firing rate, and firing rate and precise stimulus class are not substantive dimensions of the concern. If there is any spatial variation in stimulus properties across the retina, and anything about microsaccades differs between stimulus conditions (frequency, amplitude, direction, velocity, etc) then this is a potential confound, simply put. Any persuasive response to the microsaccade question MUST include some analysis of eye movement data from the experiment. The authors reported they possess those data. I would struggle to accept a version of this manuscript without such an analysis. Even then, it doesn't guarantee that the microsaccades aren't an issue, but it's an essential starting point.

Response: We added the following text to the discussion:

*"It is possible that differences in neural activity between stimulus categories may relate to microsaccadic activity. We analyzed microsaccades in the available eye-tracking dataset (macaque M1) across different image categories. These results do not reveal an association with microsaccades (**Figure S7**). Regardless, in this study, we do not make a claim regarding the neural mechanisms mediating the effect of structural predictability on neural activity. To our knowledge, the mechanisms underlying such contextual modulation are not fully established. For*

example, there are several biophysical models (Jadi & Sejnowski, 2014; Tahvili & Vinck & DiVolo, 2025), and indications that SOM neurons are more active for unpredicted stimuli (Keller et al., 2020), which in turn promote gamma oscillations. However, direct evidence for these mechanisms in primates and humans is lacking, and differences between rodents and primates warrant consideration (Onorato et al., 2020). Microsaccades are an unlikely mechanism for the relationship between structural predictability and NBG, for various reasons: Classic findings on gamma oscillations with size tuning have been made under anesthesia in monkeys (e.g., Jia, Tanabe & Kohn, 2013; Jia, Xing & Kohn, 2013), where one can rule out eye-movement-related changes in activity, and the difference in gamma between noisy and grating images is also found in anesthetized, paralysed cats (Zhou and Bonds, 2008). In Figure 1 and Figure Supplement 3 of Peter et al. (2019), we have shown that V1 gamma is not affected by restricting the analyses to periods outside microsaccades. Hence, the literature suggests that differences in NBG between structurally predicted and unpredicted stimuli are not driven by microsaccades. Likewise, gamma responses in monkey visual cortex are transiently disrupted by saccades during free viewing but then emerge image-specifically after each saccade (Brunet et al., 2014).”

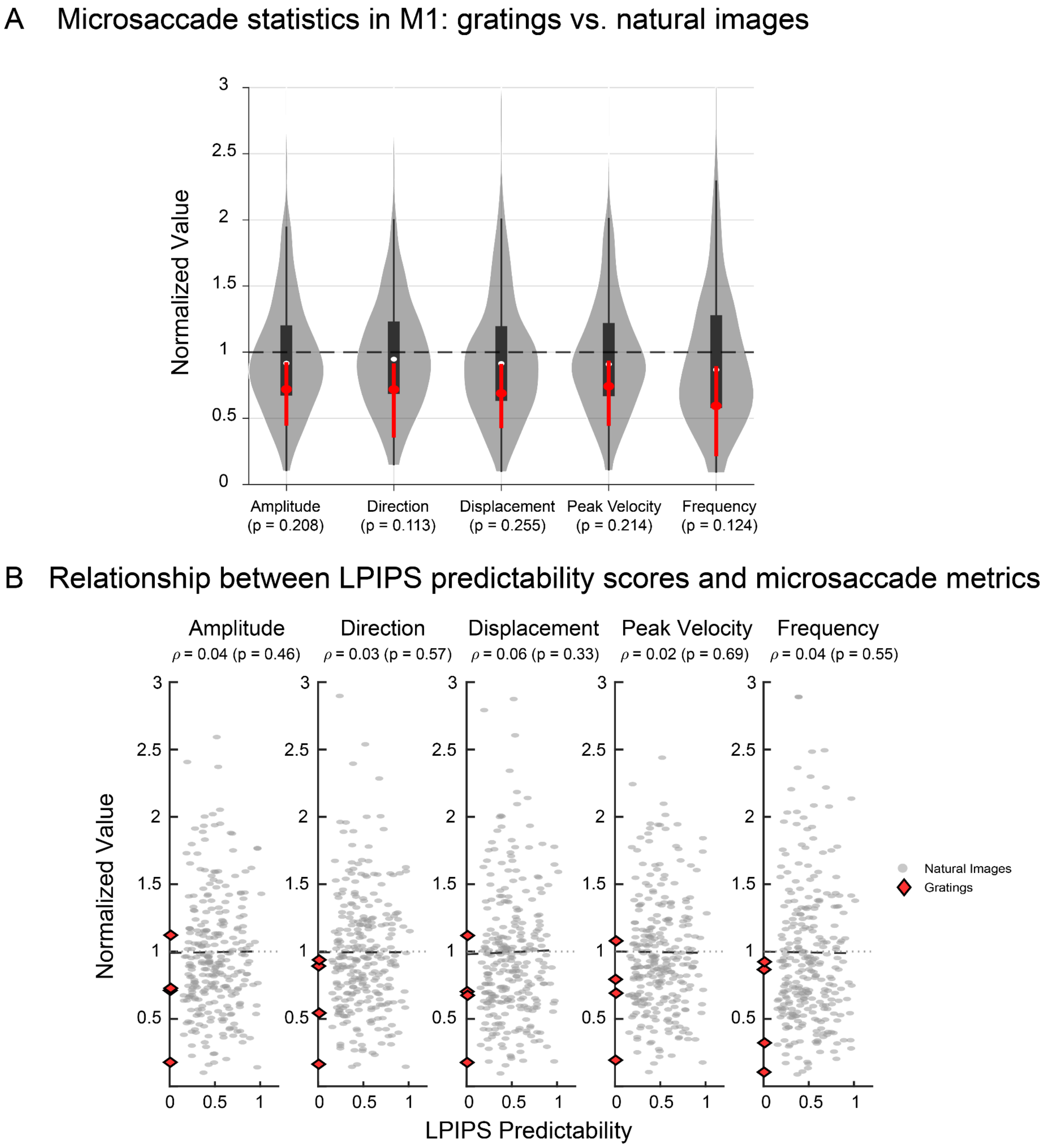

***Figure S7**. Eye Movement Analysis. To address potential oculomotor confounds, we analyzed eye-tracking data collected during the presentation of both natural images and gratings, which were used in all other analyses. **(A)** Microsaccades were detected using the algorithm as described in Otero et al. (2014). We extracted five kinematic metrics for comparison: amplitude, direction, displacement, peak velocity, and frequency. For each metric, we compared the distributions of the natural images and gratings. For visualization, all metric values were normalized to the mean of the Natural Image condition (Natural images Mean = 1). We assessed statistical significance using the nonparametric Wilcoxon rank-sum test. In the resulting plots, the natural image distribution is shown as a violin plot (gray), while the gratings are summarized by their median (red circle) and interquartile range (red vertical line). **(B)** The relationship between LPIPS predictability scores and microsaccade metrics. Grating stimuli are shown in red, and natural images are shown in gray. Grating stimuli follow the same distribution as natural images; they do not form an outlier cluster that drives a spurious trend. Across the pooled dataset, we found no significant correlation between image statistics and microsaccade dynamics (Spearman correlation, $p > 0.05$ for all metrics).*

We have added in the Methods section:

***"Eye movement recordings and microsaccade detection.** Eye movements and pupil size were recorded binocularly at 1000 Hz using an Eyelink 1000 system (SR Research) with infrared illumination. Eye signals were calibrated before each recording session using a standardized fixation task. Microsaccades were detected using an unsupervised clustering method described by Otero et al. (2014). Candidate microsaccades were identified as periods during which eye velocity exceeded 5 degrees/sec. Saccade onset and offset were defined as the first and last points, respectively, where the velocity or acceleration exceeded the detection thresholds surrounding the peak velocity. We excluded microsaccades occurring during blinks or inter-trial intervals, based on eyelink pupil data and blink events. Overlapping saccade candidates were merged, and a minimum inter-saccadic interval of 30 ms was enforced. For each detected microsaccade, we calculated metrics including amplitude (Euclidean distance between the start and end positions), direction (angle of the amplitude vector), peak velocity, and microsaccade duration. Microsaccade frequency (Hz) was calculated as event count per trial duration."*

References

- Otero-Millan, J., Castro, J.L.A., Macknik, S.L., Martinez-Conde, S. (2014). Unsupervised clustering method to detect microsaccades. **Journal of Vision** 14.
- Jadi, M.P., Sejnowski, T.J., 2014. Cortical oscillations arise from contextual interactions that regulate sparse coding. **Proceedings of the National Academy of Sciences** 111, 6780–6785.
- Jia, X., Tanabe, S., Kohn, A., 2013a. Gamma and the coordination of spiking activity in early visual cortex. **Neuron** 77, 762–774.
- Jia, X., Xing, D., Kohn, A., 2013b. No consistent relationship between gamma power and peak frequency in macaque primary visual cortex. **The Journal of Neuroscience** 33, 17–25.
- Peter, A., Uran, C., Klon-Lipok, J., Roese, R., Van Stijn, S., Barnes, W., Dowdall, J.R., Singer, W., Fries, P., Vinck, M., 2019. Surface color and predictability determine contextual modulation of V1 firing and gamma oscillations. **eLife** 8, e42101.
- Zhou, Z., Bernard, M.R., Bonds, A.B., 2008. Deconstruction of Spatial Integrity in Visual Stimulus Detected by Modulation of Synchronized Activity in Cat Visual Cortex. **The Journal of Neuroscience** 28, 3759–3768.
- Brunet, N.M., Bosman, C.A., Vinck, M., Roberts, M., Oostenveld, R., Desimone, R., De Weerd, P., Fries, P., 2014. Stimulus repetition modulates gamma-band synchronization in primate visual cortex. **Proceedings of the National Academy of Sciences** 111, 3626–3631.
- Onorato, I., Neuenschwander, S., Hoy, J., Lima, B., Rocha, K.S., Broggini, A.C., Uran, C., Spyropoulos, G., Klon-Lipok, J., Womelsdorf, T., Fries, P.,Niell, C., Singer, W., Vinck, M., 2020. A distinct class of bursting neurons with strong gamma synchronization and stimulus selectivity in monkey V1. **Neuron** 105, 180–197.
- Tahvili, F., Vinck, M., di Volo, M., 2025. Pv and som cells play distinct causal roles in controlling network oscillations and stability. **Cell Reports** 44, 116131.

5. The legend for Figure 2 still doesn't match what is shown. The legend states "...first (dark green) and second (light green) spectral principal component (SPCA) is shown between 1 and 200 Hz." But the inset says "BB" and "NBG" not "first" and "second". Arbitrarily associating the ordinality of

the component with assumed spectral content was a main concern shared among the reviewers. The authors changed from using ordinality to some operational definition, so the legend should reflect that change.

Response: The association is not arbitrary as we demonstrated that, in our data, our first component always corresponded with BB, and our second component always corresponded with NBG (see response to **point 6**).

6. The revised results section continues to talk about the “first” and “second” SP components as if those ordinal labels were meaningful, but the authors stated that in the revision they switch to a functional method for identifying BB vs. NBG components, so this way of presenting the results no longer makes sense.

Response: The nomenclature SPC1 and SPC2 makes sense even after our run-length functional analysis, so we kept it. As we clearly stated in our previous response, *across S1, S2, and M1,* ***the NBG component corresponded to SPC2 in all cases****. Similarly,* ***across S1, S2, and M1, the BB component corresponded to SPC1 in all cases:***

Lines XXX-YYY: “*We identified NBG components using a run-length threshold applied independently to each spectral principal component (SPC) and electrode. For each SPC, we evaluated the frequency range 30–80 Hz (51 samples). An NBG component in a given electrode was marked present if this window contained a consecutive run spanning at least 75% of the window with magnitudes strictly greater than 0 (i.e., ≥ 39 consecutive samples within 30–80 Hz). Applying this criterion independently yielded a single NBG component in 4/7 electrodes in S1, 5/6 electrodes in S2, 63/63 electrodes in M1 (V1), and 0/63 electrodes in M1 (V4).* ***Across S1, S2, and M1, the NBG component corresponded to SPC2 in all cases****. Similarly, the BB components were selected with the same procedure over 1–200 Hz (200 samples). A BB component in a given electrode was marked present if this window contained a consecutive run spanning at least 75% of the window with magnitudes strictly greater than 0 (i.e., ≥150 consecutive samples within 1–200 Hz). Using this criterion, we found a single BB component in 7/7 electrodes in S1, 6/6 electrodes in S2, and 63/63 electrodes in M1 in both V1 and V4.* ***The BB component corresponded to SPC1 across S1, S2, and M1****. The average across channels for NBG and BB components following this procedure is depicted in* ***Figure S1****. The projection of SPC1 and SPC2 on the time-frequency axis for gratings stimuli for S1, S2, and M1 is shown in* ***Figure S2****.”*

7. In the legend to Figure S2 the authors describe a “fre covariance matrix”. What is “fre”? Later in the same legend a “compo frequency profile” is mentioned. What is “compo”?

Response: There is no such error in the Figure S2 caption.
